# Does It Bind? A Method to Determine the Affinity of Calcium and Magnesium Ions for Polymers Using ^1^H NMR Spectroscopy

**DOI:** 10.1021/acs.analchem.2c01166

**Published:** 2022-07-25

**Authors:** Matthew Wallace, Joshua Holroyd, Agne Kuraite, Haider Hussain

**Affiliations:** School of Pharmacy, University of East Anglia, Norwich Research Park, Norwich NR4 7TJ, U.K..

## Abstract

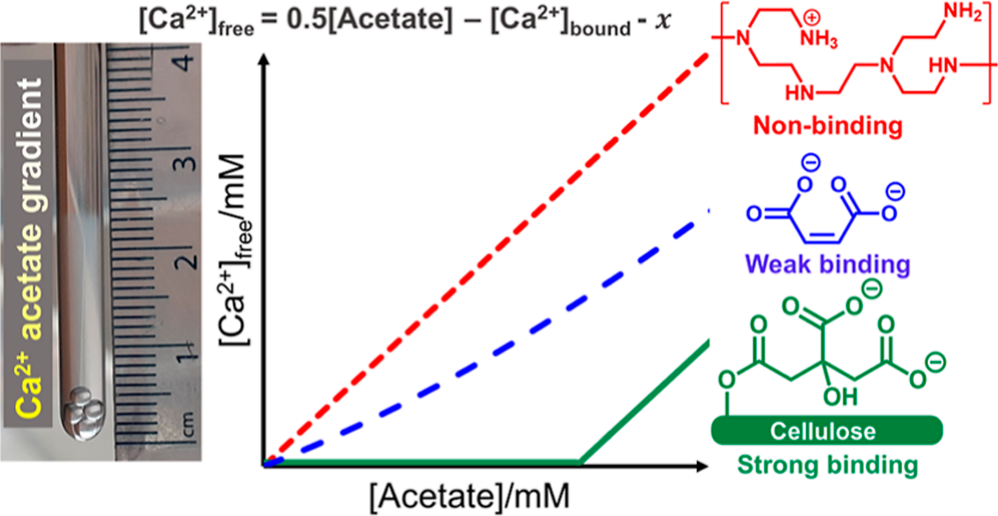

The binding of calcium and magnesium ions (M^2+^) by polymers and other macromolecules in aqueous solution is ubiquitous across chemistry and biology. At present, it is difficult to assess the binding affinity of macromolecules for M^2+^ without recourse to potentiometric titrations and/or isothermal titration calorimetry. Both of these techniques require specialized equipment, and the measurements can be difficult to perform and interpret. Here, we present a new method based on ^1^H NMR chemical shift imaging (CSI) that enables the binding affinity of polymers to be assessed in a single experiment on standard high-field NMR equipment. In our method, M^2+^ acetate salt is weighed into a standard 5 mm NMR tube and a solution of polymer layered on top. Dissolution and diffusion of the salt carry the M^2+^ and acetate ions up through the solution. The concentrations of acetate, [Ac], and free (unbound) M^2+^, [M^2+^]_f_, are measured at different positions along the sample by CSI. Binding of M^2+^ to the polymer reduces [M^2+^]_f_ and hinders the upward diffusion of M^2+^. A discrepancy is thus observed between [Ac] and [M^2+^]_f_ from which the binding affinity of the polymer can be assessed. For systems which form insoluble complexes with M^2+^, such as sodium polyacrylate or carboxylate-functionalized nanocellulose (CNC), we can determine the concentration of M^2+^ at which the polymer will precipitate. We can also predict [M^2+^]_f_ when a solution of polymer is mixed homogeneously with M^2+^ salt. We assess the binding properties of sodium polyacrylate, alginate, polystyrene sulfonate, CNC, polyethyleneimine, ethylenediamenetetraacetic acid, and maleate.

Many polymers will bind calcium and magnesium ions (M^2+^) in aqueous solution.^1−4^ Knowledge of the M^2+^-binding properties of polymers is vital when developing new materials or formulations. For example, the free concentration of M^2+^ must be carefully controlled when preparing media for the growth of cells.^5,6^ It is difficult to assess the M^2+^-binding strength of polymers using conventional titrimetric approaches. Potentiometric titrations require homogeneous mixing of the polymer and M^2+^ salt which can be difficult to achieve in systems exhibiting rapid aggregation upon contact with M^2+^.^7^ Furthermore, ion-sensitive electrodes require large volumes of sample (typically >10 mL) which may not be available when analyzing custom-synthesized materials.^4^ The electrodes also require extensive calibration before use and can suffer from artifacts due to the interaction of other sample components with the ion-sensitive membranes.^2^ Other approaches to assess the affinity of M^2+^ for polymers include the measurement of turbidity^1^ or the filtration of samples to remove insoluble complexes.^8^ However, these approaches provide only qualitative information and are limited to polymers that form insoluble aggregates upon binding to M^2+^. Isothermal titration calorimetry can be used to study ion–polymer association, but the data often requires additional analytical techniques for full interpretation.^2,8^ The gelation of alginate along concentration gradients of Ca^2+^ has been monitored using Ca^2+^-sensitive dyes,^9^ magnetic resonance imaging MRI,^10^ and dialysis.^11^ These approaches do not yield the concentrations of free and polymer-bound M^2+^ directly and are only suitable for polymers which form gels.

Here, we present a new method based on ^1^H NMR chemical shift imaging (CSI) that enables the binding affinity of polymers to be assessed without stirring or electrochemical probes. A full titration is performed in a single CSI experiment on one NMR sample with no adjustment required following preparation. The required NMR experiments can be performed under routine automation on standard NMR equipment, the total analysis time being less than 20 min.

In our method, solid M^2+^ acetate salt is weighed into a 5 mm NMR tube and an aqueous solution of the polymer placed on top. Dissolution and diffusion of the salt up the NMR tube establish concentration gradients of M^2+^ and acetate. Spatially resolved ^1^H NMR spectra are recorded at different vertical positions along the sample using CSI techniques.^12,13^ The concentration of free (unbound) M^2+^ ions, [M^2+^]_f_, at each position is determined from the ^1^H chemical shifts of the weakly complexing ligands, glycolate and sulfoacetate, as described in our previous work and Section S1 of the Supporting Information.^14^ The only calibration required is an ^1^H spectrum of these ligands in a solution of the polymer in the absence of M^2+^. The concentration of acetate at each position is determined by integration of the ^1^H NMR resonance of acetate against a reference such as dimethylsulfoxide (DMSO).

Once a gradient is established, the concentration of acetate will be higher toward the base of the NMR tube. We can thus measure [M^2+^]_f_ as a function of acetate concentration along our sample. With a nonbinding polymer, [M^2+^]_f_ will increase with acetate concentration as the upward diffusion of M^2+^ is unhindered. However, with a strong binding polymer, [M^2+^]_f_ will remain negligible until the binding sites on the polymer are occupied. A discrepancy is thus observed between the concentration of acetate and [M^2+^]_f_, provided other cations are present in the sample to diffuse with the acetate. A weakly binding polymer will show intermediate behavior. The binding strength of the polymer can thus be judged from a plot of [M^2+^]_f_*versus* acetate concentration (Scheme 1). We note that similar plots can be obtained by homogeneous mixing of the polymer with M^2+^ acetate, with [M^2+^]_f_ obtained from a conventional 1D ^1^H spectrum.^14^ However, this analysis must be repeated at several concentrations of M^2+^ acetate to elucidate the nature of the ion–polymer interaction. Homogeneous mixing also requires physical disruption of the gels that can form upon contact of polymers with M^2+^, potentially giving poor-quality NMR spectra. In this work, results obtained by homogeneous mixing of the polymer with M^2+^ acetate are compared with those obtained by analysis of M^2+^ gradients.

**Scheme 1:**
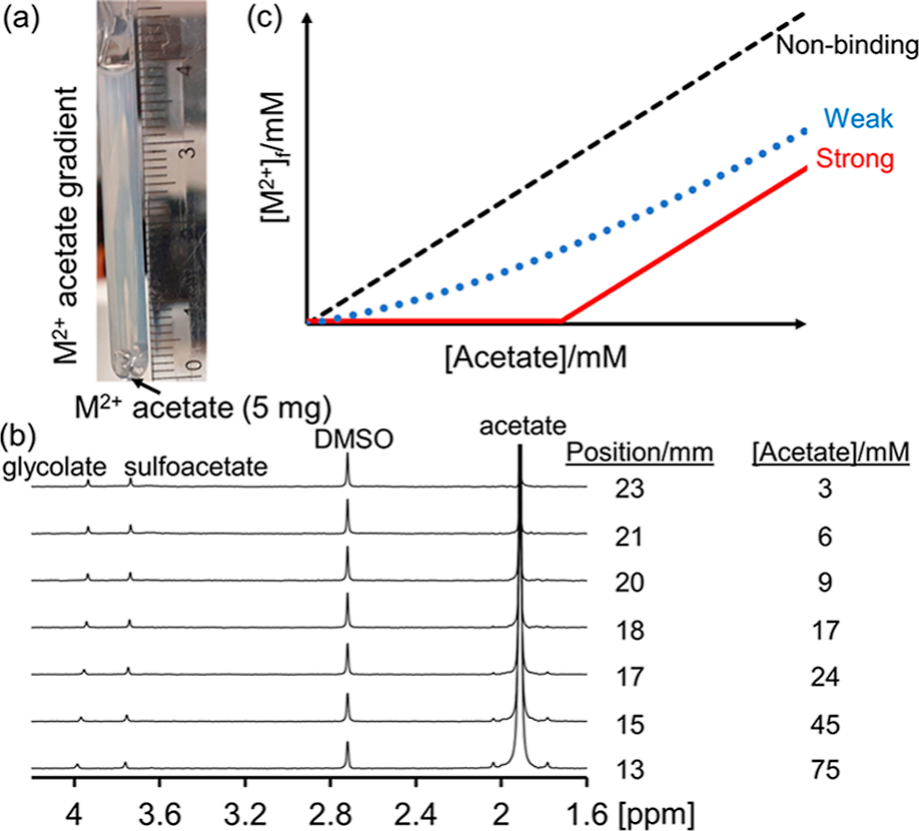
Method to Assess the Binding Affinity of M^2+^ for Polymers (a) A Concentration Gradient of M^2+^ Acetate Is Established by Layering a Solution of Polymer on Top of Solid Acetate Salt; (b) ^1^H NMR Spectra Are Recorded at Different Positions along the Sample using CSI Techniques; [M^2+^]_f_ and the Acetate Concentration Are Determined from These Spectra; and (c) Sketch of [M^2+^]_f_ versus Acetate Concentration for a Strong, Weak, and Nonbinding Polymer

We apply our method to assess the M^2+^-binding behavior of poly(sodium 4-styrenesulfonate) (PSS), sodium polyacrylate (PAA), alginate, polyethyleneimine (PEI), and citrate-functionalized nanocellulose (CNC). Good agreement is obtained with data published elsewhere, with our method correctly distinguishing between strong-binding (PAA, CNC, Ca-alginate), weak-binding (PSS, Mg-alginate), and nonbinding (PEI) systems. By monitoring the ^1^H resonances of polymers which form insoluble complexes with M^2+^, we can find the concentration of M^2+^ at which the polymers will fully precipitate from solution. We can also establish a lower limit for [M^2+^]_f_ when the polymer and M^2+^ salt are mixed homogeneously.

## Experimental Section

### Materials

All reagents were purchased from Merck or Fisher Scientific and used as received. Milli-Q water (18.2 MΩ cm) was used throughout the study. A 4 wt % stock dispersion of citrate-functionalized CNC at pH 7.4 was prepared as described in our previous work.^15^ The concentration of deprotonated carboxylate groups, [COO^–^], in a 1 wt % dispersion of CNC was determined as 3.1 ± 0.3 mM using our published method.^15^ Polyacrylic acid (*M*_w_ 240 kDa) was purchased from Fisher. PEI (branched, *M*_w_ 750 kDa), sodium alginate (viscosity of 1 wt % solution in H_2_O = 16 cps, mass loss on drying 13.7%), and PSS (*M*_w_ 70 kDa) were purchased from Merck. Tap water was obtained from a domestic supply in Norwich, UK. The alkalinity of the water was measured as 233 ± 6 mg/L CaCO_3_ using our published method.^15^

### Preparation of Samples

All samples were prepared in H_2_O with NaCl (0.05 M), DMSO (0.01 vol %), glycolate (1 mM), and sulfoacetate (1 mM). Na^+^ was the counterion in all cases. With the exception of the alginate and CNC samples, the following substances were also included: tert-butanol (0.01 vol %), 3-(trimethylsilyl)-1-propanesulfonic acid sodium salt (0.2 mM), and 2-methylimidazole (2MI, 1 mM). The pH of the samples was determined from the ^1^H chemical shift of 2MI (Section S2). The ethylenediaminetetraacetic acid (EDTA) sample was prepared with EDTA trisodium salt (EDTA-Na_3_, 5 mM) and 2MI (10 mM). The additional 2MI acted to absorb the proton that was liberated by the binding of M^2+^ to EDTA-Na_3_. The PAA sample was prepared at a concentration of carboxyl groups of 10 mM and was adjusted to pH 9.1 with NaOH. [COO^–^] was determined as 8.7 ± 0.6 mM,^15^ excluding glycolate and sulfoacetate, in agreement with titration data at pH 9 presented by Swift *et al.*(16) The PEI sample was prepared at 20 mM amine groups, assuming a monomer mass of 43 g/mol. The pH was adjusted to 8.8 by the addition of 0.2 equivalents of HCl. Based on data presented by Smits *et al.*,^17^ approximately 20% of the amine groups will be protonated at this pH. The PSS sample was prepared at pH 9.1 at a concentration of 10 mM sulfonate groups, assuming a monomer mass of 206 g/mol. The alginate samples were prepared from a 10 mg/mL stock solution of sodium alginate. [COO^–^] in the 4 mg/mL sample for NMR analysis was determined as 16.2 ± 0.8 mM, excluding glycolate and sulfoacetate.

To prepare gradients of M^2+^ acetate for analysis by CSI, 4–5 mg of solid calcium acetate hydrate or magnesium acetate tetrahydrate was loaded into the tip of a 9″ Pasteur pipette by pressing into the solid salt. The salt was then transferred from the tip to the base of a 5 mm NMR tube (Wilmad 528-PP). Four, 2 mm diameter glass beads were placed on top of the acetate salt. Prior to use, the beads were rinsed with ethanol and dried. The solutions, prepared as above, were carefully layered on top of the glass beads to a height of 40 mm from the base of the NMR tube with a 9″ Pasteur pipette. The samples were stood in the autosampler rack (22 °C) and analyzed by CSI every 2–4 h. The data presented in this paper was collected between 5 and 9 h after preparation. However, we note that useable data can be collected between 3 and 13 h after preparation (Section S3).

Homogeneous samples of the polymer and M^2+^ were prepared directly in NMR tubes by combining a stock solution of M^2+^ acetate with the polymer and additives listed above. The concentration of the M^2+^ acetate stock was verified by integration of the acetate resonance against 0.5 M potassium hydrogen phthalate in D_2_O. With the exception of the CNC samples, [M^2+^]_tot_ was based on the volume of acetate solution added and is assumed accurate to 3%. The CNC samples were prepared by addition of aliquots (<10 μL) of M^2+^ acetate solution to the CNC to conserve material and enable several values of [M^2+^]_tot_ to be measured with the same sample. [M^2+^]_tot_ in the CNC samples was determined from the ^1^H integral of acetate. The Ca^2+^-alginate and CNC samples were gently centrifuged (<500 rpm) on a Hettich 1011 hand centrifuge to drive solid material to the lower region of the tube. 0.5 wt % CNC samples in hard and soft water were prepared by combining CNC stock with tap and Milli-Q water in different proportions. No glycolate, sulfoacetate, NaCl, or DMSO was included in these samples.

### NMR Analysis

All experiments were performed off-lock in 100% H_2_O at 298 K on a Bruker 500 MHz AVANCE III spectrometer. ^1^H chemical shift images were acquired using a gradient-phase-encoding sequence based on Trigo-Mouriño *et al.*(12) The sequence incorporated a double echo excitation sculpting component (Bruker library zgesgp) for water suppression (Section S13.1). Gaussian inversion pulses of 4 ms duration and 300 Hz peak power were applied to the H_2_O resonance. The phase-encoding gradient pulse (172 μs) was in the form of a smoothed square and was ramped from −18.8 to 18.8 G/cm in 64 steps, giving a theoretical spatial resolution of 0.41 mm along the *z*-axis. Four scans were acquired at each gradient increment, with a signal acquisition time of 2 s and relaxation delay of 2 s. A spoil gradient (27 G/cm) was employed at the end of the acquisition period to destroy any remaining transverse magnetization. 16 dummy scans were run prior to acquisition, giving a total acquisition time of 19 min ^1^H spectra were acquired in 32 scans, 32 dummy scans, using the same excitation sculpting sequence and timings used for CSI but without the phase-encoding gradient.

### Data Analysis

Prior to preparing M^2+^ acetate gradients, an ^1^H spectrum of the samples was acquired to measure the ^1^H chemical shifts of glycolate and sulfoacetate in the absence of M^2+^.^14^ The lower detection limits of [M^2+^]_f_ are 0.4 and 0.8 mM for Ca and Mg, respectively, below which [M^2+^]_f_ can be taken as zero within the uncertainty of the measurement (Section S1). CSI data sets were processed in a phase-sensitive mode with 32K points and an exponential line broadening factor of 3 Hz. 1D spectra were processed with 64K points. Each row (64) of the CSI data set was automatically phase- and baseline-corrected using an automation script written in house. The ^1^H chemical shifts of DMSO, glycolate, sulfoacetate, and 2MI and the ^1^H integrals of DMSO and acetate were extracted from each row using a custom script. Tert-butanol was used as an alternative integral reference when DMSO overlapped with other resonances (EDTA, PEI). Lineshape deconvolution was used when acetate overlapped with resonances of the polymer (PSS and PAA). [Ac] was obtained as [Ac] = *kA*/*R*, where *A* and *R* are the integrals of acetate and reference, *k* = 2.92 (DMSO) and 3.83 (tert-butanol). All integration methods give equivalent results (Figure S1). All spectra were referenced to DMSO (2.72 ppm). Chemical shift and integral data were exported to the spreadsheet accompanying this work where referencing and data quality checks are performed automatically. To calculate *Z* (eq 3), the distance between the absolute base of the NMR tube and the center of the CSI image was determined as 19 mm by analysis of biphasic samples, as described in our previous work.^13^ Scripts for the automated acquisition and processing of CSI data sets are provided in Section S13.

## Results and Discussion

### Experimental Design and Validation Using Small-Molecule Ligands

If M^2+^ acetate is diffused into an aqueous solution that does not contain other ionic species, the concentrations of M^2+^ and acetate must remain in the ratio 1:2 to maintain electroneutrality. However, when other ionic species are present, the M^2+^ ions and acetate can diffuse at different rates—the salt effect.^18,19^ As all practical samples will contain other ionic species, we use 50 mM NaCl as a background medium to provide a constant salt effect and excess of monovalent cations (Section S8). The measured concentration of acetate, [Ac], is related to the concentrations of the M^2+^ species in the sample by eq 1
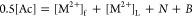
1where [M^2+^]_L_ is the concentration of M^2+^ bound to the glycolate, sulfoacetate, acetate, and chloride ligands. *N* is the salt effect, and *B* is the remaining discrepancy due to association of M^2+^ with the polymer. [M^2+^]_L_ is calculated using eq 2
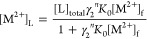
2where [L]_total_ is the total concentration of the ligand and *n* is the ligand charge (*n* = 1 for acetate, glycolate, and chloride; *n* = 2 for sulfoacetate). *K*_0_ is the binding constants for these ligands, provided in Section S4. γ_2_ is the activity coefficient of a divalent ion and can be calculated from the chemical shifts of glycolate and sulfoacetate as described in our previous work.^14^*N* may be calculated by assuming that the diffusion of M^2+^ and acetate follows separate Gaussian models (Section S5)
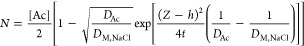
3where *D*_M,NaCl_ and *D*_Ac_ are the diffusion coefficients of the M^2+^ and acetate ions, respectively, measured in 50 mM NaCl in the absence of the polymer. *D*_Ac_ was obtained as 1 × 10^–9^ m^2^ s^–1^, and *D*_M,NaCl_ was obtained as 9.3 × 10^–10^ and 9.0 × 10^–10^ m^2^ s^–1^ for Ca^2+^ and Mg^2+^, respectively (Section S5). *Z* is the vertical distance from the absolute base of the NMR tube and is obtained directly from the chemical shift image (Equation S28). *t* is the time elapsed since preparation of the sample. *h* is the height of the solid acetate layer when prepared (2 mm). The stated diffusion coefficients are used to calculate *N* in all experiments as 50 mM NaCl is used as a constant background medium in this work. We recommend that *D*_Ac_ and *D*_M,NaCl_ are redetermined if an alternative background salt with different diffusion properties is used.^20^*B* may be obtained by rearrangement of eq 1
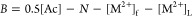
4

**Figure 1:**
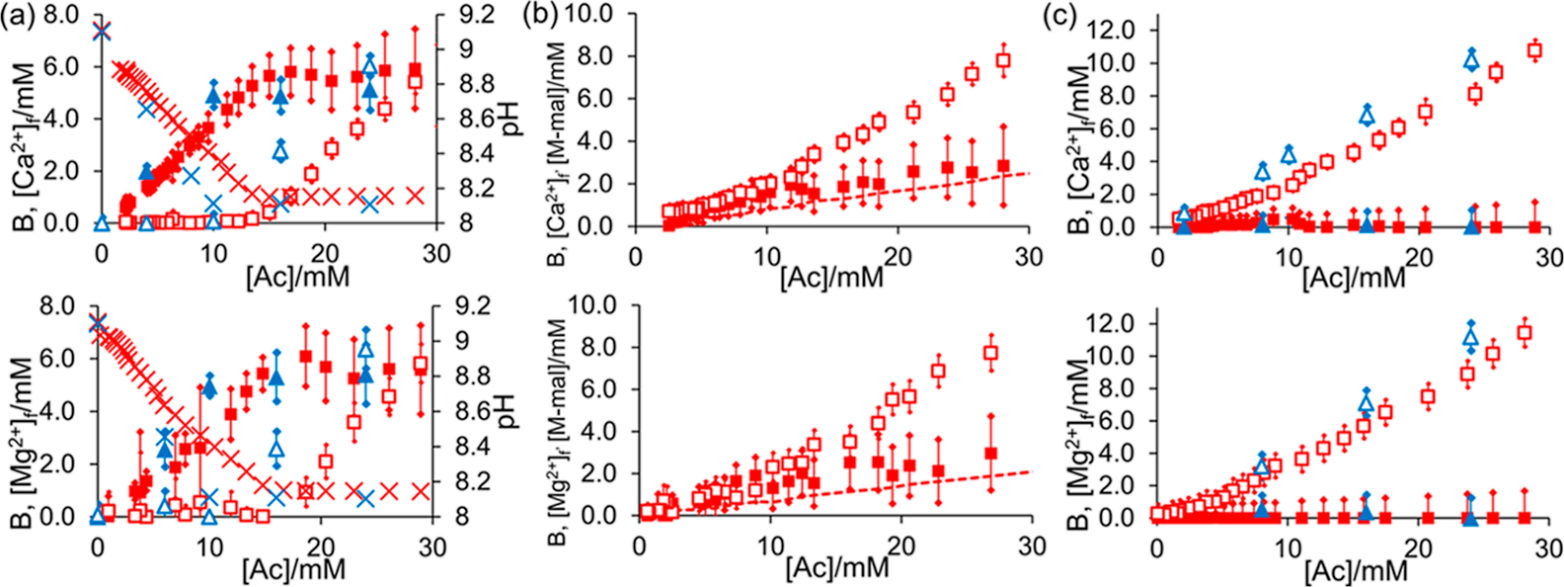
Plot of B (solid symbols) and [M^2+^]_f_ (open symbols) when calcium acetate (upper plots) or magnesium acetate (lower plots) is diffused into solutions of the ligand and the sample analyzed by CSI (red square) or mixed homogeneously with the ligand (blue triangle): (a) 5 mM EDTA, pH (cross); (b) 10 mM maleate, concentration of M^2+^-maleate complex, M-mal, calculated using eq 2 (dashed line); and (c) 50 mM NaCl. All samples contained 50 mM NaCl in addition to these substances (Experimental Section).

A positive value of *B* indicates association of M^2+^ with the polymer under investigation. Calculations of [M^2+^]_f_, [Ac], and *N* are performed automatically by the spreadsheet accompanying this work. When the polymer is mixed homogeneously with M^2+^ acetate, *N* is set to zero as no concentration gradients are present. To test our approach, samples were prepared containing 50 mM NaCl and the following ligands: 50 mM NaCl (100 mM NaCl total), 5 mM EDTA, and 10 mM disodium maleate (Experimental Section). Plots of B and [M^2+^]_f_*versus* [Ac] for these ligands are provided in Figure 1. EDTA exhibits strong binding behavior, with [M^2+^]_f_ remaining zero within the uncertainty of the measurement until B has attained a plateau. The binding of M^2+^ to EDTA liberates a proton which causes the pH of the sample to fall with acetate concentration until all the EDTA is complexed.

Complexation is also apparent from changes to the ^1^H NMR resonances of EDTA (Section S6). The increase in [M^2+^]_f_ thus coincides with full complexation of the EDTA. In contrast, no binding is observed to 50 mM NaCl in agreement with the low-stability constant of the M–Cl ion pair (log *K*_0_ < 1).^21^ [M^2+^]_f_ rises monotonically with acetate concentration, while B remains zero within the uncertainty of the measurement. The same binding behaviors are apparent by homogeneous mixing of EDTA or NaCl with M^2+^ acetate, although the CSI data is shifted to higher [Ac] due to the salt effect. Both methods demonstrate that EDTA is a strong binder and NaCl is a nonbinder. Maleate (log *K*_0_ = 2.40 for Ca^2+^, 2.30 for Mg^2+^) is a much weaker binder than EDTA.^21^ The unbound maleate ligand can thus coexist with millimolar concentrations of free M^2+^ (eq 2). Accordingly, both B and [M^2+^]_f_ increase with acetate concentration. B remains far below the stoichiometric requirement of the ligand (10 mM), confirming weak binding.

Our CSI method can thus distinguish between strong, weak, and nonbinding ligands. However, it is apparent from the EDTA data (Figure 1a) that our method does not yield the exact 1:1 binding stoichiometry observed by homogeneous mixing of EDTA and M^2+^ acetate. We note that the binding of M^2+^ to a polymer or ligand will reduce *D*_M_ (Equation 3) below the value measured in 50 mM NaCl. The actual concentration of M^2+^ bound to the polymer or ligand, [M^2+^]_b_, is thus always less than or equal to *B*, provided *N* ≥ 0 (Section S7).

### Analysis of Binding Properties of PSS, PAA, and PEI

**Figure 2:**
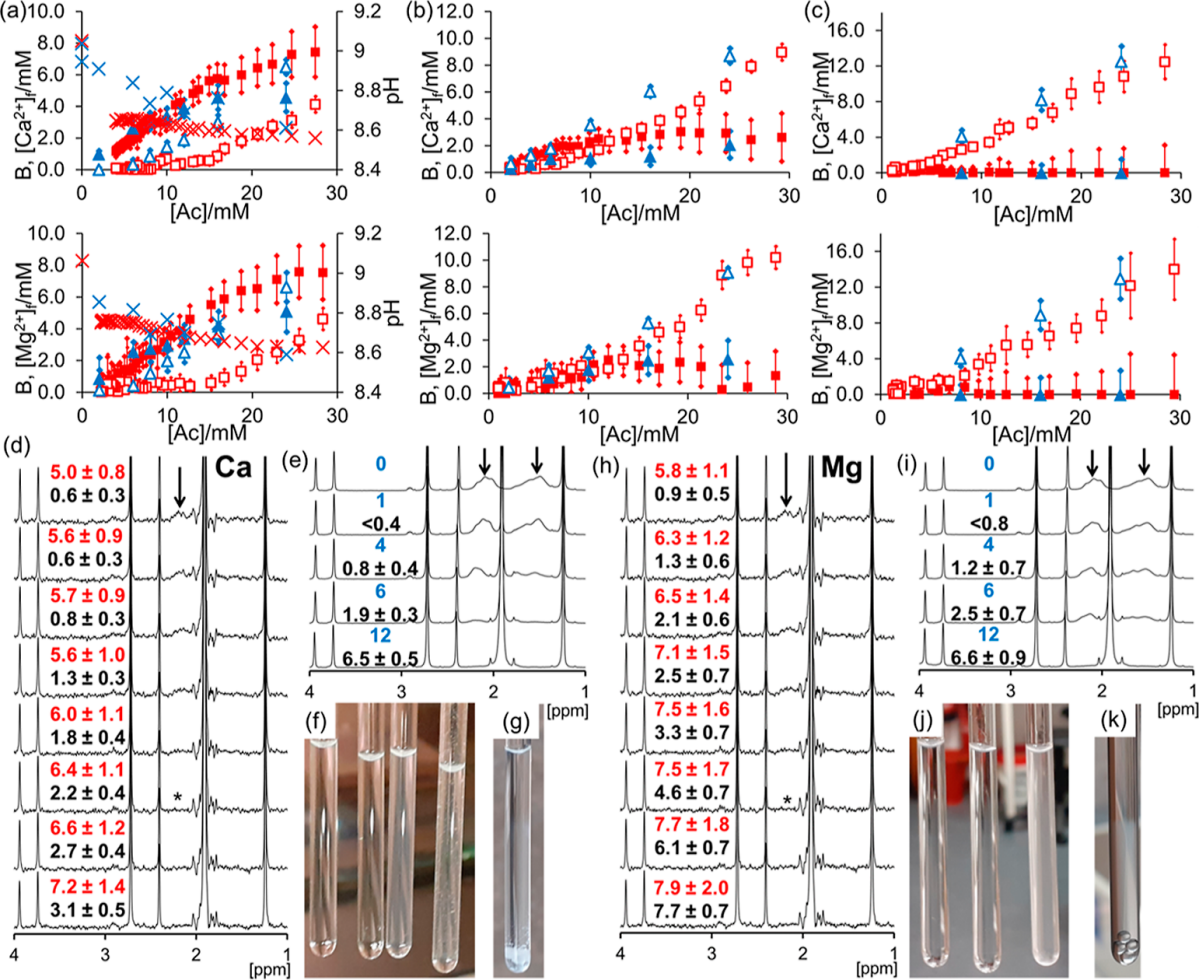
(a–c): Plot of B (solid symbols) and [M^2+^]_f_ (open symbols) when calcium acetate (upper plots) or magnesium acetate (lower plots) is diffused into solutions of the polymer and the sample analyzed by CSI (red square) or mixed homogeneously with the polymer (blue triangle): (a) PAA, pH (cross); (b) PSS; and (c) PEI. (d–k): ^1^H spectra and photographs of PAA samples with Ca^2+^ (d–g) and Mg^2+^ (h–k). (d,h): ^1^H spectra extracted from CSI data set. ^1^H resonances of PAA are indicated with arrows. * indicates the critical spectrum where the PAA resonances are not visible. *B*/mM (red) and [M^2+^]_f_/mM (black). (e,i): ^1^H spectra of homogeneous samples of PAA and M^2+^. [M^2+^]_tot_/mM (blue) and [M^2+^]_f_/mM (black). [M^2+^]_f_ is below the lower detection limits when [M^2+^]_tot_ = 1 mM ([Ac] = 2 mM). Photographs of homogeneous samples: from left to right, (f) [Ca^2+^]_tot_: 0, 4, 6, 12 mM and (g) Ca CSI sample 23 h after preparation. (j) [Mg^2+^]_tot_: 4, 6, and 12 mM and (k) Mg CSI sample 16 h after preparation.

Having validated our method on small-molecule ligands, we now assess the M^2+^-binding properties of the water-soluble synthetic polymers PAA, PSS, and PEI. Plots of *B* and [M^2+^]_f_*versus* [Ac] are provided for each polymer in Figure 2a–c. PAA and PSS are both anionic polyelectrolytes with average axial charge spacings of 2.9 and 2.5 Å, respectively. Both polymers can be expected to exhibit counterion condensation.^4,22^ The partially cationic PEI exhibits negligible binding to M^2+^, with *B* remaining zero within the uncertainty of the measurement. With PSS, *B* attains a plateau at 2 mM, far below the stoichiometric requirement of 5 mM where two sulfonate groups would be coordinated to one M^2+^ ion. After the plateau is attained, [M^2+^]_f_ increases with acetate concentration. Additional plots are provided in Figure S8. Our results are in good agreement with potentiometry data presented by Ostrowska-Czubenko,^4^ who observed that [M^2+^]_b_ attained a plateau when the ratio of Ca^2+^ to sulfonate groups on PSS reached 0.2. This plateau is predicted by the counterion condensation theory, where M^2+^ would condense onto PSS until a partial neutralization of the negative charge had been obtained.^22^ The ^1^H NMR resonances of PEI and PSS do not decrease in intensity as the concentration of M^2+^ is increased, confirming that the polymers remain mobile in solution (Section S6). Plots of [M^2+^]_f_ and *B versus* [Ac] obtained by homogeneous mixing of PSS or PEI with M^2+^ acetate are in qualitative agreement with those obtained by CSI (Figure 2a–c).

PAA exhibits stronger binding behavior than PSS. [M^2+^]_f_ remains negligible (<1 mM) until *B* has exceeded the stoichiometric requirement of 4.4 ± 0.3 mM, where two carboxylate groups on PAA would be bound to one M^2+^ ion. We note that *B* can exceed the stoichiometric requirement due to exchange between free and polymer-bound M^2+^ (Section S7). The increase in [M^2+^]_f_ above 2 mM coincides with the disappearance of the ^1^H resonances of PAA, implying a loss of mobility of the polymer chain and precipitation (Figure S3). Sinn *et al.*(2) and Siew *et al.*(3) performed titrations of PAA using Ca^2+^ ion-sensitive electrodes and observed that [Ca^2+^]_f_ remained negligible (<1 mM) until the ratio of M^2+^ to carboxylate groups on the PAA, *r*, exceeded approximately 0.2–0.3. Precipitation of the polymer was observed by these authors upon further addition of Ca^2+^, the critical value of r required to induce precipitation depending on the concentration of PAA. Similarly, Satoh *et al.*(23) demonstrated a strong interaction of PAA with Mg^2+^ and with Ca^2+^ in the presence of 50 mM NaCl, the activity of M^2+^ remaining negligible until *r* > 0.2.

A similar critical ratio is observed by homogeneous mixing of PAA with M^2+^ acetate, with [M^2+^]_f_ remaining negligible until [Ac] > 6 mM (*r* > 0.3), Figure 2a. We note that in the homogenous samples, *B* = [M^2+^]_b_, allowing direct measurement of [M^2+^]_f_ at different total ratios of M^2+^ to polymer. However, such analysis requires the preparation and analysis of a significant number of separate NMR samples (seven on Figure 2a). These samples require a greater quantity of the polymer and a longer total preparation and analysis time relative to the faster but more qualitative CSI experiment.

We can use our CSI data to predict the stability of PAA in solution when mixed homogeneously with M^2+^. By CSI, we can find the critical point along an M^2+^ acetate gradient where the ^1^H signals of the polymer are lost. As *B* ≥ [M^2+^]_b_, our method predicts that the polymer will fully precipitate from a homogeneously mixed solution if the total concentration of M^2+^, [M^2+^]_tot_, equals or exceeds the sum of [M^2+^]_f_, *B*, and [M^2+^]_L_ at this critical point in the CSI experiment. Similarly, our method predicts that the polymer will be stable if [M^2+^]_tot_ ≤ [M^2+^]_f_ at the critical point. We note that [M^2+^]_L_ is negligible (<1 mM) in our experiments and can be ignored in our prediction of stability as it is within the combined uncertainty of B and [M^2+^]_f_ (Section S9). In the CSI data set, the ^1^H resonances of the PAA disappear completely when the sum of *B* and [M^2+^]_f_ is 8.6 ± 1.5 mM for Ca and 12.1 ± 2.4 mM for Mg (Figure 2d,h). Accordingly, in the samples prepared by homogeneous mixing, the ^1^H resonances of PAA are not discernible with either Ca or Mg when [M^2+^]_tot_ = 12 mM, indicating that full precipitation has occurred (Figure 2e,i). Precipitation of the polymer is visually apparent (Figure 2f,j). The Ca sample was initially turbid but cleared on standing (<10 min) with the formation of macroscopic aggregates, as photographed. Precipitation was also apparent in the samples prepared for analysis by CSI (Figure 2g,k). Our method predicts that PAA will be stable in solution when [M^2+^]_tot_ is less than 2.2 ± 0.4 mM for Ca and 4.6 ± 0.7 mM for Mg. Accordingly, in the homogeneous samples, the ^1^H resonances of PAA at [M^2+^]_tot_ = 1 mM (*r* = 0.11) are very similar in shape and intensity to the resonances observed in the absence of M^2+^, confirming that no significant precipitation of the polymer has occurred (Figure 2e, i). As [M^2+^]_tot_ is increased up to 6 mM, the ^1^H resonances of PAA decrease in intensity but do not disappear completely, indicating that mobile polymer is still present. A full spectral assignment is provided in Figure S3.

**Figure 3:**
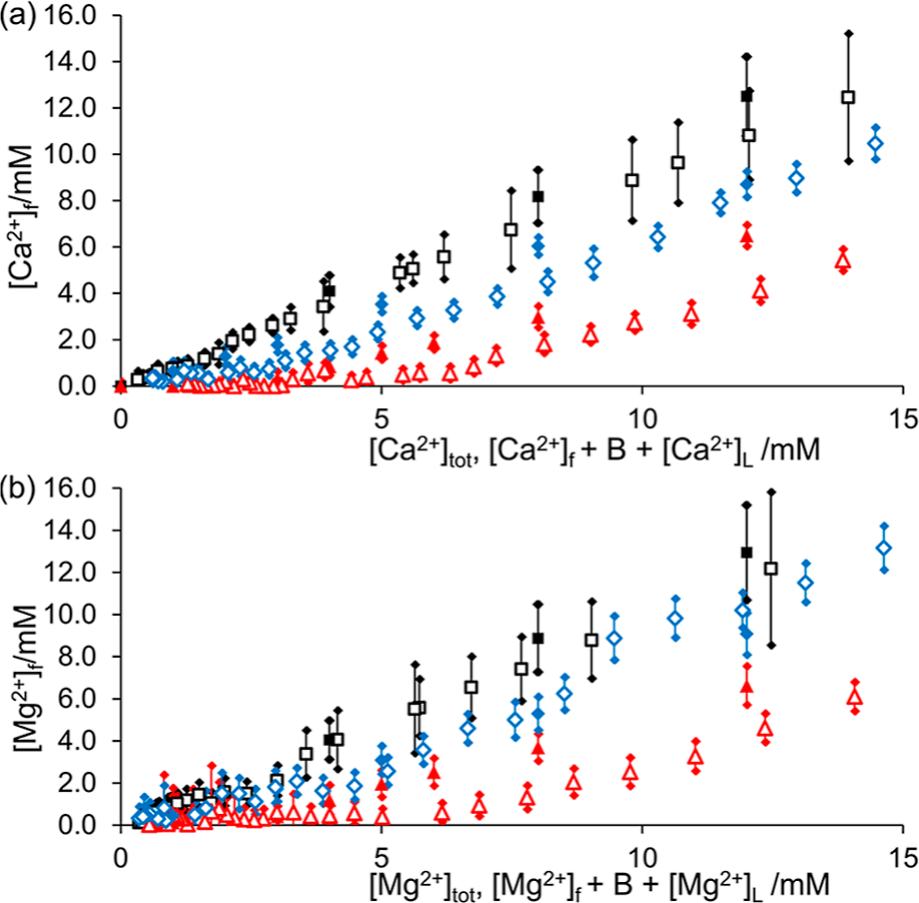
Plot of [M^2+^]_f_ measured by CSI along an M^2+^ acetate gradient *vs* [M^2+^]_f_ + *B* + [M^2+^]_L_ (open symbols) and [M^2+^]_f_ in homogeneous samples *vs* [M^2+^]_tot_ (solid). PEI (black square), PSS (blue rhombus), and PAA (red triangle). (a) Ca and (b) Mg.

We can also use the CSI data to determine a lower limit for [M^2+^]_f_ when a polymer is mixed homogeneously with M^2+^. As *B* ≥ [M^2+^]_b_, a homogeneous sample was prepared so that [M^2+^]_tot_ = [M^2+^]_f_ + *B* + [M^2+^]_L_ will possess a free ion concentration equal to or greater than the value of [M^2+^]_f_ measured in the CSI experiment. Comparing the CSI data from Figure 2 with [M^2+^]_f_ measured in homogeneous samples, this prediction is correct (Figure 3).

The ability to predict a lower limit for [M^2+^]_f_ could be useful where it is necessary to maintain [M^2+^]_f_ below certain levels, for example, in the preparation of cell culture media^5,6,24^ or when investigating drug–protein binding.^25^

### Analysis of Binding Properties of Alginate and CNC

Finally, we consider the interaction of M^2+^ with alginate and citrate-functionalized CNC. Carboxylate-functionalized CNCs have been proposed for a wide variety of applications in biomedicine,^26^ foods,^27^ and wastewater treatment.^28^ In all these applications, the colloidal stability, M^2+^-binding ability, and [M^2+^]_f_ are important factors to consider when designing CNC formulations. Calcium alginate gels are of practical relevance for cell-culturing applications, where the concentration of alginate and [Ca^2+^]_tot_ can be used to vary the mechanical and biological properties of the materials.^29^ [Ca^2+^]_f_ will determine the growth and viability of the cells;^5^ however, it is difficult to measure [M^2+^]_f_ in intact gels using potentiometric methods.^7^

**Figure 4:**
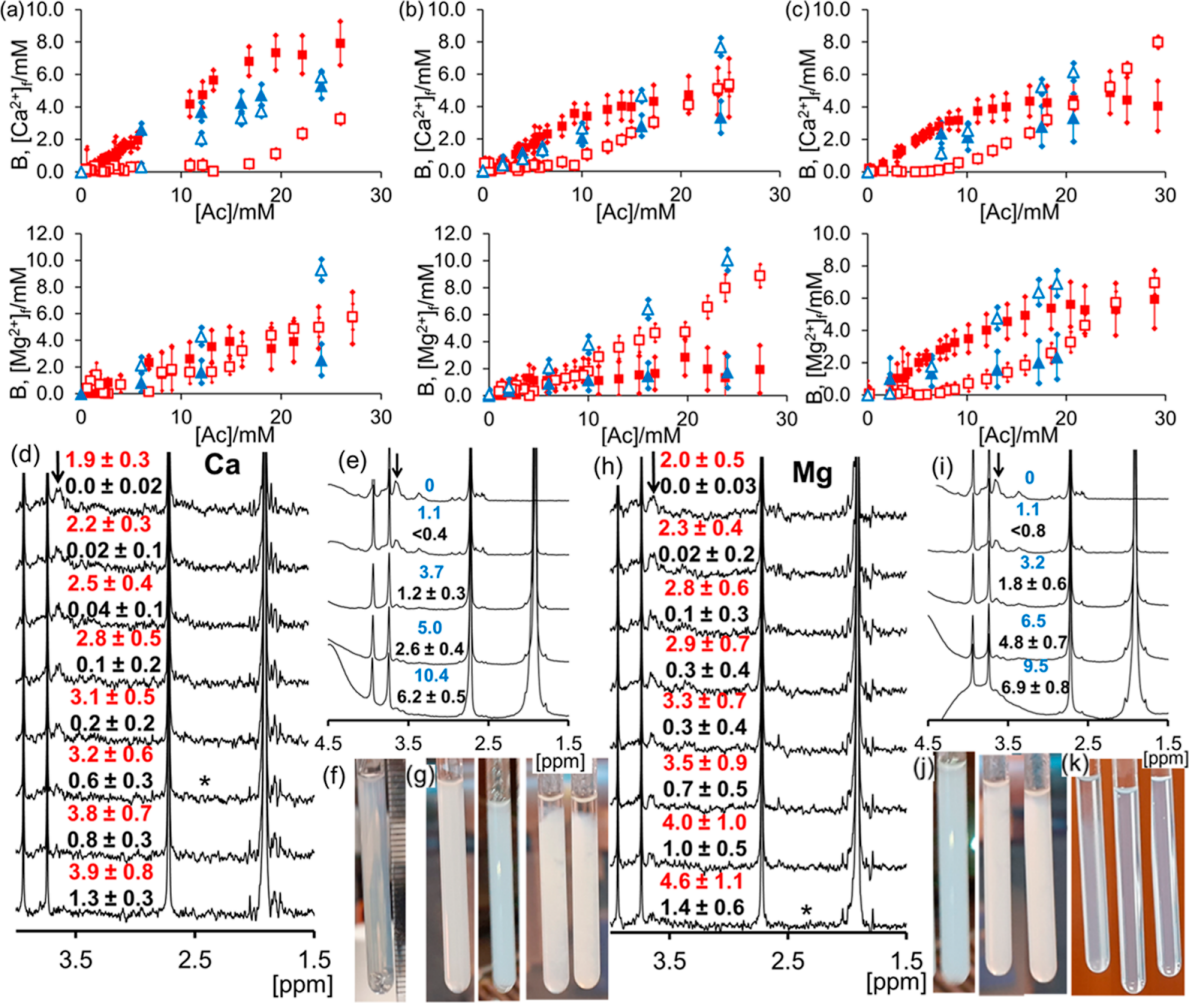
(a–c): Plot of *B* (solid symbols) and [M^2+^]_f_ (open symbols) when calcium acetate (upper plots) or magnesium acetate (lower plots) is diffused into solutions of alginate or CNC and the sample analyzed by CSI (red square) or mixed homogeneously (blue triangle): (a) 4 mg/mL alginate, (b) 2 mg/mL alginate, and (c) 2 wt % citrate-CNC. (d–k): ^1^H spectra and photographs of CNC samples with Ca^2+^ (d–g) and Mg^2+^ (h–k). (d,h): ^1^H spectra extracted from CSI data set. ^1^H resonances of CNC are indicated with arrow. * indicates the spectrum at which the resonances are no longer visible. B/mM (red) and [M^2+^]_f_/mM (black). (e,i): ^1^H spectra of homogeneous samples. [M^2+^]_tot_/mM (blue) and [M^2+^]_f_/mM (black). [M^2+^]_f_ is below the lower detection limits when [M^2+^]_tot_ = 1.1 mM. (f) Ca CSI sample 46 h after preparation. (g,j): Photographs of homogeneous samples. From left to right, (g) [Ca^2+^]_tot_: 0, 1.1, 3.7, and 5.0 mM and (j) [Mg^2+^]_tot_: 1.1, 3.2, and 6.5 mM. (k) 0.5 wt % CNC in tap water. From left to right: 190 ± 5, 39 ± 1, and 0 mg/L CaCO_3_.

Data for alginate at 2 and 4 mg/mL and 2 wt % citrate-functionalized CNC are presented in Figure 4. A strong interaction between Ca^2+^ and sodium alginate is apparent by CSI (Figure 4a,b). [Ca^2+^]_f_ rises above 1 mM only after B has approached the stoichiometric requirement of 8.1 ± 0.4 and 4.0 ± 0.2 mM for 4 and 2 mg/mL sodium alginate, respectively. A much weaker interaction is detected between alginate and Mg^2+^. [Mg^2+^]_f_ increases with [Ac], while *B* remains far below the stoichiometric requirement. The same behavior is apparent by homogeneous mixing of M^2+^ acetate and alginate. With Ca^2+^, B ≥ [Ca^2+^]_f_ when [Ac] < 10 mM ([Ca^2+^]_tot_ < 5 mM). For Mg^2+^, *B* < [Mg^2+^]_f_ throughout the titration, confirming a weaker interaction of Mg^2+^ with the alginate. Our results are consistent with the observation that alginate forms strong gels upon addition of Ca^2+^ due to strongly site-bound ions but does not form gels with Mg^2+^ due to a more diffuse ion–polymer interaction.^8,30,31^ The average axial charge spacing of alginate is approximately 4.7 Å, so counterion condensation of Mg^2+^ onto the alginate is expected.^3,30^ Direct mixing of Ca^2+^ and alginate results in the formation of gel particles. No particles are observed when Mg^2+^ and alginate are mixed (Figure S25).

The citrate-functionalized CNC exhibits a strong interaction with Ca^2+^ and Mg^2+^ (Figure 4c). With both ions, [M^2+^]_f_ increases above 1 mM only after *B* has exceeded the stoichiometric requirement of 3.1 ± 0.3 mM. Measurements of optical transmittance indicate a similar onset of aggregation for Ca^2+^ and Mg^2+^ when titrated with M^2+^, the samples becoming essentially opaque when [M^2+^]_tot_ exceeds 3 mM (Section S11). A strong interaction of Ca^2+^ and Mg^2+^ with citrate-CNC is also apparent by homogeneous mixing of CNC with M^2+^ acetate. With both ions, *B* ≥ [M^2+^]_f_ when [Ac] < 6 mM. We note that the different preparation methods of the CNC-M^2+^ samples (direct mixing *vs* slow diffusion) may contribute to the differences observed between the plots of *B versus* [Ac] for the CSI and mixed data sets. Nevertheless, the two methods are in qualitative agreement. The detection of a strong interaction by our CSI method indicates that a homogeneous solution prepared so that *r* < 0.5 will have [M^2+^]_f_ ≪ [M^2+^]_tot_ as the majority of M^2+^ in the sample will be complexed to the polymer. Similarly, a sample prepared with *r* ≫ 0.5 will have a significant [M^2+^]_f_ which will increase almost linearly with [M^2+^]_tot_ (Section S12). Our citrate-CNC has a carboxyl content of 0.31 mmol/g. Approximately, 3% of glucose units in our CNC thus bear a doubly deprotonated citrate moiety.^15^ Depending on the distribution of these groups on the CNC surface, the average spacing between carboxylate groups may drop below the critical value of 7.1 Å required for counterion condensation to occur.^22^ Site-specific binding to the citrate moiety may also take place.^14^ We note that the nature of CNC surfaces is currently an active area of research.^32^

A strong affinity of Mg^2+^ and Ca^2+^ for carboxylate-functionalized CNCs was observed by Lombardo *et al.*,^33^ who studied the salt-induced aggregation of CNCs prepared *via* TEMPO-mediated oxidation. These authors found that the critical concentrations of MgCl_2_ and CaCl_2_ required to induce aggregation of the CNCs were the same within experimental error. These authors also observed similar Gibbs energies of ion absorption and stoichiometric coefficients for both ions.

In the absence of M^2+^, citrate-functionalized CNC possesses mobile chains on the surface of the CNC particles which can be observed by solution-state ^1^H NMR.^15,34^ The ^1^H resonances of the CNC disappear from the CSI spectra (Figure 4d,h) when the sums of B and [M^2+^]_f_ are 3.8 ± 0.9 mM for Ca and 6.0 ± 1.7 mM for Mg. In accord with our predictive method, aggregation of the CNC and loss of the ^1^H NMR resonances are observed in samples prepared by direct mixing of M^2+^ acetate and CNC when [M^2+^]_tot_ exceeds these values (Figure 4e,g,i,j). The baselines of these spectra are distorted due to the presence of solid aggregates in the sample tube. These distortions are not apparent in the CSI data as mechanical mixing and breakage of the gels were not performed (Figure 4f). Larger-scale CSI spectra are provided in Section S10, along with 2D overview plots. The high sensitivity of citrate-functionalized CNC to M^2+^ revealed by our method could be of relevance for the practical application of the CNCs. For example, the concentration of M^2+^ in drinking water can exceed 2 mM in many parts of the World.^15,35^ A 0.5 wt % dispersion of citrate-functionalized CNC ([COO^–^] = 1.5 ± 0.2 mM) contains solid aggregates if prepared in hard water (190 ± 5 mg/L CaCO_3_) but remains stable in soft water (39 ± 1 mg/L CaCO_3_), Figure 4k. This spontaneous aggregation upon exposure to natural waters could be of significance when considering the use of CNCs as flocculants.^28^

## Conclusions

The affinity of polymers for Ca^2+^ and Mg^2+^ (M^2+^) is an important consideration when designing systems that will function in the presence of these ions. Our method provides a convenient indication of the M^2+^-binding strength of a polymer and a prediction of the stability and free ion concentration when the polymer and M^2+^ salt are mixed homogeneously. If sufficient sample is available, these predictions can be confirmed by direct mixing, with the concentration of unbound M^2+^ ions determined from a ^1^H NMR spectrum. Our method requires an ^1^H chemical shift image of a sample containing an M^2+^ acetate gradient, along with a ^1^H reference spectrum. If the reference sample is subsequently used to create the gradient, the total volume of the sample required is less than 0.7 mL. These modest requirements make the method suitable for analysis of biomolecules or custom-synthesized materials, where only small quantities of the sample may be available. Our method would allow optimization of the concentration and type of charged groups on a polymer or gel to obtain the desired binding properties and stability. In addition to the ^1^H spectra required by our method, we note that other position-selective NMR experiments such as ^23^Na or relaxation measurements could be performed on the same sample to gain further insights into ion displacement phenomena or the mobility of polymer chains.

## Supporting Information Available

Estimation of uncertainty in B and [M^2+^]_f_; determination of pH; CSI analysis at different times since sample preparation; ligand-binding constants and chemical shifts; derivation of eq 3; ^1^H spectra of PSS, PEI, and EDTA; interpretation of plots of B versus [Ac]; effect of 50 mM NaCl on plot of B *versus* [Ac]; plots of [M^2+^]_L_*versus* [Ac]; expanded plots from CSI analysis of alginate and CNC; optical transmittance of CNC samples; prediction of [M^2+^]_f_ in alginate and CNC samples using CSI data; and pulse program and processing scripts (PDF)
Spreadsheet for analysis of CSI data sets (XLSX)
