# Supplementary material for: Does It Bind? A Method to Determine the Affinity of Calcium and Magnesium Ions for Polymers Using 1H NMR Spectroscopy
Source: Anal Chem. 2022 Jul 25;94(31):10976–83. doi: 10.1021/acs.analchem.2c01166 (PMC9366736; doi:10.1021/acs.analchem.2c01166)
Supplement: Supplementary file 1 — ac2c01166_si_001.pdf [file ac2c01166_si_001.pdf]

Supporting Information for:

Does it bind? A method to determine the affinity of calcium and magnesium ions for polymers using ^{1}H NMR spectroscopy

Matthew Wallace,* Josh Holroyd, Agne Kuraite and Haider Hussain

School of Pharmacy, University of East Anglia, Norwich Research Park, Norwich, NR4 7TJ, UK

*Corresponding Author: Matthew Wallace, email: matthew.wallace@uea.ac.uk

Contents

S1. Estimation of uncertainty in the determination of $[M^{2+}]_f$ and B, with spreadsheet calculation 2

S2. Determination of pH from ^{1}H chemical shift of 2-methylimidazole 5

S3. CSI analysis of M^{2+} gradients at different times since preparation 7

S4. Ligand binding constants and limiting chemical shifts 12

S5. Salt effect on diffusion of M^{2+} and acetate, derivation of Equation 3 13

S6. ^{1}H spectra of PSS, PEI and EDTA 16

S7. Interpretation of plots of B and $[M^{2+}]_f$ versus [Ac] 19

S8. Effect of 50 mM NaCl on the binding behaviour observed by CSI 22

S9. Plots of $[M^{2+}]_L$ versus [Ac] for samples of Figures 2 and 4 23

S10. Expanded plots from CSI datasets of citrate-functionalised CNC and 4 mg/mL sodium alginate, ^{1}H spectra of 0.5 wt% CNC in tap water 24

S11. Optical transmittance of CNC samples at 600 nm 29

S12. Prediction of $[M^{2+}]_f$ in homogeneous samples of alginate and citrate-CNC using CSI data 30

References 30

S13. Pulse programs and AU scripts 31

S13.1 2D pulse sequence for CSI (Bruker) 31

S13.2 AU program to process raw CSI datasets (Bruker) 32

S13.3 AU program to phase and baseline correct CSI datasets (Bruker) 33

S13.4 AU program to extract chemical shifts from CSI dataset (Bruker) 34

S13.5 AU program to extract integrals of acetate and reference from a CSI dataset (Bruker) 37

S13.6 AU program to extract areas of acetate and reference from a CSI dataset by lineshape deconvolution (Bruker) 40

S13.7 AU program to shim and find water suppression frequency when running under IconNMR (Bruker) 42

S13.8 Processing procedure for Mnova 14.2.0 43

S1. Estimation of uncertainty in the determination of $[M^{2+}]_f$ and B, with spreadsheet calculation

$[M^{2+}]_f$ and γ_2 are obtained using Equations S1 and S2, respectively, as in our previous work:[50]

$$[M^{2+}]_f = \frac{(\delta_{L1} - \delta_{M1})(\delta_{a1} - \delta_{b1})}{K_{0,1}\gamma_2(\delta_{b1} - \delta_{M1})(\delta_{a1} - \delta_{M1})} \quad \text{S1}$$

$$\gamma_2 = \frac{K_{0,1}(\delta_{L2} - \delta_{M2})(\delta_{a2} - \delta_{b2})(\delta_{a1} - \delta_{M1})(\delta_{b1} - \delta_{M1})}{K_{0,2}(\delta_{b2} - \delta_{M2})(\delta_{a2} - \delta_{M2})(\delta_{L1} - \delta_{M1})(\delta_{a1} - \delta_{b1})} \quad \text{S2}$$

where the subscripts 1 and 2 indicate glycolate and sulfoacetate, respectively. δ_M and δ_L are the limiting chemical shifts of the free and fully complexed ligands (Section S4). δ_a is the chemical shift measured in the polymer solution in the absence of M^{2+}, δ_b is the chemical shift measured in the presence of M^{2+} from the spectrum of interest. In the CSI experiment, δ_b is measured for glycolate and sulfoacetate at different positions along the M^{2+} gradient. δ_M and δ_L are assumed constant.

The uncertainty in γ_2, $\Delta\gamma_2$, is estimated by comparing the value obtained from Equation S3 with the value obtained from the reported $[M^{2+}]_f$:[50]

$$\Delta\gamma_2 = \gamma_2 - 10^{-2.04\left[\frac{\sqrt{3[M^{2+}]_f + I_0}}{1+\sqrt{3[M^{2+}]_f + I_0}} - 0.3(3[M^{2+}]_f + I_0)\right]} \quad \text{S3}$$

where I_0 is the ionic strength of the sample prior to addition of M^{2+}. The uncertainty in $[M^{2+}]_f$, $\Delta[M^{2+}]_f$, is obtained by propagation of uncertainty analysis of Equation S1:

$$\Delta[M^{2+}]_f = \sqrt{\left(\frac{\partial[M^{2+}]_f}{\partial K_{1,0}}\right)^2 \Delta K_{1,0}{}^2 + \left(\frac{\partial[M^{2+}]_f}{\partial \gamma_2}\right)^2 \Delta\gamma_2{}^2 + \left(\frac{\partial[M^{2+}]_f}{\partial \delta_{L1}}\right)^2 \Delta\delta_{L1}{}^2 + \left(\frac{\partial[M^{2+}]_f}{\partial \delta_{a1}}\right)^2 \Delta\delta_{a1}{}^2 + \left(\frac{\partial[M^{2+}]_f}{\partial \delta_{b1}}\right)^2 \Delta\delta_{b1}{}^2 + \left(\frac{\partial[M^{2+}]_f}{\partial \delta_{M1}}\right)^2 \Delta\delta_{M1}{}^2} \quad \text{S4}$$

where Δ indicates the uncertainty in the variable.

Differentiating Equation S1 with respect to each variable:

$$\frac{\partial[M^{2+}]_f}{\partial K_{1,0}} = -\frac{(\delta_{L1} - \delta_{M1})(\delta_{a1} - \delta_{b1})}{K_{1,0}^2\gamma_2(\delta_{b1} - \delta_{M1})(\delta_{a1} - \delta_{M1})} \quad \text{S5}$$

$$\frac{\partial[M^{2+}]_f}{\partial \gamma_2} = -\frac{(\delta_{L1} - \delta_{M1})(\delta_{a1} - \delta_{b1})}{K_{1,0}\gamma_2^2(\delta_{b1} - \delta_{M1})(\delta_{a1} - \delta_{M1})} \quad \text{S6}$$

$$\frac{\partial[M^{2+}]_f}{\partial \delta_{L1}} = \frac{(\delta_{a1} - \delta_{b1})}{K_{1,0}\gamma_2(\delta_{b1} - \delta_{M1})(\delta_{a1} - \delta_{M1})} \quad \text{S7}$$

$$\frac{\partial[M^{2+}]_f}{\partial \delta_{a1}} = \frac{(\delta_{b1} - \delta_{M1})(\delta_{L1} - \delta_{M1})[(\delta_{a1} - \delta_{M1}) - (\delta_{a1} - \delta_{b1})]}{K_{1,0}\gamma_2(\delta_{b1} - \delta_{M1})^2(\delta_{a1} - \delta_{M1})^2} \quad \text{S8}$$

$$\frac{\partial[\mathrm{M}^{2+}]_\mathrm{f}}{\partial\delta_{\mathrm{b1}}} = -\frac{(\delta_{\mathrm{a1}}-\delta_{\mathrm{M1}})(\delta_{\mathrm{L1}}-\delta_{\mathrm{M1}})[(\delta_{\mathrm{b1}}-\delta_{\mathrm{M1}})+(\delta_{\mathrm{a1}}-\delta_{\mathrm{b1}})]}{\mathrm{K}_{1,0}\gamma_2(\delta_{\mathrm{b1}}-\delta_{\mathrm{M1}})^2(\delta_{\mathrm{a1}}-\delta_{\mathrm{M1}})^2} \quad \text{S9}$$

$$\frac{\partial[\mathrm{M}^{2+}]_\mathrm{f}}{\partial\delta_{\mathrm{M1}}} = \frac{(\delta_{\mathrm{a1}}-\delta_{\mathrm{b1}})[(\delta_{\mathrm{L1}}-\delta_{\mathrm{M1}})[(\delta_{\mathrm{b1}}-\delta_{\mathrm{M1}})+(\delta_{\mathrm{a1}}-\delta_{\mathrm{M1}})]-(\delta_{\mathrm{b1}}-\delta_{\mathrm{M1}})(\delta_{\mathrm{a1}}-\delta_{\mathrm{M1}})]}{\mathrm{K}_{1,0}\gamma_2(\delta_{\mathrm{b1}}-\delta_{\mathrm{M1}})^2(\delta_{\mathrm{a1}}-\delta_{\mathrm{M1}})^2} \quad \text{S10}$$

$\Delta\delta_{b1}$, $\Delta\delta_{a1}$, $\Delta\delta_{L1}$ and $\Delta\delta_{M1}$ are taken as 0.0005 ppm in this work.[51] $\Delta K_{1,0}$ is provided in Section S4.

The 0.0005 ppm uncertainty in chemical shift determines the lower detection limit for the measurement of $[M^{2+}]_f$, below which $[M^{2+}]_f$ can be taken as zero within the uncertainty of the measurement. From Equation S1, the lower detection limit, $[M^{2+}]_{f,Lower}$ can be obtained as:

$$[\mathrm{M}^{2+}]_{\mathrm{f,Lower}} = -\frac{2(\delta_{\mathrm{L1}}-\delta_{\mathrm{M1}})\Delta\delta_{\mathrm{a1}}}{\mathrm{K}_{0,1}\gamma_{2,\mathrm{L}}[(\delta_{\mathrm{a1}}+\Delta\delta_{\mathrm{a1}}-\delta_{\mathrm{M1}})(\delta_{\mathrm{a1}}-\Delta\delta_{\mathrm{a1}}-\delta_{\mathrm{M1}})]} \quad \text{S11}$$

where $\gamma_{2,L}$ is obtained from I_0 as:[50]

$$\gamma_{2,\mathrm{L}} = 10^{-2.04\left[\frac{\sqrt{\mathrm{I}_0}}{1+\sqrt{\mathrm{I}_0}}-0.3\mathrm{I}_0\right]} \quad \text{S12}$$

For 50 mM NaCl with 1 mM glycolate and sulfoacetate (I_0 = 0.054 M), $[M^{2+}]_{f,Lower}$ is obtained as 0.4 and 0.8 mM for Ca and Mg, respectively. Below these limits, $[M^{2+}]_f$ is zero within the uncertainty of the measurement. Nevertheless, the spreadsheet accompanying this work calculates $[M^{2+}]_f$ explicitly along with associated uncertainties.

To determine whether a measurement of $[M^{2+}]_f$ is acceptable, the following filter is applied:

1. If the chemical shifts of glycolate and/or sulfoacetate are < (δ_a – 0.0005 ppm), the measurement is invalid and is set to -999.

2. If the chemical shifts of glycolate and/or sulfoacetate are within 0.0005 ppm of δ_a, but the modulus of the reported $[M^{2+}]_f > [M^{2+}]_{f,Lower}$, the measurement is invalid and is set to -999.

3. If the chemical shifts of glycolate and/or sulfoacetate are within 0.0005 ppm of δ_a, but the reported $[M^{2+}]_f$ is negative, $[M^{2+}]_f$ is set to 0. If the modulus of $[M^{2+}]_f > \Delta[M^{2+}]_f$, the measurement is invalid and is set to -999. Otherwise the $[M^{2+}]_f$ is reported as calculated using Equation S1.

4. If the chemical shifts of glycolate and sulfoacetate are > (δ_a + 0.0005 ppm), the validity of the measurement is judged by comparing the value of γ_2 obtained from Equation S2 with the value calculated from the reported $[M^{2+}]_f$ as $\gamma_2 - \Delta\gamma_2$ (Equation S3). The measurement is unacceptable if $\gamma_2 > (\gamma_2 - \Delta\gamma_2)\left(1 + {}^{h}/_{100}\right)$ or if $\gamma_2 < (\gamma_2 - \Delta\gamma_2)\left(1 - {}^{h}/_{100}\right)$, and $[M^{2+}]_f$ is set to -999. h is set to 60 in this work.

B is calculated from Equation 4. $[M^{2+}]_L$ is calculated from Equation 2 for chloride, acetate, glycolate and sulfoacetate using the values of K_0 provided in Section S4. If the measurement of $[M^{2+}]_f$ is invalid (see above), B is set to -999. If Equation 4 returns a negative value, B is set to 0.

To calculate the uncertainty in B, the maximum/minimum values of $[M^{2+}]_L$ are computed for each ligand in Table S3 using Equation 2:

$$[M^{2+}]_{L,max} = \frac{[L]_{total}\gamma_2^n K_0([M^{2+}]_f + \Delta[M^{2+}]_f)}{1 + \gamma_2^n K_0([M^{2+}]_f + \Delta[M^{2+}]_f)} \qquad S13$$

$$[M^{2+}]_{L,min} = \frac{[L]_{total}\gamma_2^n K_0([M^{2+}]_f - \Delta[M^{2+}]_f)}{1 + \gamma_2^n K_0([M^{2+}]_f - \Delta[M^{2+}]_f)} \qquad S14$$

The maximum B, B_{max}, is then estimated as:

$$B_{max} = 0.5[Ac]\left(1 + \frac{x}{100}\right)\sqrt{\frac{D_{Ac}}{D_M}}\exp\left[\frac{(Z-h)^2}{4t}\left(\frac{1}{D_{Ac}} - \frac{1}{D_M}\right)\right] - [M^{2+}]_{L,min} - \left([M^{2+}]_f - \Delta[M^{2+}]_f\right) \qquad S15$$

where x is the percentage error of [Ac] from integration of the ^{1}H NMR resonance of acetate. B_{min} is obtained as:

$$B_{min} = 0.5[Ac]\left(1 - \frac{x}{100}\right)\sqrt{\frac{D_{Ac}}{D_M}}\exp\left[\frac{(Z-h)^2}{4t}\left(\frac{1}{D_{Ac}} - \frac{1}{D_M}\right)\right] - [M^{2+}]_{L,max} - \left([M^{2+}]_f + \Delta[M^{2+}]_f\right) \qquad S16$$

The uncertainty in B, ΔB, is obtained as:

$$\Delta B = \frac{B_{max} - B_{min}}{2} \qquad S17$$

[Ac] was determined by integration of the ^{1}H resonances of acetate against either DMSO or tert-Butanol using Equation S18:

$$[Ac] = kA/R \qquad S18$$

where A and R denote the acetate and reference signals, respectively. To determine x (Equation S15, S16) and the conversion factor, k, the concentration of acetate determined by NMR was compared with the known concentration of acetate in the homogeneous titrations of NaCl, EDTA, PAA, PSS, PAA, PEI, and alginate. Averaging across all samples, k was determined as 2.92 and 3.83 for DMSO and tert-Butanol respectively, with a maximum deviation of 6% for all datasets, based on the slope of a plot of the measured versus known concentration of acetate. The random error in integration within a CSI dataset was determined as 5% by comparing the integrals of DMSO and tert-butanol in the NaCl and maleate samples of Figure 1b. x is thus estimated as 8%. The determination of [Ac] by integration against the resonances of DMSO or tert-Butanol, or by lineshape deconvolution were found to give equivalent results (Figure S-1).

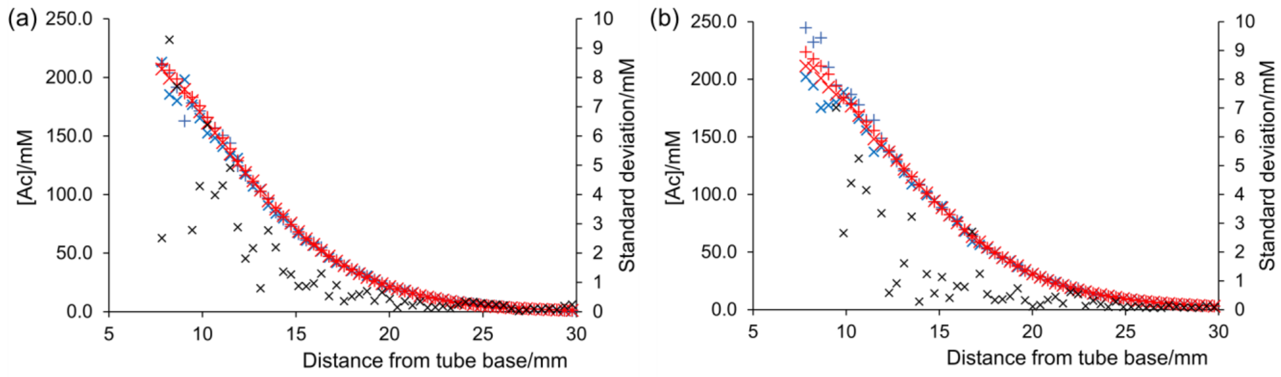

Figure S-1. Plot of [Ac] when Mg^{2+} acetate (a) and Ca^{2+} acetate (b) were diffused into 10 mM maleate (dataset of Figure 1b). [Ac] was determined by integration (blue) or lineshape deconvolution (red) with DMSO (St Andrew's cross) or tert-Butanol (vertical cross) as references. Standard deviation across all four methods is plotted as small black cross.

S2. Determination of pH from ^{1}H chemical shift of 2-methylimidazole

The pH of the solution at each position along the M^{2+} gradient is determined from the observed ^{1}H chemical shift, δ_{obs}, of 2-methylimidazole using Equation S19:

$$\mathrm{pH} = \mathrm{pK_{a,0}} + \frac{0.51\sqrt{I}}{1+\sqrt{I}} - 0.1I + \log_{10}\left(\frac{\delta_H - \delta_{obs}}{\delta_{obs} - \delta_L}\right) \qquad \text{S19}$$

where I is the ionic strength of the solution. δ_H and δ_L are the chemical shifts of the fully protonated and deprotonated forms, respectively. The pK_a at I = 0, p$K_{a,0}$, is taken as 7.96 from our previous work.[51] For calculation of pH in this work using Equation S19, we use the ionic strength of the solution prior to diffusion of M^{2+} acetate (I_0, Section S-1) rather than explicitly calculate the ionic strength at each point along our gradient. According to Equation S19, this approximation introduces an error of <0.2 units in the measured pH. It has been demonstrated that δ_H and δ_L of imidazole are not significantly affected by the presence of Ca^{2+} or Mg^{2+}.[52] δ_H and δ_L for 2-methylimidazole are provided in Table S-1. pH measured in CSI titrations of NaCl, maleate, EDTA, PAA, PSS, PEI (Figures 1 and 2) are provided on Figure S-2b,c)

**δ_H/ppm (DSS)**	**δ_L/ppm (DSS)**	**δ_H/ppm (DMSO)**	**δ_L/ppm (DMSO)**
2.6044	2.3450	2.6080	2.3486
7.2673	6.9586	7.2709	6.9622

Table S-1. δ_H and δ_L of 2-methylimidazole referenced to DSS (0 ppm) and DMSO (2.72 ppm). Values are provided for the methyl (upper) and aromatic (lower) resonances. Chemical shifts relative to DMSO have been obtained by adding 0.0036 ppm to the values in DSS, following our previous work.[51] pH values determined using the two chemical shift references agree to < 0.04 units (Figure S-2a).

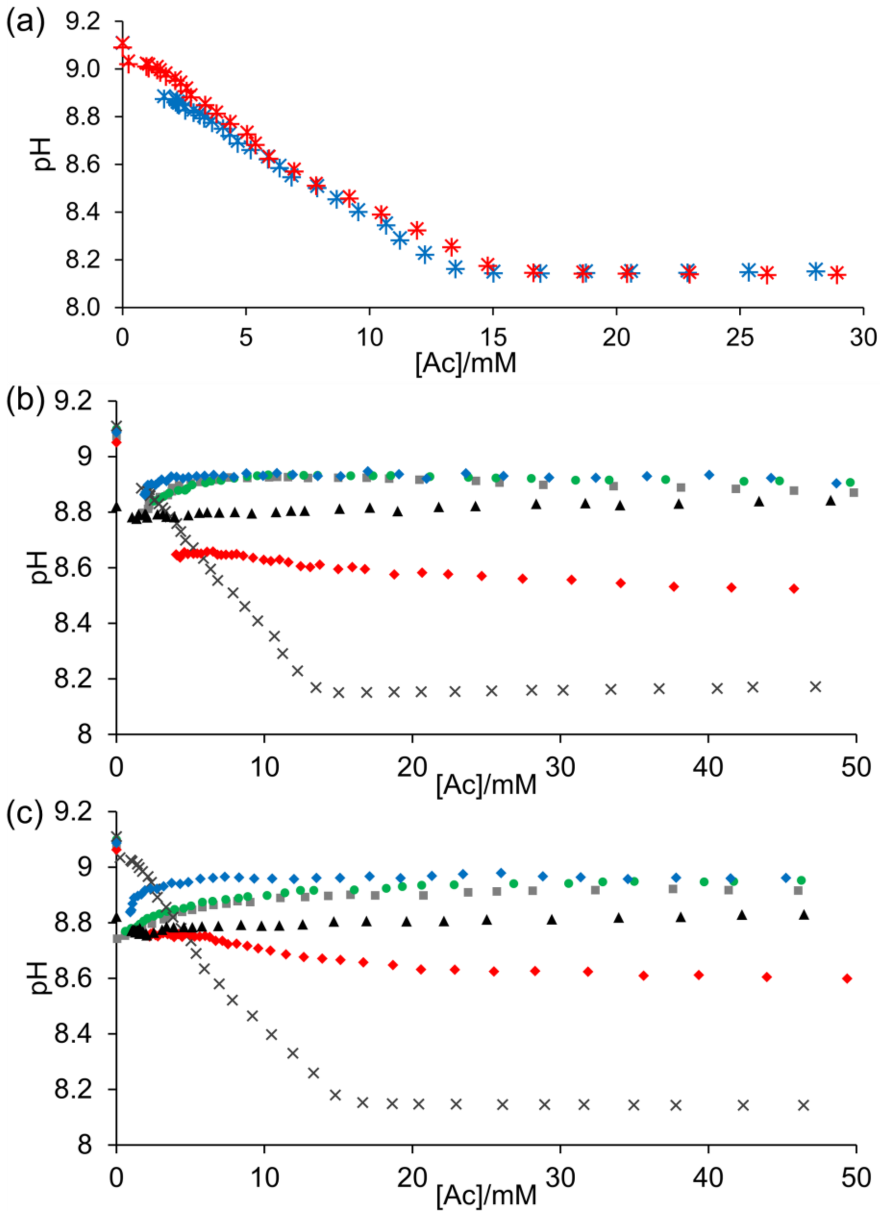

Figure S-2. (a) Plot of pH calculated from chemical shift of 2-methylimidazole referenced to DSS (vertical cross) and DMSO (St Andrew's cross) in CSI titration of 5 mM EDTA with calcium (blue) and magnesium (red). Datasets are the same as on Figure 1a. (b-c): Plots of pH versus [Ac] in CSI titration of 5 mM EDTA (grey cross), 50 mM NaCl (grey square), 10 mM maleate (green circle), PAA (red diamond), PEI (black triangle) and PSS (blue diamond). (b) Ca, (c), Mg. Datasets are the same as on Figures 1 and 2. We note that our method to measure $[M^{2+}]_f$ functions between pH 7 and pH 12.[50]

S3. CSI analysis of M^{2+} gradients at different times since preparation

The time at which a CSI experiment was run since preparation of the sample was rounded to the nearest half hour. This time was used in the calculation of N (Equation 3).

Sample	Time since preparation/hour
PAA (Mg)	8
PAA (Ca)	8.5
PSS (Mg)	9
PSS (Ca)	8.5
PEI (Mg)	7
PEI (Ca)	6.5
4 mg/mL sodium alginate (Mg)	7.5
4 mg/mL sodium alginate (Ca)	7
2 mg/mL sodium alginate (Mg)	8.5
2 mg/mL sodium alginate (Ca)	8.5
Citrate-CNC (Mg)	8.5
Citrate-CNC (Ca)	8
EDTA (Mg)	8
EDTA (Ca)	7.5
Maleate (Mg)	9
Maleate (Ca)	8.5
NaCl (Mg)	5.5
NaCl (Ca)	5

Table S-2. Time elapsed between preparation and analysis of CSI samples presented in the main paper.

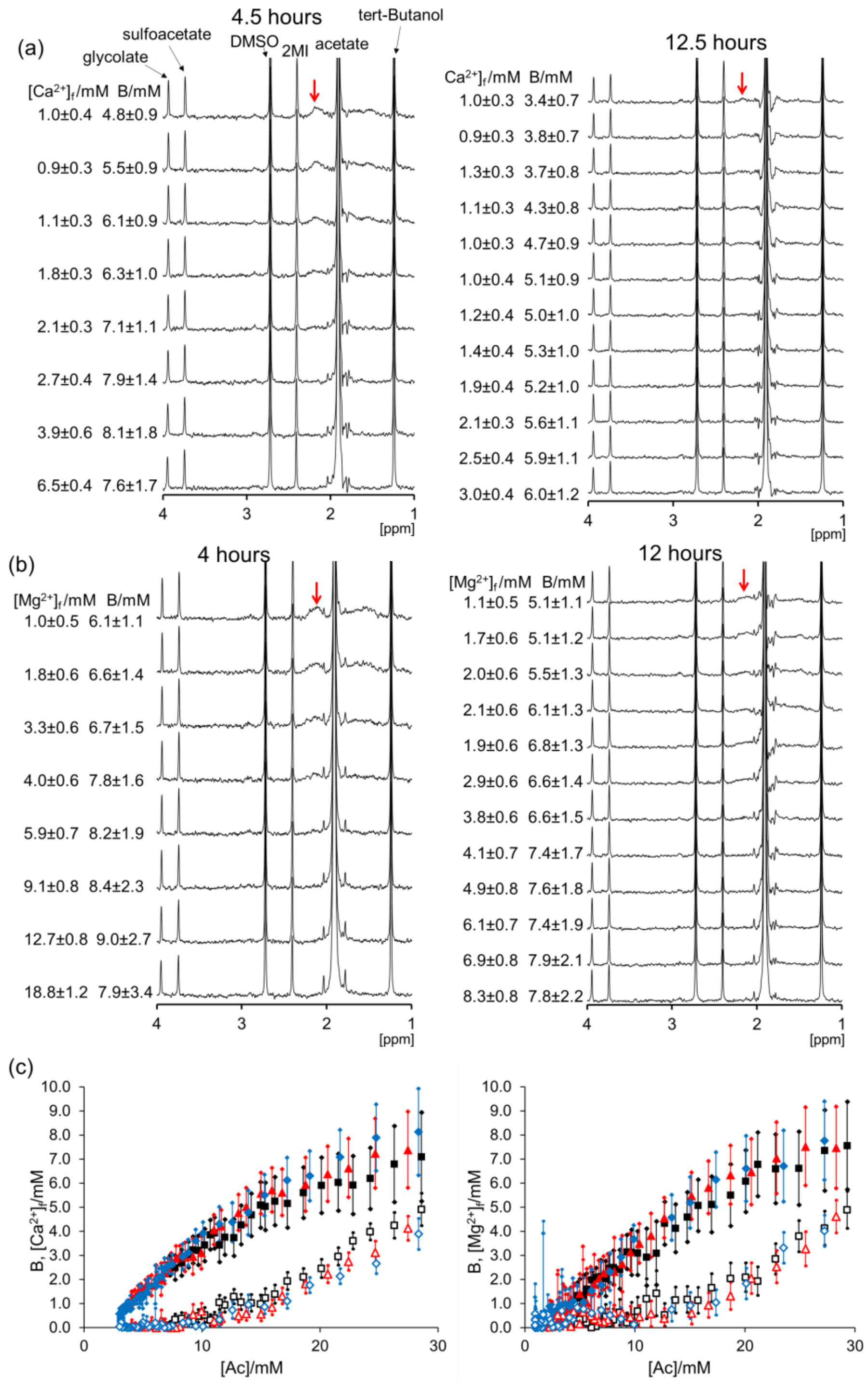

Figure S-3. PAA: ^{1}H spectra extracted from PAA CSI datasets measured on sample of Figure 2a at time indicated since preparation. (a) Ca, (b) Mg. (c) Plot of B (solid symbols) and $[M^{2+}]_f$ (open) when calcium acetate (left) or magnesium acetate (right) is diffused into solution of PAA. The sample was analysed at the following times since preparation: Ca: 4.5 hours (blue diamond), 8.5 hours (red triangle), 12.5 hours (black square). Mg: 4 hours (blue diamond), 8 hours (red triangle), 12 hours (black square).

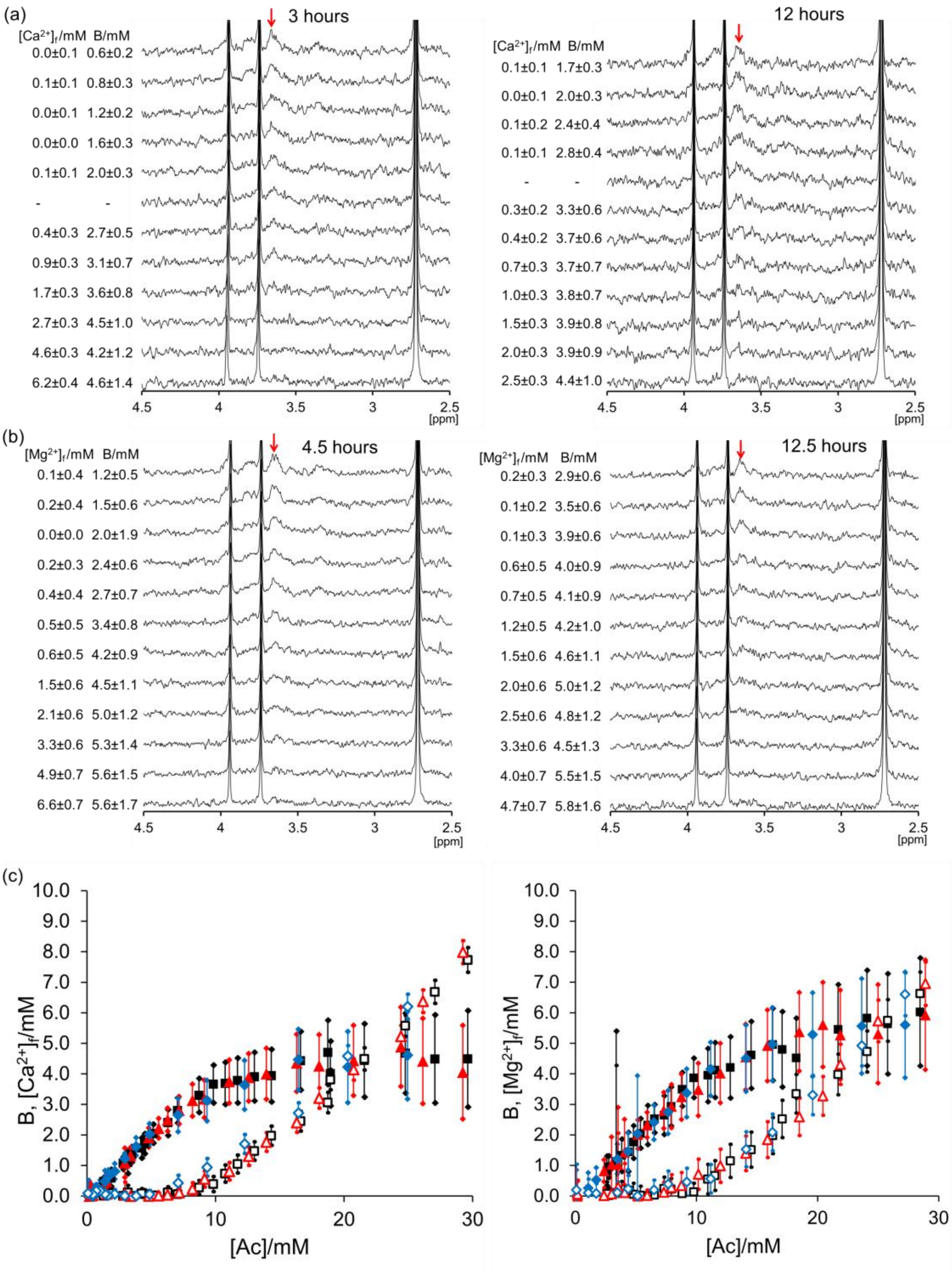

Figure S-4. 2 wt% citrate-CNC: ^{1}H spectra extracted from citrate-functionalised CNC CSI datasets measured on sample of Figure 4c at time indicated since preparation. (a) Ca, (b) Mg. (c) Plot of B (solid symbols) and $[M^{2+}]_f$ (open) when calcium acetate (left) or magnesium acetate (right) was diffused into solution of citrate-CNC. The sample was analysed at the following times since preparation: Ca: 3 hours (blue diamond), 8 hours (red triangle), 12 hours (black square). Mg: 4.5 hours (blue diamond), 8.5 hours (red triangle), 12.5 hours (black square).

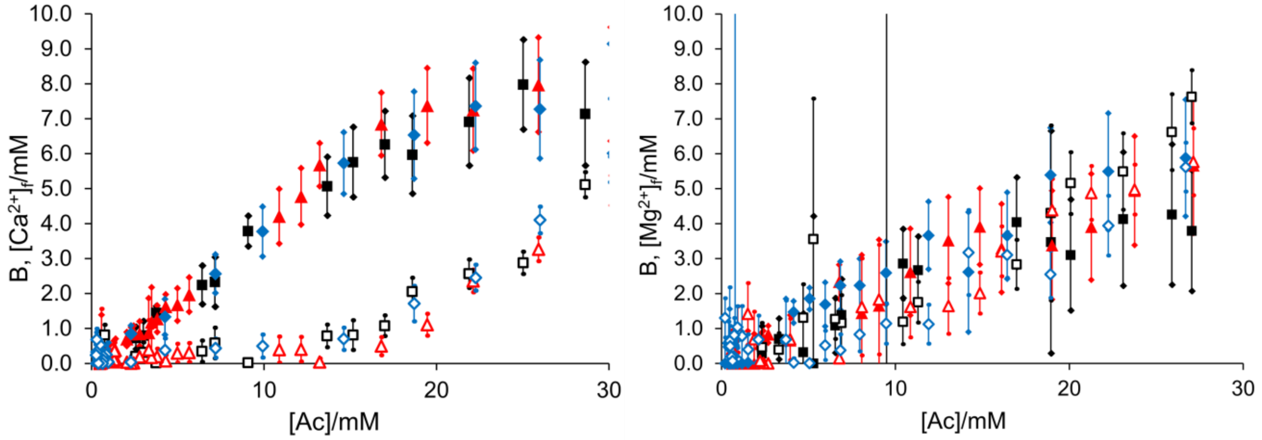

Figure S-5. Plot of B (solid symbols) and $[M^{2+}]_f$ (open symbols) when calcium acetate (left) or magnesium acetate (right) was diffused into 4 mg/mL sodium alginate. The sample was analysed at the following times since preparation: Ca: 4.5 hours (blue diamond), 7 hours (red triangle), 10 hours (black square). Mg: 4.5 hours (blue diamond), 7.5 hours (red triangle), 10.5 hours (black square).

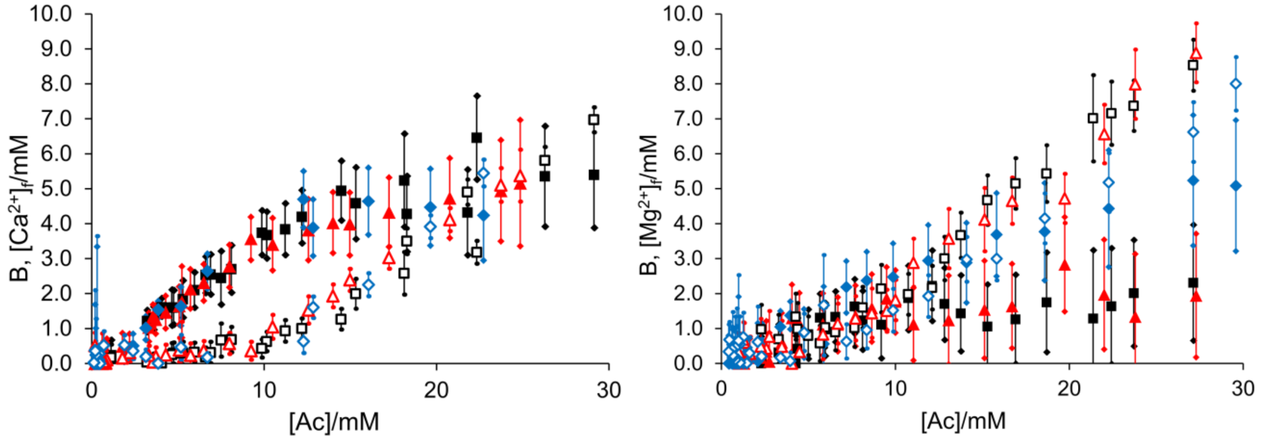

Figure S-6. Plot of B (solid symbols) and $[M^{2+}]_f$ (open symbols) when calcium acetate (left) or magnesium acetate (right) was diffused into 2 mg/mL sodium alginate. The sample was analysed at the following times since preparation: Ca: 4.5 hours (blue diamond), 8.5 hours (red triangle), 12.5 hours (black square). Mg: 5 hours (blue diamond), 8.5 hours (red triangle), 11.5 hours (black square).

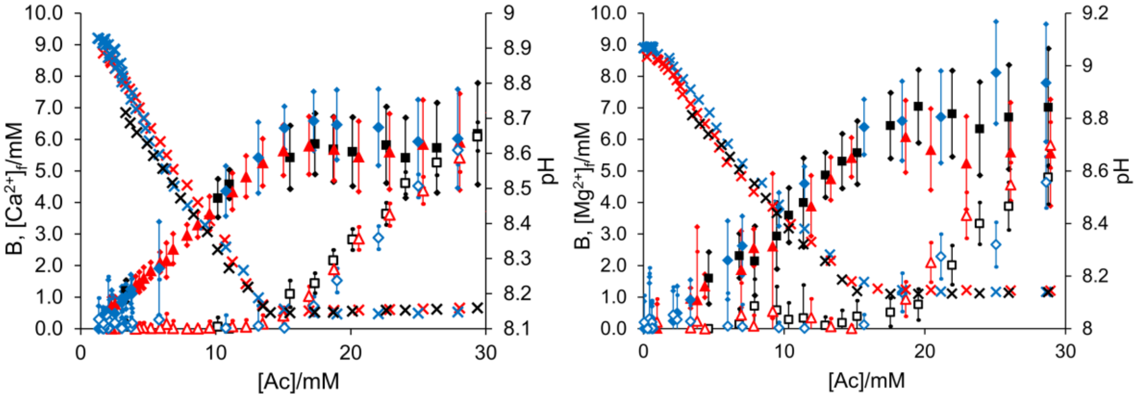

Figure S-7. Plot of B (solid symbols) and $[M^{2+}]_f$ (open symbols) when calcium acetate (left) or magnesium acetate (right) was diffused into 5 mM EDTA. pH (cross). The sample was analysed at the following times since preparation: Ca: 4.5 hours (blue diamond), 7.5 hours (red triangle), 13.5 hours (black square). Mg: 5 hours (blue diamond), 8 hours (red triangle), 14 hours (black square).

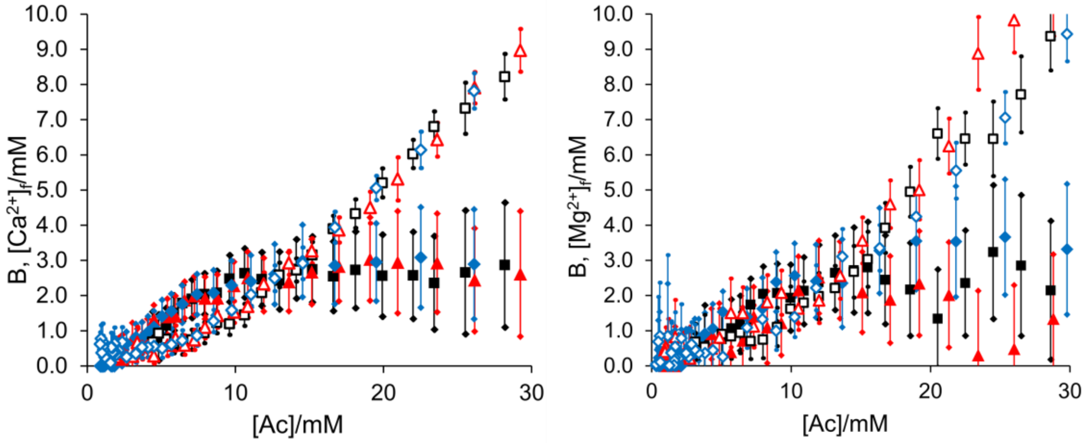

Figure S-8. Plot of B (solid symbols) and $[M^{2+}]_f$ (open symbols) when calcium acetate (left) or magnesium acetate (right) was diffused into PSS. The sample was analysed at the following times since preparation: Ca: 4.5 hours (blue diamond), 8.5 hours (red triangle), 12.5 hours (black square). Mg: 4.5 hours (blue diamond), 9 hours (red triangle), 12.5 hours (black square).

S4. Ligand binding constants and limiting chemical shifts

**Ligand**	**δ_{Ca}/ppm**	**δ_{Mg}/ppm**	**δ_L/ppm**	**$K_{0,Ca}$/M^{-1}**	**$K_{0,Mg}$/M^{-1}**
Glycolate	4.0977	4.0471	3.9353	34.7±0.06	25.4±0.6
Sulfoacetate	3.8038	3.7812	3.7332	144.4±0.7	117.4±2.4
Acetate[a]	-	-	-	5.89	1.10
Chloride	-	-	-	2.63[b]	0[c]

Table S-3. Chemical shifts of free (δ_L) and fully complexed (δ_{Ca}, δ_{Mg}) glycolate and sulfoacetate ligands and binding constants used in this work, taken from Reference 50. Chemical shifts are reported relative to DMSO at 2.72 ppm in 100% H_2O. Shifts relative to DSS and methanol, and in 90% H_2O/10% D_2O are provided in our previous work. a: Reference 50. b: Reference 53. c: as discussed in our previous work, glycolate and sulfoacetate are unable to distinguish between free Mg^{2+} ions and Mg-Cl ion pairs and the binding constant is set to zero in the present work for simplicity.[50]

S5. Salt effect on diffusion of M^{2+} and acetate, derivation of Equation 3

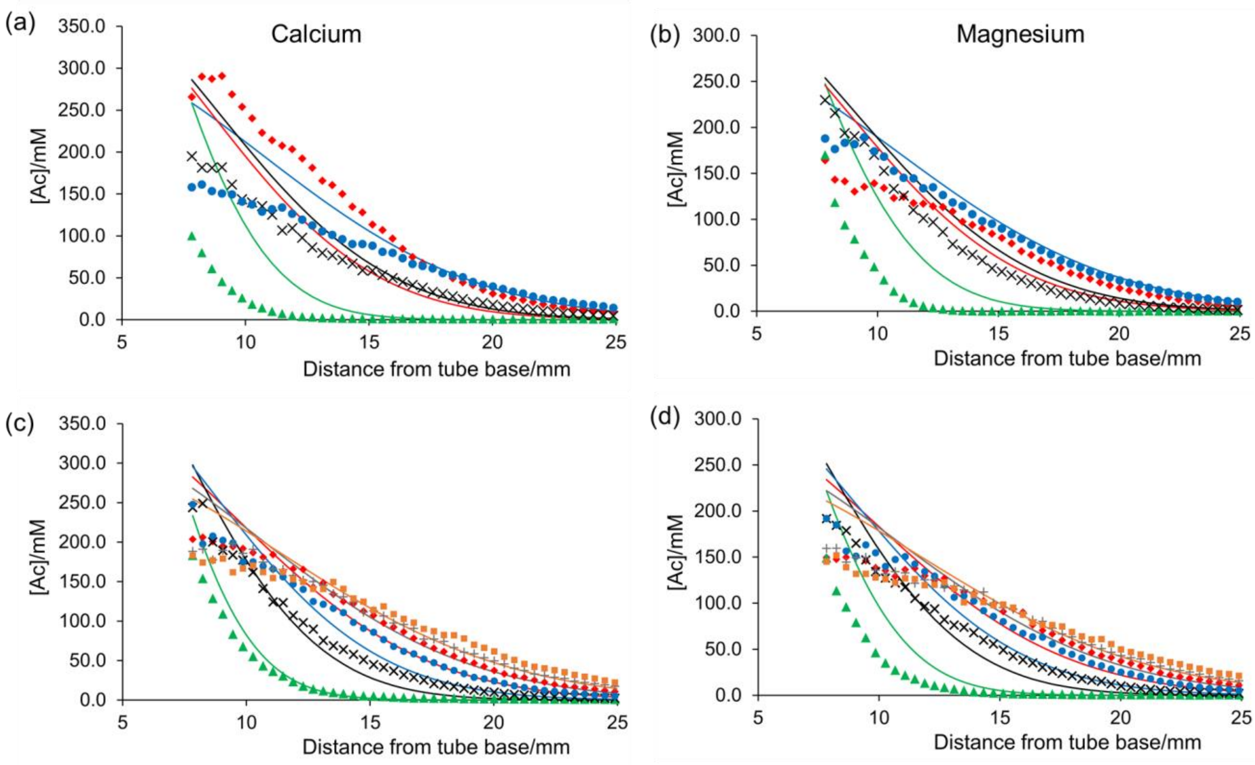

Figure S-9. Concentration of acetate versus vertical position from base of tube (Z, Equation S20) when calcium (left a, c) or magnesium (right b, d) acetate is diffused into a solution containing 50 mM NaCl. Sample also contained glycolate (1 mM), sulfoacetate (1 mM), DMSO (0.01 vol%), DSS (0.2 mM), 2MI (1 mM) and tert-Butanol (0.01 vol%). (a) m = 4.5 mg, t = 2 (green triangle), 4 (black cross), 6 (blue circle), 8 (red diamond), 10 (grey cross) and 12 hours (orange square) since sample preparation. (b) m = 4.5 mg, t = 2.5 (green triangle), 4.5 (black cross), 6.5 (blue circle), 8.5 (red diamond), 10.5 (grey cross) and 12.5 (orange square) hours since preparation. (c) m = 4.4 mg, t = 2.5 (green triangle), 6.5 (black cross), 10.5 (blue circle) hours since preparation. m = 4.2 mg, t = 6 hours (red diamond). (d) m = 4.2 mg, t = 3 (green triangle), 7 (black cross), 11 (blue circle) hours since preparation. m = 4.5 mg, t = 6.5 hours (red diamond). The lines are the acetate concentration predicted using Equation S20, with D_{Ac} = 1 x 10^{-9} m^2s^{-1}.

To determine D_{Ac} and D_M (Equation 3), samples were prepared containing 50 mM NaCl, 1 mM 2MI, 1 mM glycolate and sulfoacetate, 0.2 mM DSS, 0.01 vol% DMSO and 0.01 vol% tert-butanol. Three experiments were performed for Ca^{2+} and Mg^{2+} to assess the reproducibility of the concentration gradients (Figure S-9, above). [Ac] can be assumed to follow a Gaussian profile (Equation S20):

$$[\mathrm{Ac}] = \frac{2\mathrm{m}}{\pi \mathrm{r}^2 \mathrm{M_r} \sqrt{\pi \mathrm{D_{Ac}} \mathrm{t}}} \exp\left[\frac{-(\mathrm{Z}-\mathrm{h})^2}{4\mathrm{D_{Ac}}\mathrm{t}}\right] \qquad \text{S20}$$

where m is the mass of M^{2+} acetate salt weighed into the tube, r the tube radius (2.1 mm), M_r the molecular weight of the salt (180 g/mol for calcium acetate hydrate, 214.5 g/mol for magnesium acetate tetrahydrate), D_{Ac} the diffusion coefficient of acetate, t the time since sample preparation, Z the vertical position from the absolute bottom of the NMR tube and h the thickness of the acetate when weighed out (2 mm). D_{Ac} of 1 x 10^{-9} m^2s^{-1} was chosen, based on visual inspection of the data (Figure S-9). This value is in good agreement with results presented elsewhere for diffusion of acetate.[54] The poor agreement with Equation S20 at t < 3 hours is attributable to unavoidable upward mixing of the acetate salt during preparation of the sample.

To estimate D_M, N was calculated for the datasets on Figure S-9 using Equation 1 (B = 0). Fits were then generated using Equation 3 and the value of D_M chosen to obtain the best visual fit to the data. Plots are provided on Figure S-10:

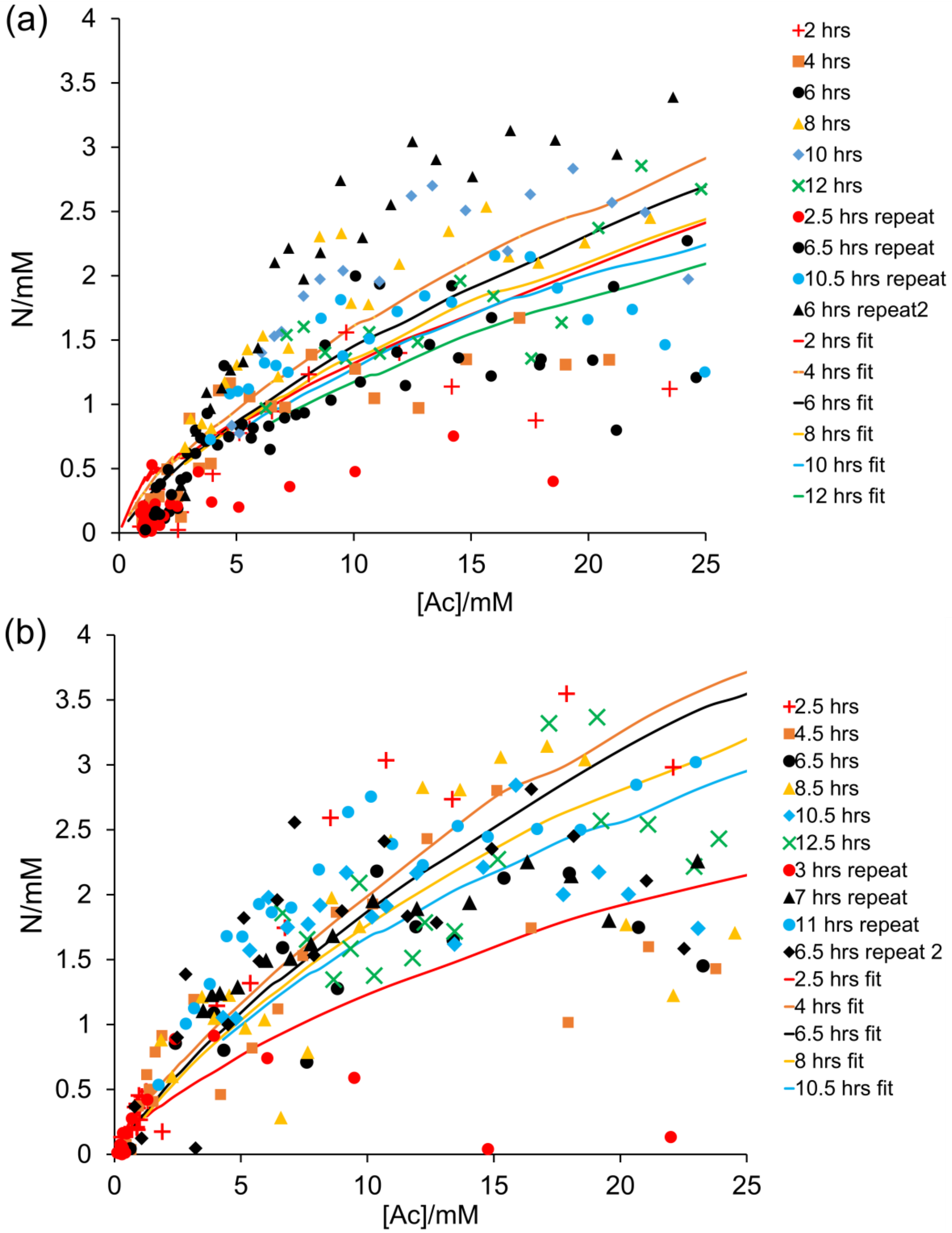

Figure S-10. Plot of N obtained using Equation 1 (data points) and fit to Equation 3 (solid lines) for diffusion of calcium (a) and magnesium (b) acetate into 50 mM NaCl. The datasets are the same as used for Figure S-9. Time elapsed since preparation is indicated in the legend. Masses of M^{2+} acetate are as follows: calcium: m = 4.5 mg, repeat m = 4.4 mg, second repeat m = 4.2 mg. $D_M = 9.3 \times 10^{-10}$ m^2s^{-1}. Magnesium: m = 4.5 mg, repeat m = 4.2 mg, second repeat m = 4.5 mg. $D_M = 9.0 \times 10^{-10}$ m^2s^{-1}.

N (Equation 3) is defined as the discrepancy between [Ac] and the total concentration of M^{2+} at a particular point in the sample, $[M^{2+}]_{total}$, assuming B = 0:

$$N = 0.5[Ac] - [M^{2+}]_{total} \quad \text{S21}$$

Assuming acetate and M^{2+} follow independent Gaussian profiles (Equation S20), we obtain the ratio of the two at any point from Equation S22:

$$\frac{[M^{2+}]_{total}}{0.5[Ac]} = \sqrt{\frac{D_{Ac}}{D_M}} \exp\left[\frac{(Z-h)^2}{4t}\left(\frac{1}{D_{Ac}} - \frac{1}{D_M}\right)\right] \quad \text{S22}$$

Equation 3 is obtained by combining Equations S21 and S22.

S6. ¹H spectra of PSS, PEI and EDTA

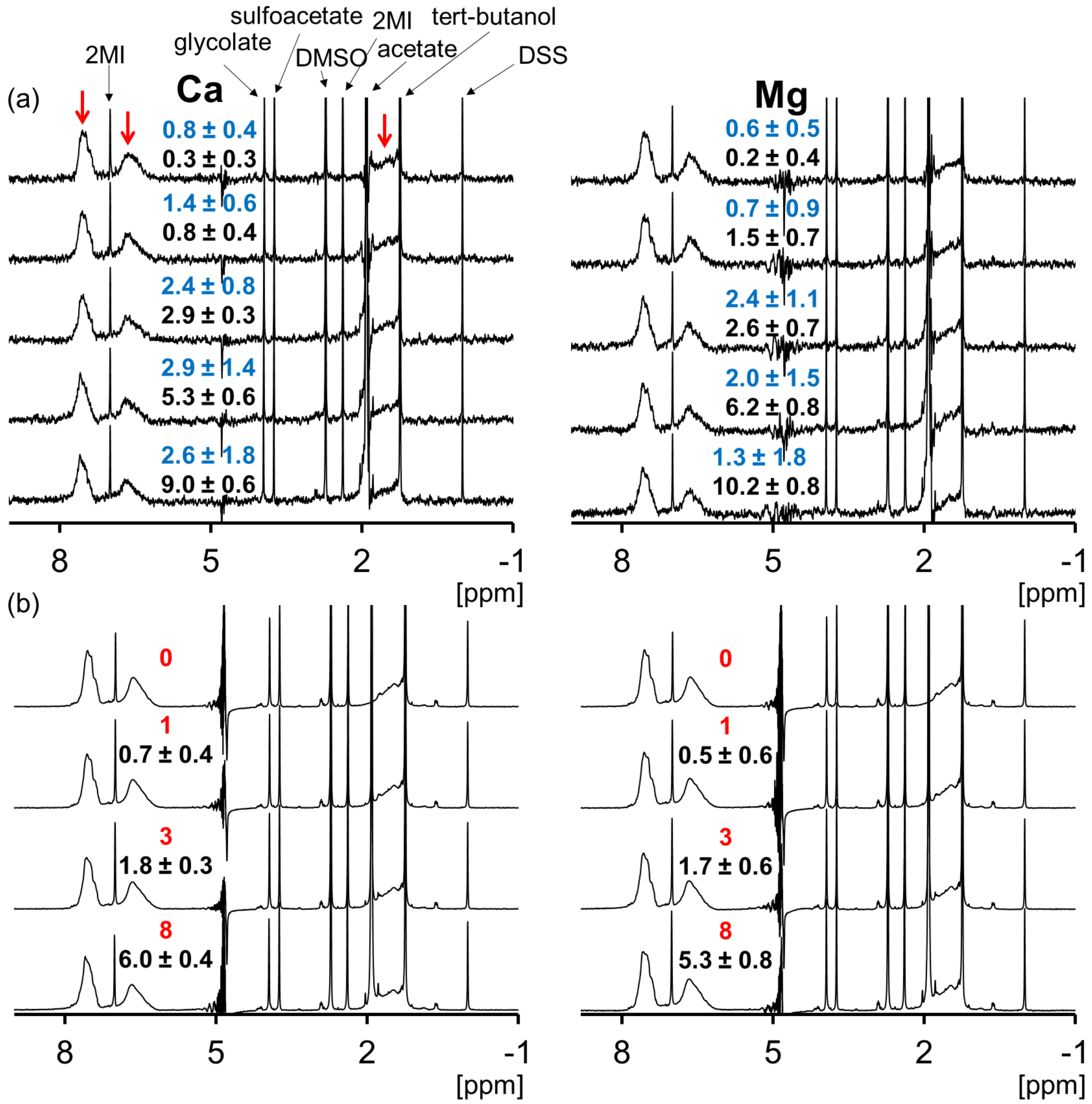

Figure S-11. (a) ¹H spectra of PSS extracted from CSI dataset of Figure 2b. ¹H resonances of PSS are indicated with red arrows. Ca^{2+} (left) and Mg^{2+} (right). B/mM (blue) and $[M^{2+}]_f$/mM (black). (b) ¹H spectra of homogenous samples of PSS and M^{2+}. $[M^{2+}]_{tot}$/mM (red) and $[M^{2+}]_f$/mM (black).

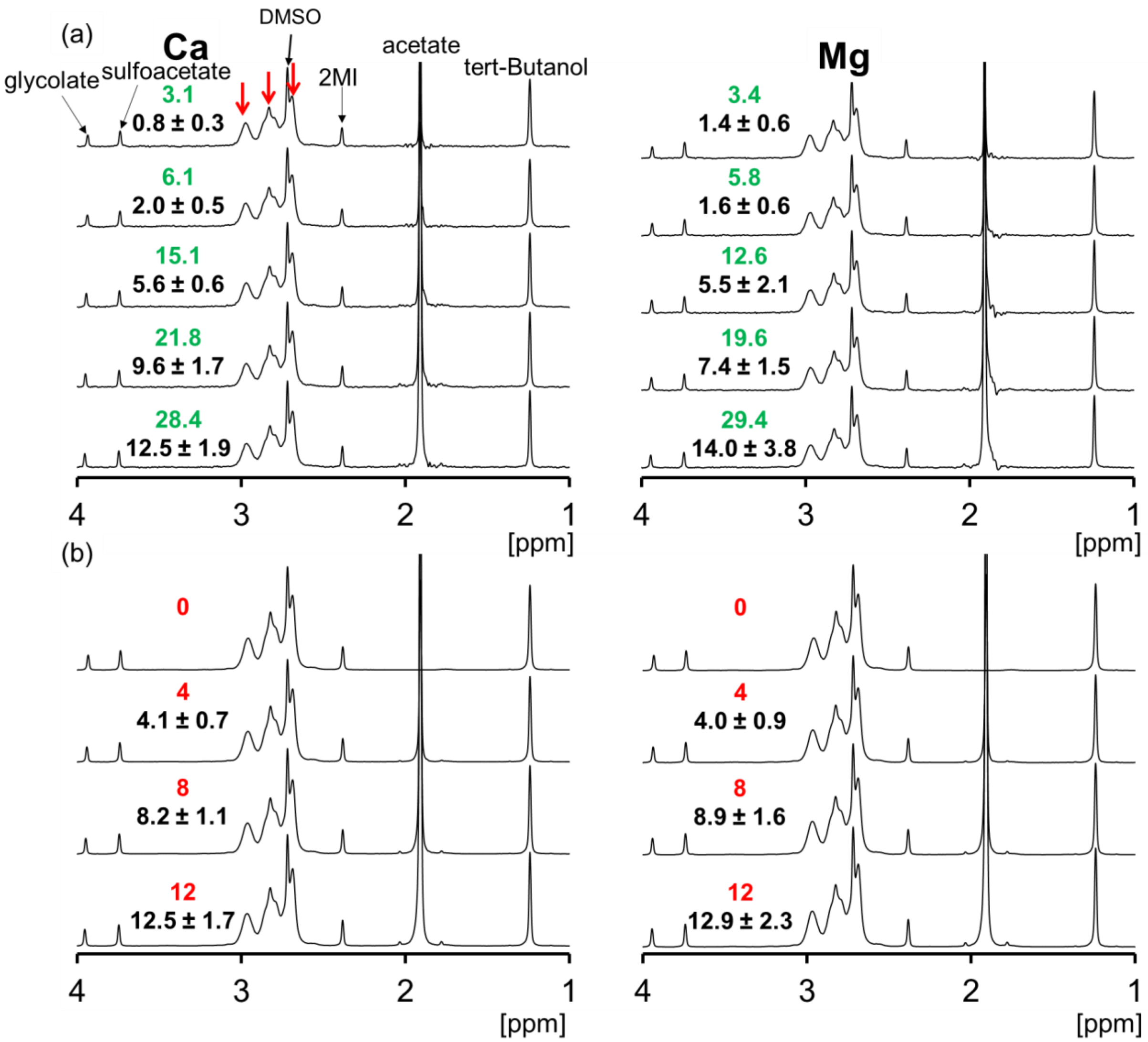

Figure S-12. (a) ^{1}H spectra of PEI extracted from CSI dataset of Figure 2c. ^{1}H resonances of PEI are indicated with red arrows. Ca^{2+} (left) and Mg^{2+} (right). [Ac]/mM (green) and $[M^{2+}]_f$/mM (black). (b) ^{1}H spectra of homogenous samples of PEI and M^{2+}. $[M^{2+}]_{tot}$/mM (red) and $[M^{2+}]_f$/mM (black).

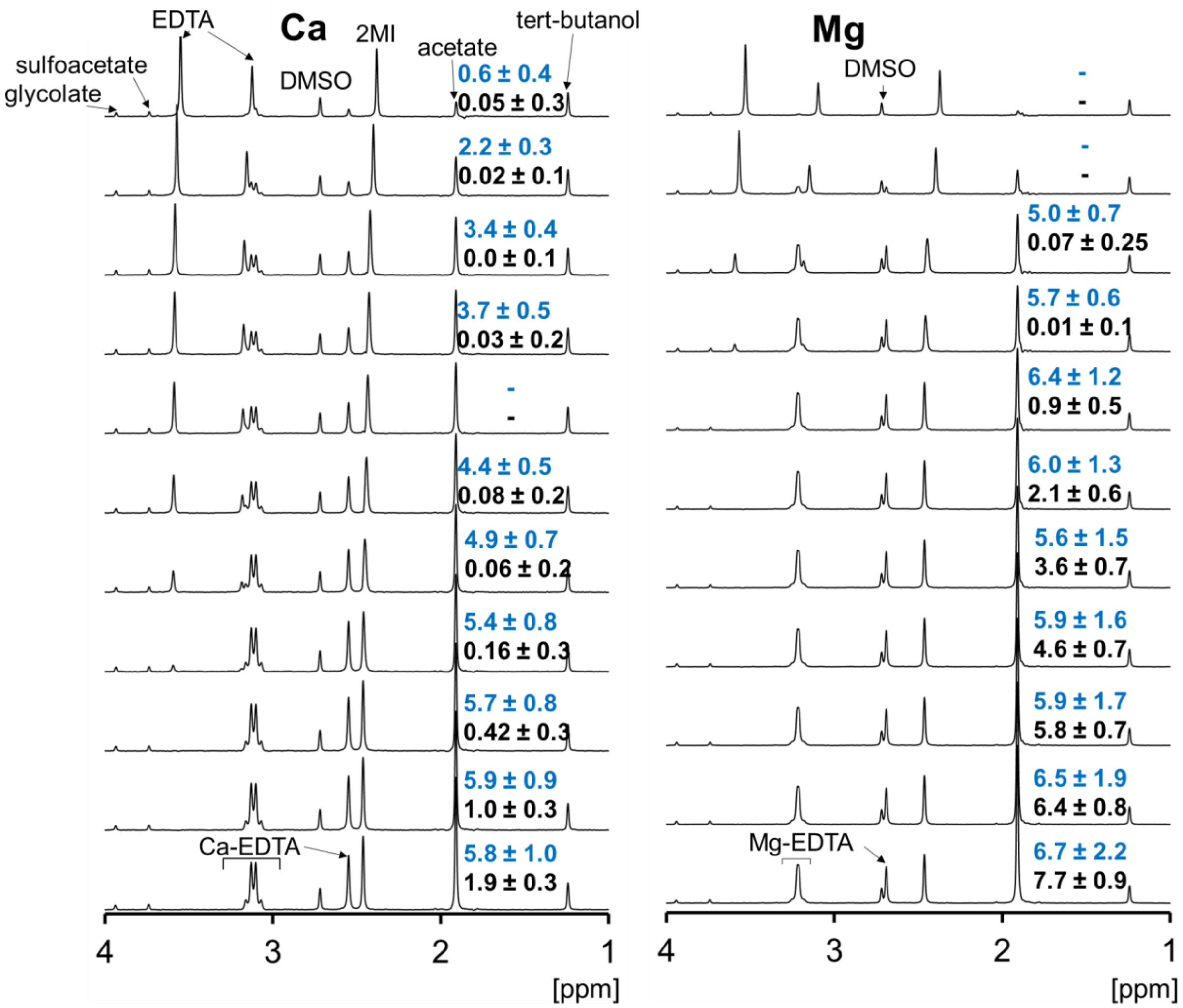

Figure S-13. ^{1}H spectra of EDTA extracted from CSI datasets of Figure 1a. Ca^{2+} (left) and Mg^{2+} (right). B/mM (blue) and $[M^{2+}]_f$/mM (black). Assignments of ^{1}H resonances of EDTA and M^{2+} complexes are made according to Monakhova *et al.*[55] $[M^{2+}]_f$ remains zero within experimental uncertainty until all of the EDTA has been complexed and the ^{1}H resonances of free EDTA have vanished.

S7. Interpretation of plots of B and $[M^{2+}]_f$ versus [Ac]

The interaction of M^{2+} with the polymer reduces D_M below the value measured in 50 mM NaCl, $D_{M,NaCl}$ (Section S-5). Assuming D_M is constant throughout the sample, the total concentration of M^{2+}, $[M^{2+}]_{total}$, at any vertical position along the sample is given by Equation S23:

$$[M^{2+}]_{total} = \frac{m}{\pi r^2 M_r \sqrt{\pi D_M t}} \exp\left[\frac{-(Z-h)^2}{4D_M t}\right] \qquad S23$$

(see Equation S20). Setting $[M^{2+}]_b = 0$, $[M^{2+}]_L = 0$, so that $[M^{2+}]_f = [M^{2+}]_{total}$, we may calculate B as:

$$B = 0.5[Ac] - N - [M^{2+}]_{total} \qquad S24$$

where [Ac] is calculated using Equation S20, and N using Equation 3. In real polymer systems, B thus contains a contribution from the reduction in D_M, as well as M^{2+} associated with the polymer. Therefore $B \geq [M^{2+}]_b$, provided $N \geq 0$. Plots of $[M^{2+}]_{total}$ and B are provided on Figure S-14 for different values of D_M.

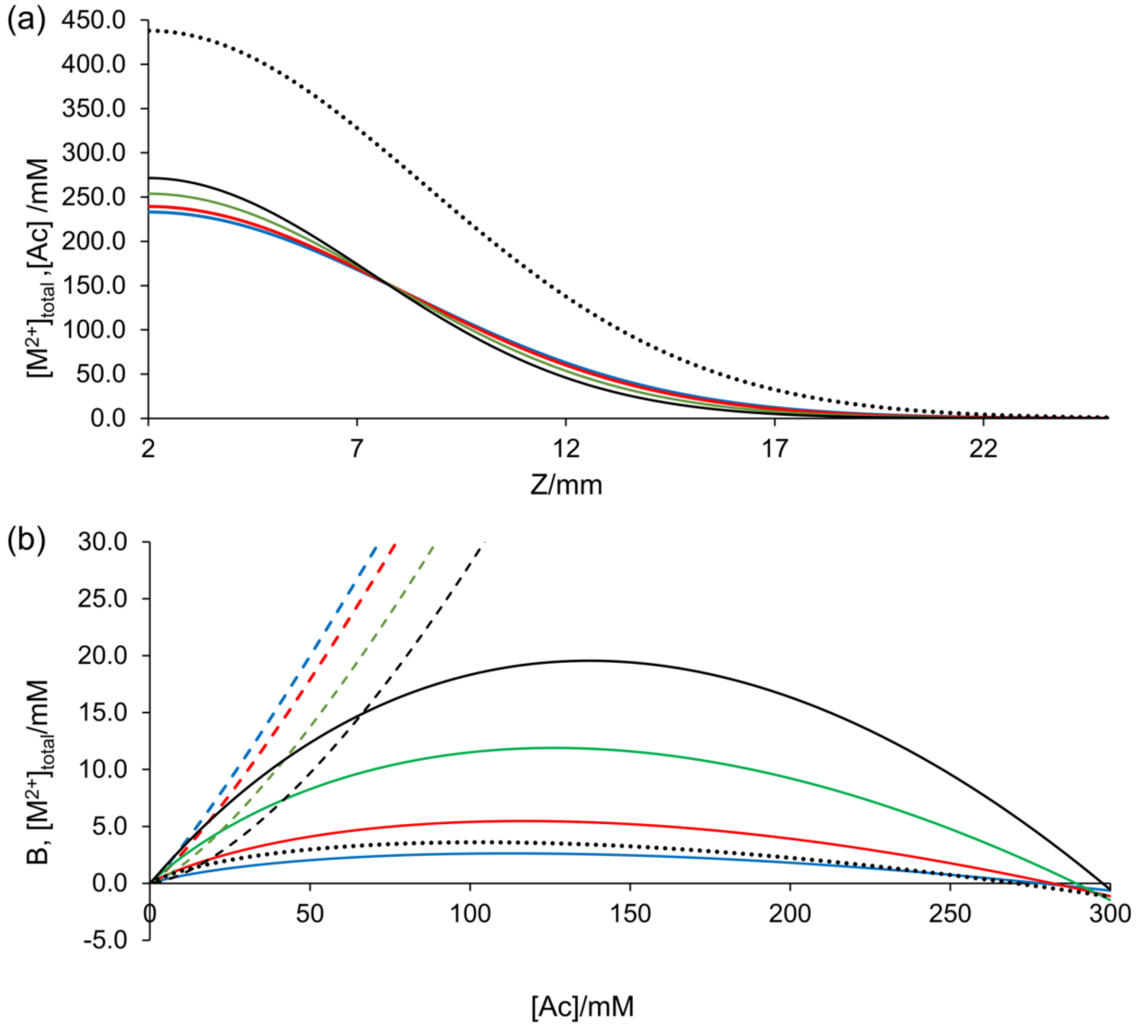

Figure S-14. (a) Plot of $[M^{2+}]_{total}$ versus Z at $D_M/D_{M,NaCl}$ of 0.95 (blue), 0.90 (red) 0.80 (green) and 0.70 (black) calculated using Equation S23. [Ac] (black, dotted line), Equation S20. (b) Plot of B (solid line) and $[M^{2+}]_{total}$ (dashed) at $D_M/D_{M,NaCl}$ of 0.95 (blue), 0.90 (red) 0.80 (green) and 0.70 (black) calculated using Equation S24. N calculated using Equation 3 (dotted line). In these calculations (for Ca^{2+} as plotted): h = 2 mm, r = 2.1 mm, m = 4.5 mg, M_r = 180 gmol^{-1} (calcium acetate hydrate), t = 6 hours, $D_{Ac} = 1 \times 10^{-9}$ m^{2}s^{-1}, $D_{M,NaCl} = 9.3 \times 10^{-10}$ m^{2}s^{-1}. $[M^{2+}]_b = 0$, $[M^{2+}]_L$ = 0.

Exchange of bound and free M^{2+} ions on the polymer will lower D_M. Assuming that the diffusion coefficient of M^{2+} in the bound state is zero, we can write:

$$\frac{D_M}{D_{M,NaCl}} = \frac{[M^{2+}]_f}{[M^{2+}]_{total}} \quad S25$$

$[M^{2+}]_f$ can be calculated from the total concentration of binding sites, $[S]_{tot}$, polymer binding site constant, K, and $[M^{2+}]_{total}$ using Equation S26:[50]

$$[M^{2+}]_f = [M^{2+}]_{total} - \frac{\left([M^{2+}]_{total} + [S]_{tot} + \frac{1}{K}\right) - \sqrt{\left([M^{2+}]_{total} + [S]_{tot} + \frac{1}{K}\right)^2 - 4[M^{2+}]_{total}[S]_{tot}}}{2} \quad S26$$

$[M^{2+}]_b$ is calculated in this section as $[M^{2+}]_{total} – [M^{2+}]_f$. Plots of $D_M/D_{M,NaCl}$, $[M^{2+}]_b$ and $[M^{2+}]_f$ are provided on Figure S-15 below. The exchange between free and bound M^{2+} means that $D_M < D_{M,NaCl}$, even when the binding sites are saturated.

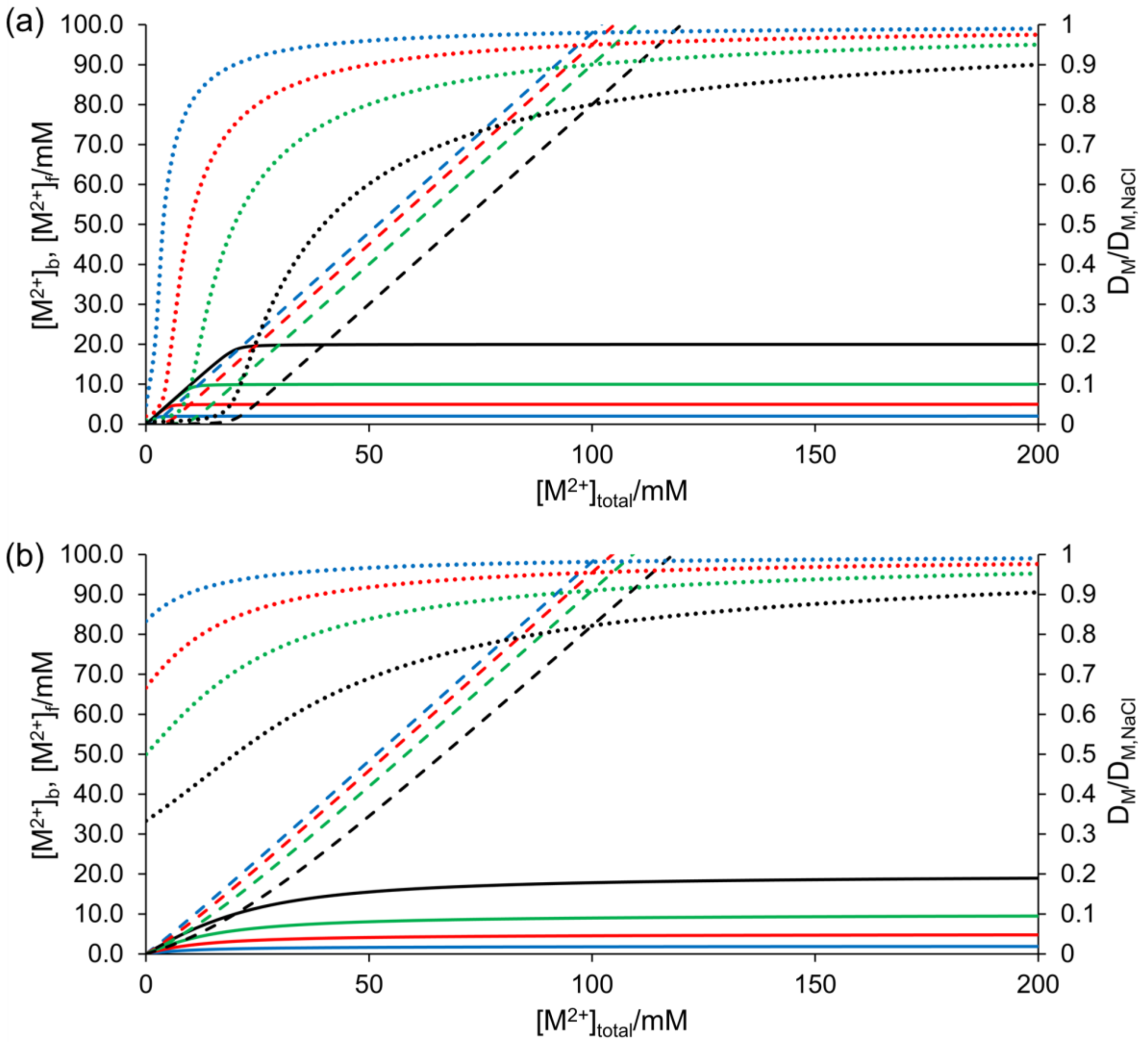

Figure S-15. Plots of $[M^{2+}]_b$ (solid line), $[M^{2+}]_f$ (dashed) and $D_M/D_{M,NaCl}$ (dotted) at $[S]_{tot}$ = 2 mM (blue), 5 mM (red), 10 mM (green) and 20 mM (black). K = 10^4 M^{-1} (a) and 10^2 M^{-1} (b). In these calculations (for Ca^{2+} as plotted): h = 2 mm, r = 2.1 mm, m = 4.5 mg, M_r = 180 $gmol^{-1}$ (calcium acetate hydrate), t = 6 hours, D_{Ac} = 1 x 10^{-9} m^2s^{-1}, $D_{M,NaCl}$ = 9.3 x 10^{-10} m^2s^{-1}.

We can now simulate the plots of B and $[M^{2+}]_f$ versus [Ac] obtained in a CSI experiment. $[M^{2+}]_{total}$ is obtained from Equation S23, B from Equation S24 and $[M^{2+}]_f$ from Equation S26. Plots are provided for various values of K on Figure S-16, fixing $D_M/D_{M,NaCl}$ as 0.9. With strong binders ($K \geq 10^3$ M^{-1}), B has exceeded $[S]_{tot}$ when $[M^{2+}]_f$ rises above 1 mM, as is observed for PAA (Figure 2a).

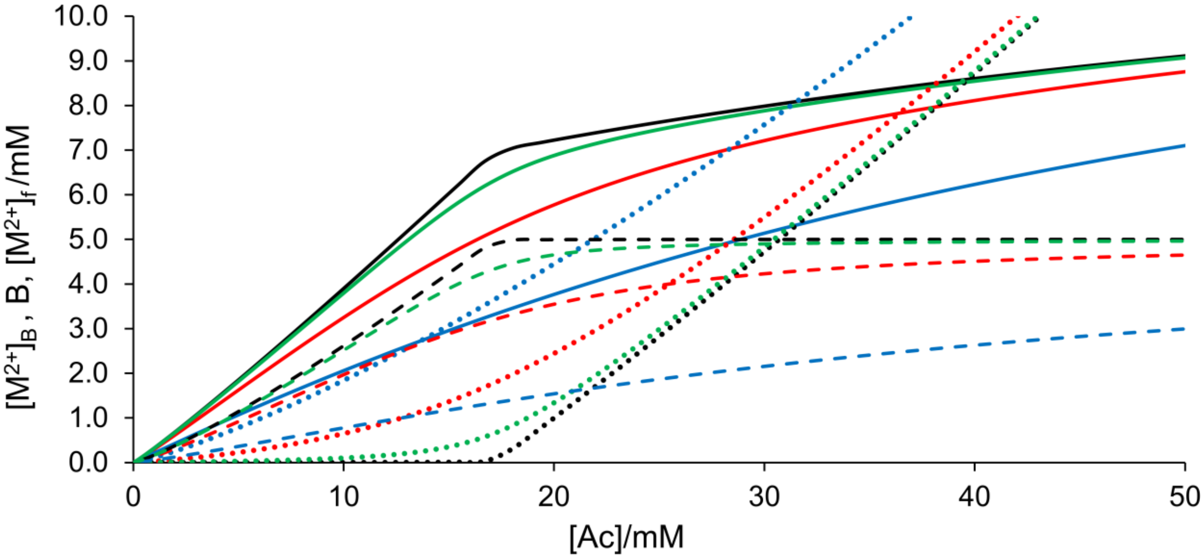

Figure S-16. Simulation of the diffusion of Ca^{2+} acetate through a solution of a polymer. $[M^{2+}]_f$ (dotted line), B (solid line), $[M^{2+}]_B$ (dashed line). K = 10^6 M^{-1} (black), 10^4 M^{-1} (green), 10^3 M^{-1} (red) and 10^2 M^{-1} (blue). In all plots $D_M/D_{M,NaCl}$ = 0.9, $[S]_{tot}$ = 5 mM, $D_{M,NaCl}$ = 9.3 x 10^{-10} m^2s^{-1}, h = 2 mm, r = 2.1 mm, m = 4.5 mg, M_r = 180 $gmol^{-1}$ (calcium acetate hydrate), t = 6 hours, D_{Ac} = 1 x 10^{-9} m^2s^{-1}.

S8. Effect of 50 mM NaCl on the binding behaviour observed by CSI

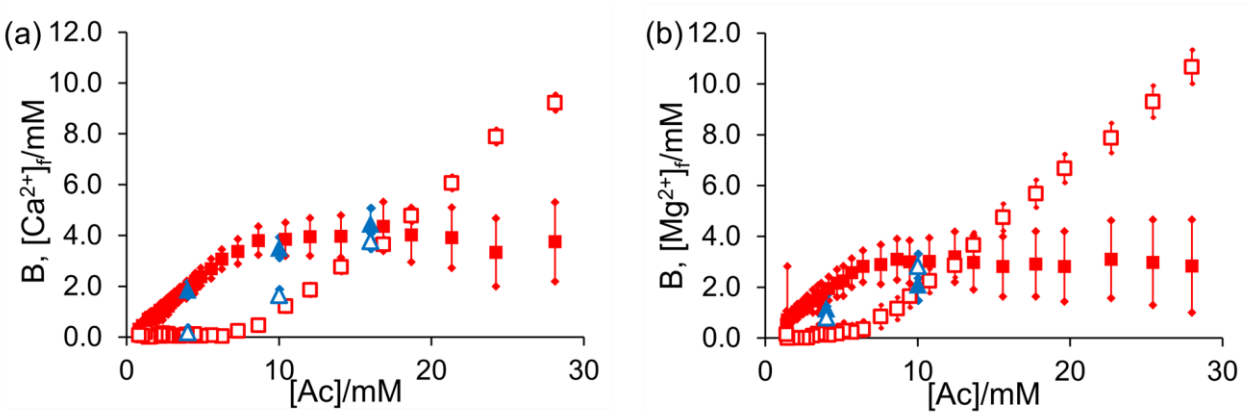

Figure S-17. Plot of B (solid symbols) and $[M^{2+}]_f$ (open symbols) when calcium acetate (a) or magnesium acetate (b) is diffused into solutions of 2 mg/mL alginate, in absence of 50 mM NaCl, and the sample analysed by CSI (red square), or mixed homogeneously with the alginate (blue triangle). B was calculated from Equation 4 with N = 0.

Without 50 mM NaCl, Mg^{2+} exhibits apparent strong binding to 2 mg/mL alginate by the CSI method (Figure S-17b). This strong binding is not observed by homogeneous mixing of alginate with Mg^{2+} acetate, or when 50 mM NaCl is present (Figure 4b). The apparent strong binding is attributable to the role of M^{2+} in balancing the negative charge of the alginate in the absence of the excess Na^+, as well as the exclusion of acetate ions.[56] Assuming the diffusion coefficient of an M^{2+} ion balancing the charge of an anionic polymer is 0 (Section S-7), D_M is given by Equation S27:

$$\frac{D_M}{D_{M,NaCl}} = 1 - \frac{C_p}{[M^{2+}]_{total}}\left(\frac{[M^{2+}]_{total}}{[Na^+]_{total} + 2[M^{2+}]_{total}}\right) \qquad \text{S27}$$

where C_p is the concentration of singly charged groups on the polymer and $[Na^+]_{total} = C_p + [NaCl]$. The factor 2 assumes that each M^{2+} ion balances the charge of two charged groups on the polymer. Plots of D_M/D_{NaCl} for 2 mg/mL alginate (C_p = 8 mM) calculated using Equation S27 are provided below on Figure S-18. Without 50 mM NaCl, a significant reduction in D_M is predicted that will give a positive value of B (Figure S-14), even in the absence of a significant binding effect.

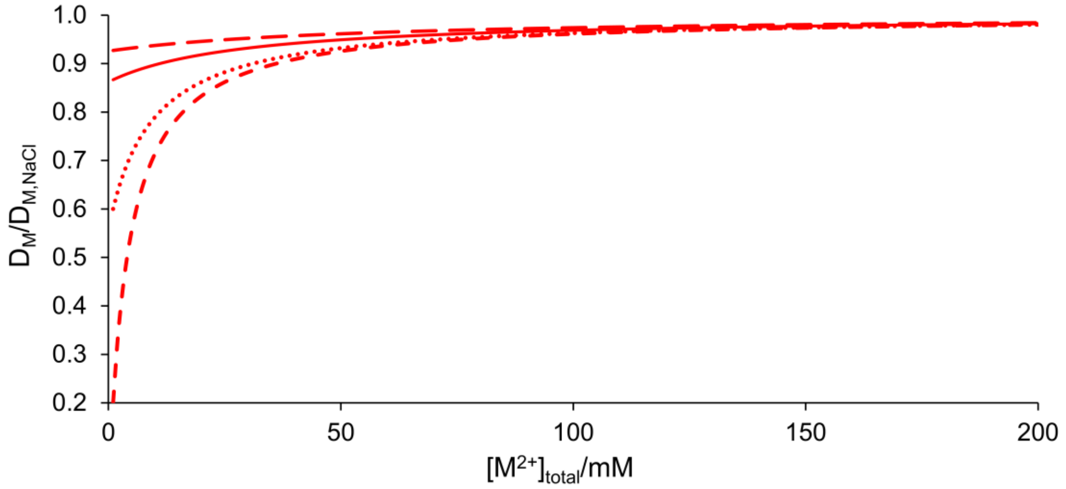

Figure S-18. Plot of $D_M/D_{M,NaCl}$ versus $[M^{2+}]_{total}$ calculated using Equation S27 for [NaCl] = 0 mM (short dash), 10 mM (dotted), 50 mM (solid line) and 100 mM (long dash).

S9. Plots of $[M^{2+}]_L$ versus [Ac] for samples of Figures 2 and 4

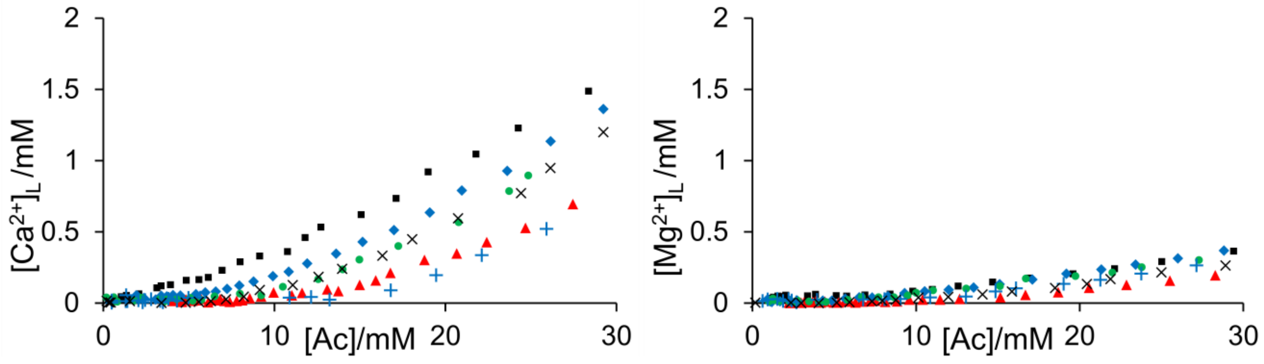

Figure S-20. Plots of $[M^{2+}]_L$ versus [Ac] in CSI experiments of Figures 2 and 4, calculated using Equation 2. PEI (black square), PAA (red triangle), PSS (blue diamond), 2 mg/mL sodium alginate (green circle), 4 mg/mL sodium alginate (blue vertical cross) and 2 wt% citrate-functionalised CNC (black cross). Ca^{2+} (left) and Mg^{2+} (right).

S10. Expanded plots from CSI datasets of citrate-functionalised CNC and 4 mg/mL sodium alginate, ^{1}H spectra of 0.5 wt% CNC in tap water

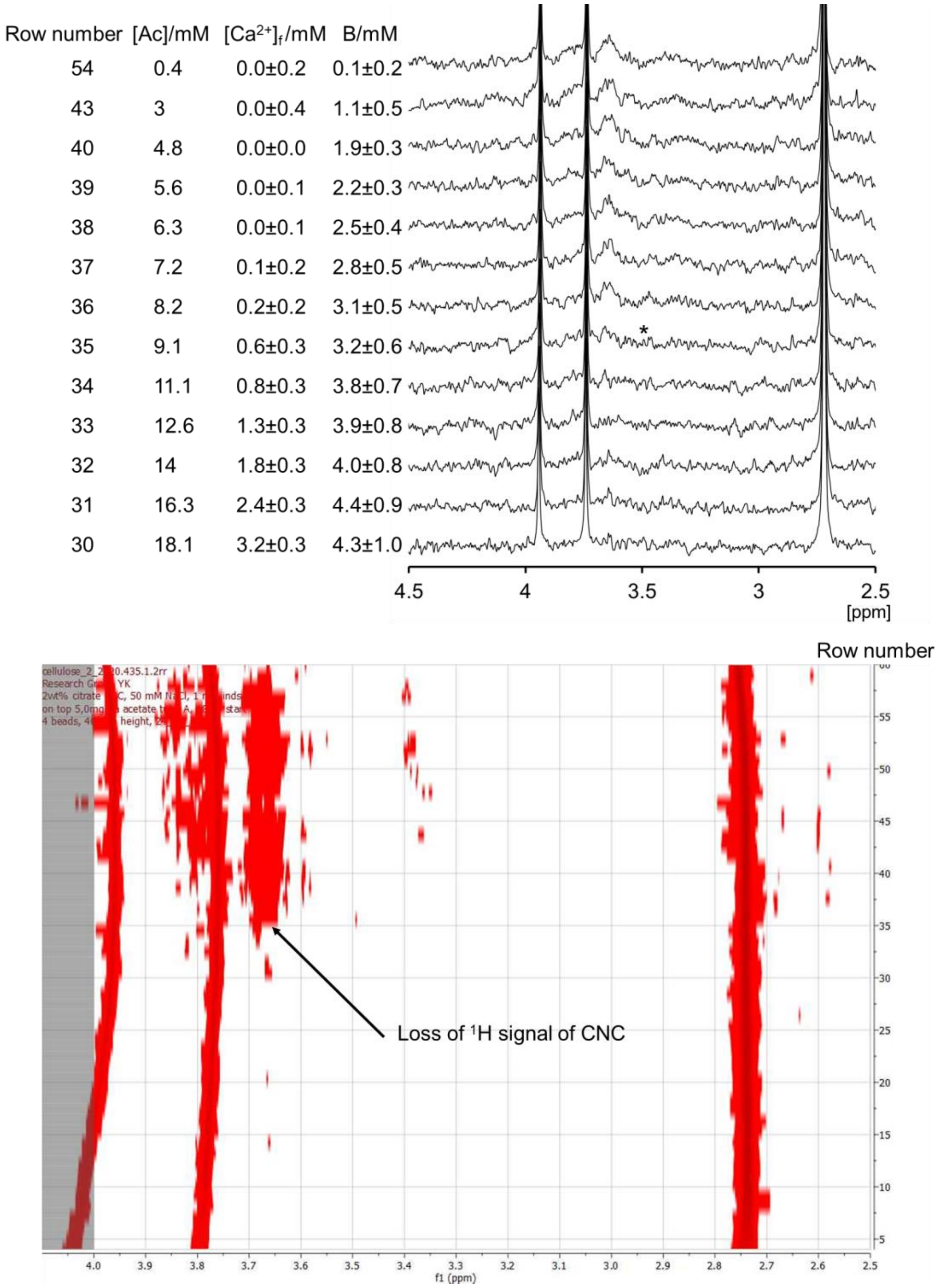

Figure S-21. Additional rows (top) and 2D plot (lower) of CSI dataset for diffusion of Ca^{2+} acetate into 2 wt% citrate-functionalised CNC (Figure 4c).

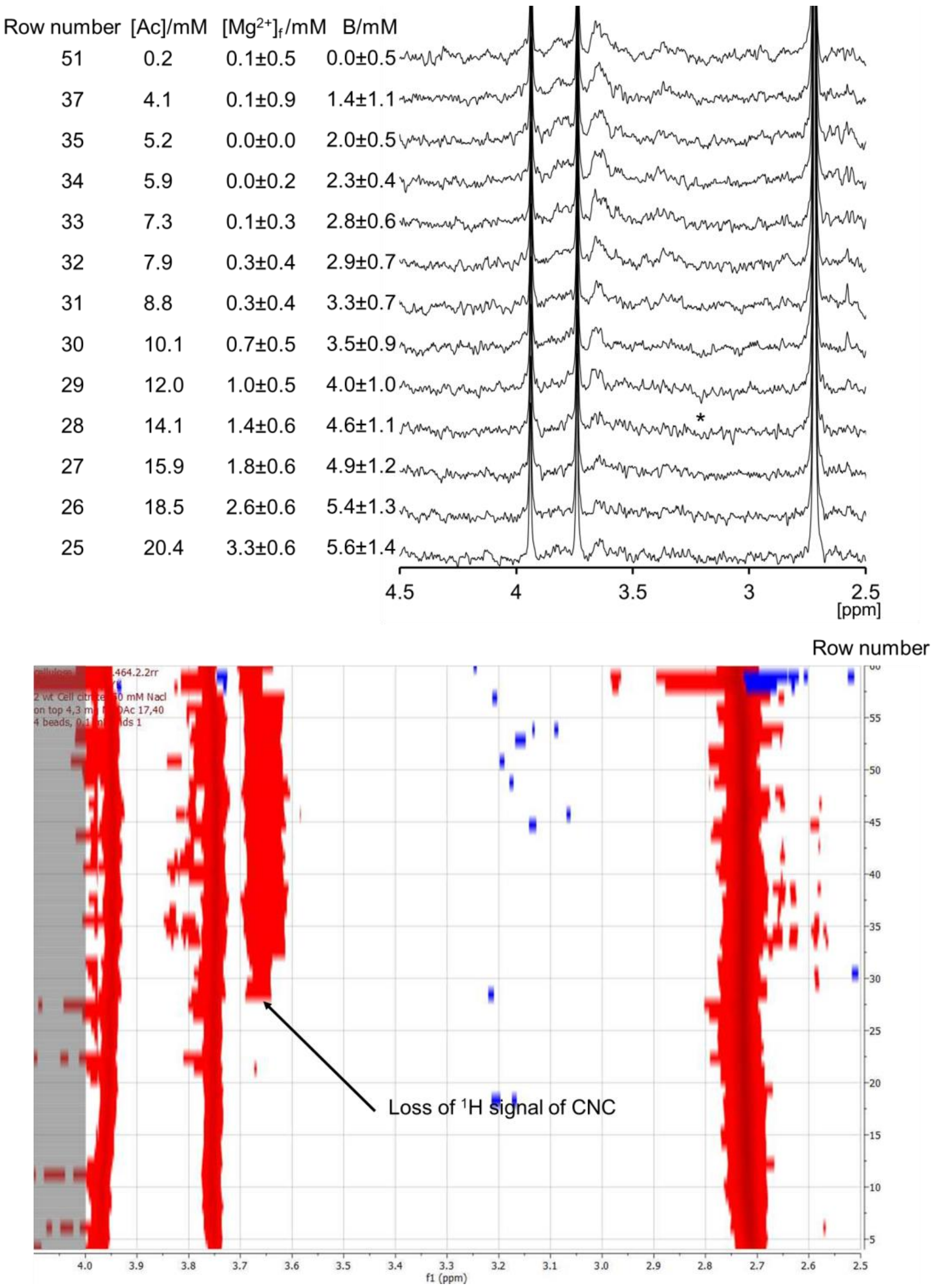

Figure S-22. Additional rows (top) and 2D plot (lower) of CSI dataset for diffusion of Mg^{2+} acetate into 2 wt% citrate-functionalised CNC (Figure 4c).

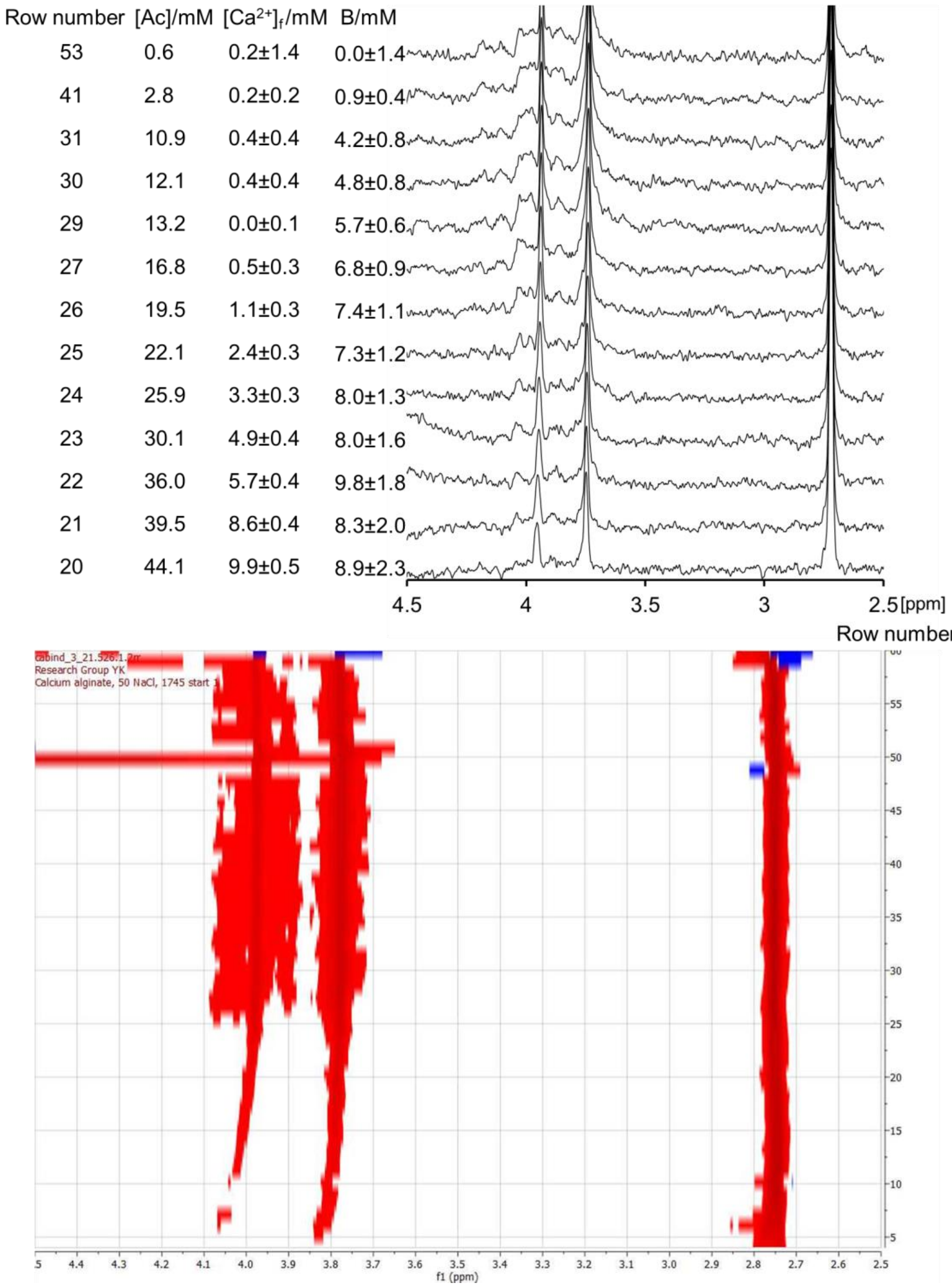

Figure S-23. Extracted ^{1}H spectra (top) and 2D plot (lower) of CSI dataset for diffusion of Ca^{2+} acetate into 4 mg/mL sodium alginate (Figure 4a).

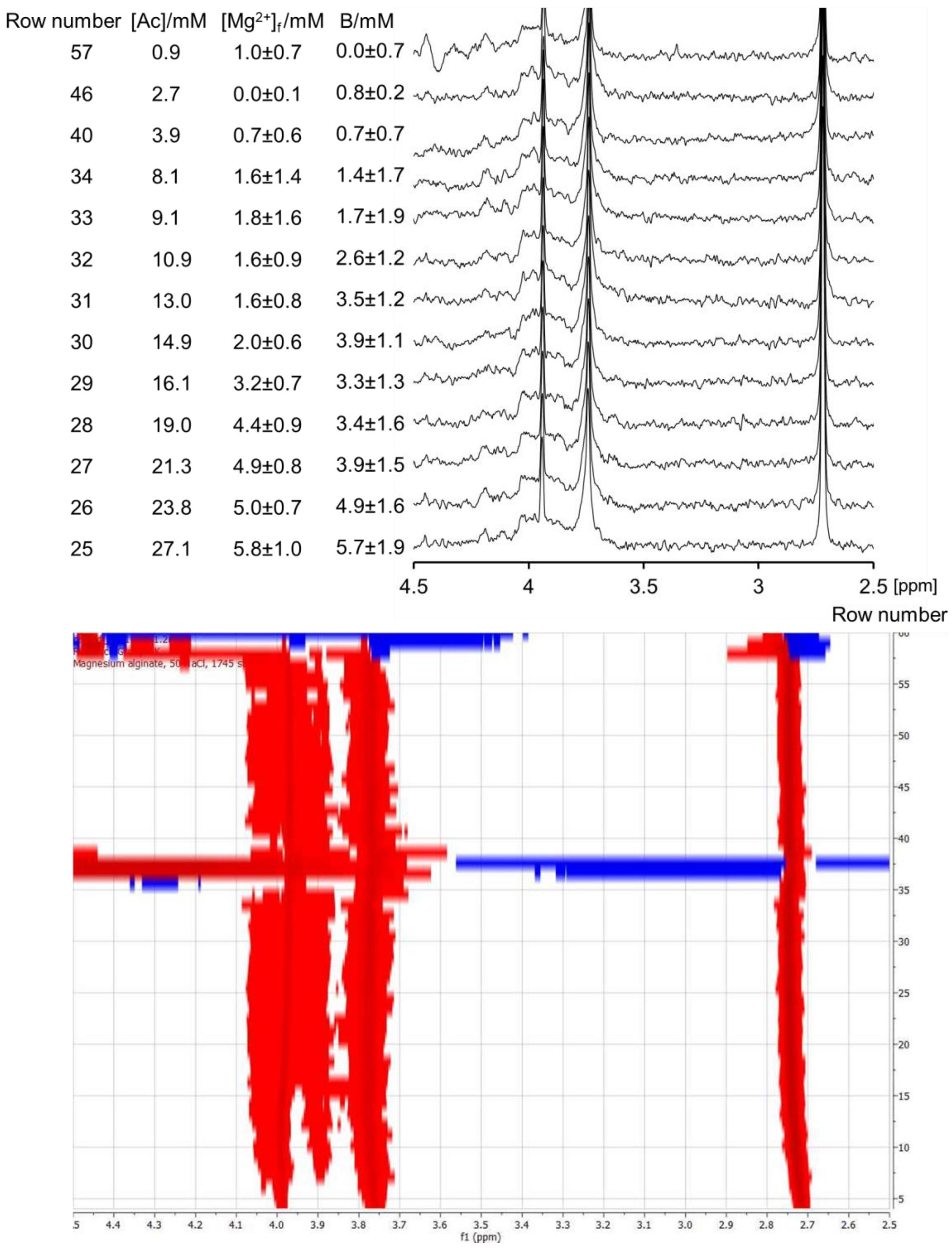

Figure S-24. Extracted ^{1}H spectra (top) and 2D plot (lower) of CSI dataset for diffusion of Mg^{2+} acetate into 4 mg/mL sodium alginate (Figure 4a). The distortion of rows 37 - 39 is due to an air bubble in the sample.

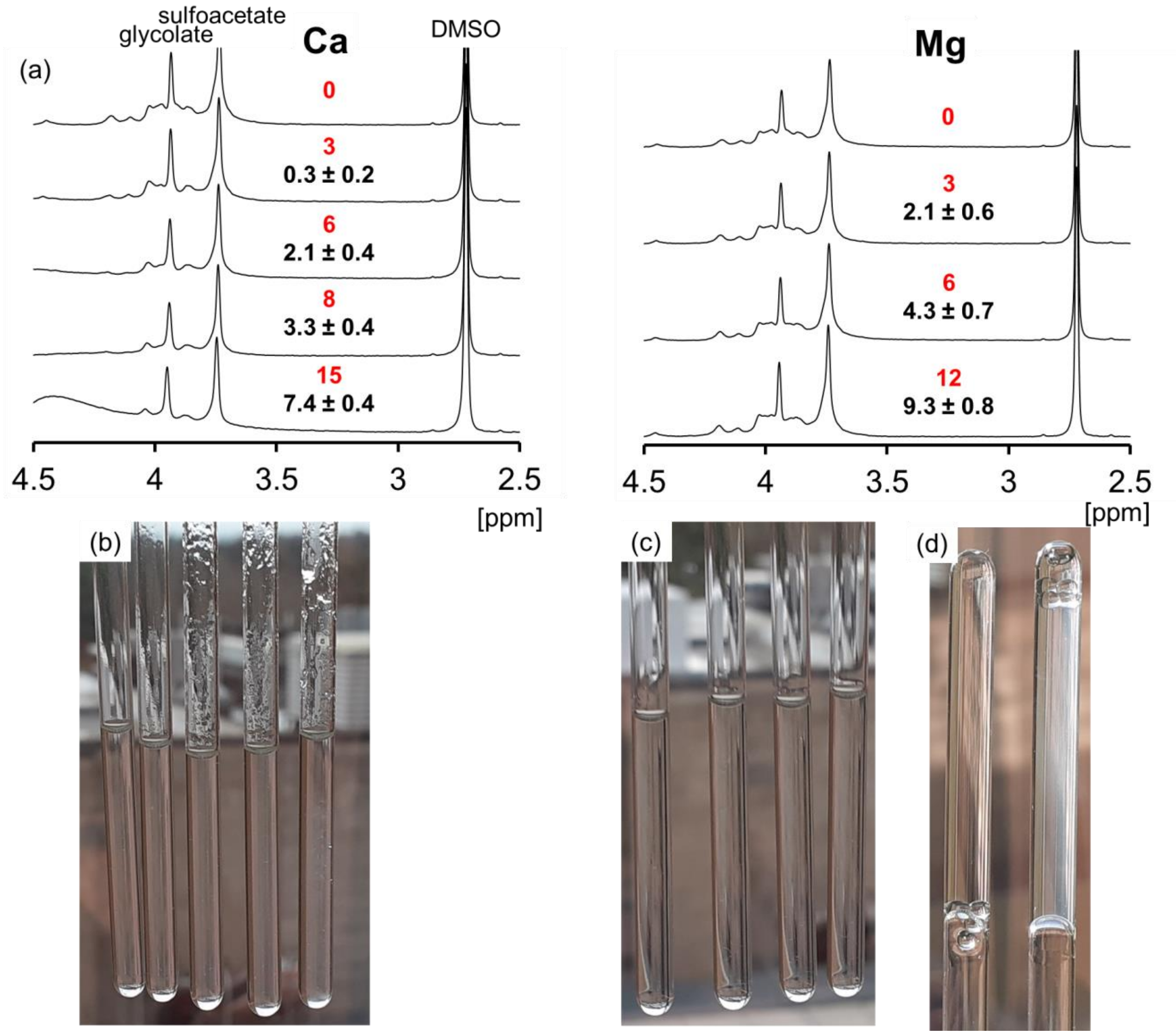

Figure S-25. (a) ^{1}H spectra of samples of 4 mg/mL sodium alginate with Ca^{2+} (left) and Mg^{2+} (right) prepared by direct mixing with M^{2+} acetate. $[M^{2+}]_{tot}$/mM (red) and $[M^{2+}]_{f}$/mM (black). Unless otherwise labelled, the ^{1}H resonances belong to the sample of alginate. Photographs of samples of 4 mg/mL sodium alginate and M^{2+}: (b) From left to right, $[Ca^{2+}]_{tot}$: 0, 3, 6, 8, 15 mM, (c) $[Mg^{2+}]_{tot}$: 0, 3, 6, 12 mM, (d) Mg^{2+} CSI sample (left) and Ca^{2+} CSI sample (right) 4 weeks after preparation. The samples had been kept in a water bath at 25 ˚C. The glass beads remain at the top of the Ca^{2+} sample when inverted, indicating the formation of a hydrogel. The beads fall in the Mg sample as a gel has not been formed.

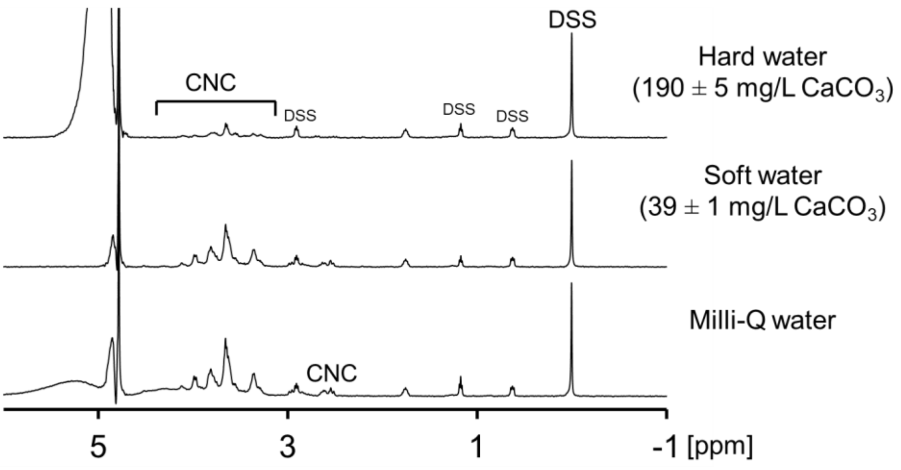

Figure S-26. ^{1}H spectra of 0.5 wt% citrate-functionalised CNC in hard, soft and Milli-Q water (Figure 4k). Spectra referenced to 0.1 mM DSS (0 ppm). Spectra were acquired off-lock in 64 scans using the perfect echo WATERGATE sequence of Adams et al.[57], incorporating the double echo W5 sequence of Liu et al.[58] The delay between successive pulses in the selective pulse train was set at 333 μs. The 90° pulse was set at 12 μs. The signal acquisition time and relaxation delay were 4.37 s and 1.0 s, respectively.

S11. Optical transmittance of CNC samples at 600 nm

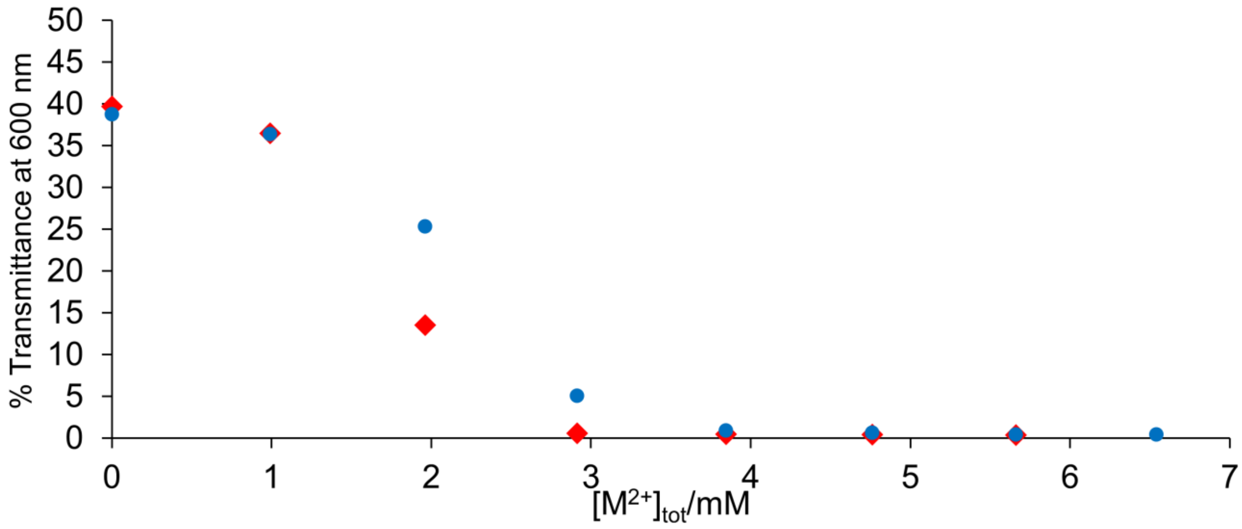

Figure S-27. Plot of optical transmittance of 2 wt% citrate-functionalised CNC with 50 mM NaCl, when titrated with $CaCl_2$ (red diamond) or $MgCl_2$ (blue circle). Samples were prepared in Brand disposable 10 mm microcuvettes (beam height intended as 8.5 mm) by placing 2 wt% citrate-functionalised CNC with 50 mM NaCl (700 μL sample volume) and adding aliquots (7 μL) of 0.1 M $CaCl_2$ or $MgCl_2$ by micropipette. The micropipette tip was used to stir the contents of the cuvette after each addition. Transmittance was measured at 600 nm using a PerkinElmer Lambda XLS UV/Vis Spectrometer. The cells were deliberately placed perpendicular to the beam to obtain a shorter path length (4 mm). Testing eight cuvettes, the uncertainty in transmittance introduced by placing the cells perpendicular to the beam was estimated as < 2 %. Distilled water was used as the reference (100% transmittance)

S12. Prediction of $[M^{2+}]_f$ in homogeneous samples of alginate and citrate-CNC using CSI data

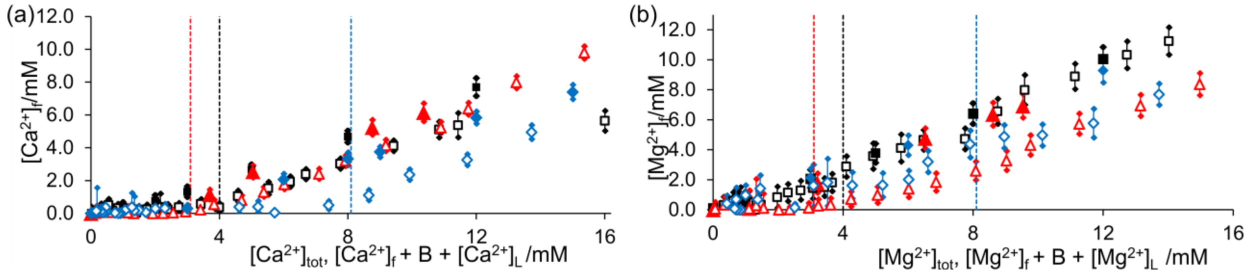

Figure S-28. Plots of $[M^{2+}]_f$ measured in CSI experiment versus $[M^{2+}]_f$ + B + $[M^{2+}]_L$ (open symbols), and $[M^{2+}]_f$ measured in homogeneous samples versus $[M^{2+}]_{tot}$ (solid symbols). 4 mg/mL alginate (blue diamond), 2 mg/mL alginate (black square) and 2 wt% citrate-CNC (red triangle). Ca (a) and Mg (b).

References

(50) Wallace, M.; Hicks, T.; Khimyak, Y. Z.; Angulo, J. Self-Correcting Method for the Measurement of Free Calcium and Magnesium Concentrations by 1H NMR. *Anal. Chem.* **2019**, *91* (22), 14442-14450, Article. DOI: 10.1021/acs.analchem.9b03008 Scopus.

(51) Wallace, M.; Lam, K.; Kuraite, A.; Khimyak, Y. Z. Rapid Determination of the Acidity, Alkalinity and Carboxyl Content of Aqueous Samples by 1H NMR with Minimal Sample Quantity. *Anal. Chem.* **2020**, *92* (19), 12789-12794.

(52) Tynkkynen, T.; Tiainen, M.; Soininen, P.; Laatikainen, R. From proton nuclear magnetic resonance spectra to pH. Assessment of 1H NMR pH indicator compound set for deuterium oxide solutions. *Anal. Chim. Acta* **2009**, *648* (1).

(53) Daniele, P. G.; Foti, C.; Gianguzza, A.; Prenesti, E.; Sammartano, S. Weak alkali and alkaline earth metal complexes of low molecular weight ligands in aqueous solution. *Coord. Chem. Rev.* **2008**, *252* (10-11), 1093-1107.

(54) Banerjee, P.; Yashonath, S.; Bagchi, B. Rotation driven translational diffusion of polyatomic ions in water: A novel mechanism for breakdown of Stokes-Einstein relation. *J. Chem. Phys.* **2017**, *146* (16).

(55) Monakhova, Y. B.; Kuballa, T.; Tschiersch, C.; Diehl, B. W. K. Rapid NMR determination of inorganic cations in food matrices: Application to mineral water. *Food Chem.* **2017**, *221*, 1828-1833.

(56) (a): Thomas Record, M.; Zhang, W.; Anderson, C. F. Analysis of Effects of Salts and Uncharged Solutes on Protein and Nucleic Acid Equilibria and Processes: A Practical Guide to Recognizing and Interpreting Polyelectrolyte Effects, Hofmeister Effects, and Osmotic Effects of Salts. In *Advances in Protein Chemistry*, Di Cera, E. Ed.; Vol. 51; Academic Press, 1998; pp 281-353. (b): Rouzina, I.; Bloomfield, V. A. Competitive electrostatic binding of charged ligands to polyelectrolytes: Practical approach using the non-linear Poisson-Boltzmann equation. *Biophysical Chemistry* **1997**, *64* (1-3), 139-155.

(57) Adams, R. W.; Holroyd, C. M.; Aguilar, J. A.; Nilsson, M.; Morris, G. A. “Perfecting” WATERGATE: Clean proton NMR spectra from aqueous solution. *ChemComm* **2013**, *49* (4), 358-360.

(58) Liu, M.; Mao, X. A.; Ye, C.; Huang, H.; Nicholson, J. K.; Lindon, J. C. Improved Watergate Pulse Sequences for Solvent Suppression in NMR Spectroscopy. *J. Magn. Reson.* **1998**, *132* (1), 125-129.

S13. Pulse programs and AU scripts

The row number (n) of a CSI dataset can be converted to vertical height from the base of the tube using Equation S28:

$$Z = \frac{\left(n - \frac{R}{2} - 0.5\right)\Delta_Z}{R} + Z_0 \qquad \text{S28}$$

where R is the total number of rows (64) and Z_0 is the height to the centre of the NMR-active window of our probe from the base of the tube (19 mm on our instrument). Δ_Z is the vertical range of the CSI experiment (26 mm).

S13.1 2D pulse sequence for CSI (Bruker)

```
;Modified from: "Probing spatial distribution of alignment by deuterium NMR imaging"
;Chemistry - A European Journal, Volume 19, Issue 22, 27 May 2013, Pages 7013-7019
;2D sequence for z-imaging preserving chemical shift
;using a phase encoding gradient. Original sequence written by Christian Merle, Martin Koos
;Modified to be on 1H with Excitation sculpting for water suppression
;Matthew Wallace, 3/2022 (University of East Anglia, matthew.wallace@uea.ac.uk)
;This pulse program is not fully tested and comes without warranty.
;!!This version is for running unlocked with sweep etc. disabled
;!!For running locked, delete the (UN)BLKGRAMP and insert the (UN)BLKGRAD
;statements as detailed in the comments
;Method for measuring free M2+ concentration can work in 10% D2O
;However, different limiting chemical shifts of glycolate and sulfoacetate must be used
;Wallace et al.  Anal. Chem. 2019, 91 (22), 14442-14450 doi.org/10.1021/acs.analchem.9b03008
;Set 1 SW to Z-range in mm (see cnst0) to get 1 Hz/mm scale in indirect dimension
;Make cnst0 bigger than actual sample size to avoid folding artefacts.
;Keep gpz6 at 100% and adjust cnst3 to get p30 to an acceptable length according to instrument (ca. 150-300 us)

; 1H-Version
;$CLASS=HighRes
;$DIM=2D
;$TYPE=
;$SUBTYPE=
;$COMMENT=
prosol relations=<triple>
#include <Avance.incl>
#include <Grad.incl>
#include <Delay.incl>
"cnst2= 0.8914027" ; integralfactor of gradient shape SMSQ10.32
"cnst4= 267.52220" ; * 10^6 /Ts = gamma1H
"p30=(td1/cnst0)*(1/(cnst1*cnst2*cnst3))*(1/cnst4)*(2*3.14159265/1000)*0.5 s"
"l1=td1-1"
lgrad r1d = l1
"acqt0=0"
"DELTA1=p30+d16"
"TAU=de+p1*2/3.1416+50u"
"p2=p1*2"
"d12=20u"
"d4=d1-100m"
baseopt_echo

1 ze
2 30m ;BLKGRAD so locked during d1
d1
;spoil gradient from previous acquisition
3 50u UNBLKGRAMP ;UNBLKGRAD
p19:gp3
d16
;start of zgesgp
d12 pl1:f1
  p1 ph1
  50u
  p16:gp1
  d16 pl0:f1
  (p12:sp1 ph2:r):f1
  4u
  d12 pl1:f1

  p2 ph3

  4u
  p16:gp1
  d16
  TAU
DELTA1
  p16:gp2
  d16 pl0:f1
  (p12:sp1 ph4:r):f1
  4u
  d12 pl1:f1

  p2 ph5

  4u
  p16:gp2
  d16
p30:gp6*cnst3*r1d
```

```
d16 BLKGRAMP ;remove this BLKGRAMP statement if running locked
go=2 ph31
30m
100m wr #0 if #0 zd igrad r1d
d4
lo to 3 times l1
goto 5
; run last increment:
4 30m ;BLKGRAD
d1
;spoil gradient from previous
5 50u UNBLKGRAMP ;UNBLKGRAD
p19:gp3
d16
  ;start of zgesgp
d12 pl1:f1
  p1 ph1
  50u
  p16:gp1
  d16 pl0:f1
  (p12:sp1 ph2:r):f1
  4u
  d12 pl1:f1

  p2 ph3

  4u
  p16:gp1
  d16
  TAU
DELTA1
  p16:gp2
  d16 pl0:f1
  (p12:sp1 ph4:r):f1
  4u
  d12 pl1:f1

  p2 ph5

  4u
  p16:gp2
  d16
p30:gp6*cnst3*r1d
d16 BLKGRAMP ;remove this BLKGRAMP statement if running locked
go=4 ph31
30m ;BLKGRAD
100m wr #0 if #0 zd
d3
exit
;Phase cycling as for zgesgp
ph1=0
ph2=0 1
ph3=2 3
ph4=0 0 1 1
ph5=2 2 3 3
ph31=0 2 2 0

;pl0 : 0W
;cnst0 : z-Range in cm
;cnst1 : GCC (G/mm) from Gradpar
;cnst3 : set max to get acceptable length of GP of ca 150-300us [0.95 max]
;pl1 : f1 channel - power level for pulse (default)
;sp1 : f1 channel - shaped pulse 180 degree
;p1 : f1 channel - 90 degree high power pulse
;p12: f1 channel - 180 degree shaped pulse (Gaus1.1000)   [4 msec]
;p16: homospoil/gradient pulse for water suppression (1000 us)
;p19 : Spoil gradient pulse (1000 us)
;gpz6: 100% phase encoding gradient
;d1 :  Relaxation delay
;d3: waiting time before next experiment in a time delay sequence [s] set to 0.1 if no delay
;d12: delay for power switching                    [20 usec]
;d16: standard eddy delay (200u)
;ns: 4 * n, total number of scans: NS * TD0
;ds: 2*m [16]
;td1: number of experiments
;FnMODE: QF
;use gradient ratio:    gp 1 : gp 2
;                         31 :   11
;for z-only gradients:
;gpz1: 31%
;gpz2: 11%
;gpz3: 50%
;use gradient files:

;gpnam1: SMSQ10.100
;gpnam2: SMSQ10.100
;gpnam3: SMSQ10.100
;gpnam6: SMSQ10.32

;$Id: zgesgpimg,v 1.9 2012/01/31 17:49:32 ber Exp $
```

S13.2 AU program to process raw CSI datasets (Bruker)

```
/*************************************************-*-C++-*-********/
/*              proc_img*/
/*Default processing of CSI image in Bruker Topspin*/
/*Set as variable AUNMP for auto processing with IconNMR*/
/*Each row (1D spectrum) will require individual phase and baseline correction*/
/*Using script 13.3 provided in this work*/
```

```
/*Line broadening factor (LB) is set as 3 by default below*/
/*This AU is not fully tested and comes without warranty.*/
/*Matthew Wallace, 3/2022*/
/*University of East Anglia, matthew.wallace@uea.ac.uk*/
/*******************************************************************/
int np=128;
int si=32768;
float ph1;
float phd=0;
int wd=3;
float lb=3.0;
float sb=0;

/*Want phase sensitive processsing in F1 and F2*/
STOREPAR1("PH_mod",1)
STOREPAR("PH_mod",1)
/*Need 1st order correction in F1 of 180*number of slices in image*/
FETCHPAR1S("SI",&np)
ph1=np*180;
STOREPAR1("PHC1",ph1)
/*Remove any default phasing*/
STOREPAR("PHC0",phd)
STOREPAR("PHC1",phd)
/*Sine function in F1*/
STOREPAR1("WDW",wd)
STOREPAR1("SSB",sb)
/*Exponential multiplication in f2 default LB of 3*/
STOREPAR("WDW",1)
STOREPAR("LB",lb)
STOREPAR("SI",si)
XFB
ERRORABORT
QUIT
```

S13.3 AU program to phase and baseline correct CSI datasets (Bruker)

```
/*To produce phase corrected chemical shift image from gradient encoded data*/
/*Set 1st order phase correction for f1 dimension to 180*Td1 (11520 when 64 points in image), 0th order to 0*/
/*PH_mod should be set to PK in both dimensions*/
/*XFB to produce image*/
/*This is done automatically by script 13.2 above*/
/*With the 2D dataset selected, Run this Au*/
/*AU extracts each row in turn to a procno and automatically phase and baseline corrects/*
/*Reversal of F1 axis may be necessary, depending on NMR probe*/
/*This AU is not fully tested and comes without warranty.*/
/*The script works on Bruker Topspin 3.6.2 but has not been tested on other versions*/
/*Use kill command if all goes wrong*/
/*Matthew Wallace, 3/2022*/
/*University of East Anglia, matthew.wallace@uea.ac.uk*/
char disk1[32], user1[32], location[128], phtyp[8];
float abf1=8;
float abf2=6;
int phpno=1;
int w=1;
int np=64;
int pno=5;
GETCURDATA
int steno=expno;
strcpy(location,disk);
strcpy(phtyp,"k");
GETSTRING("Enter location of dataset",location)
phpno=procno;
GETINT("Enter experiment number to process",steno)
GETINT("Enter procno containing XFB processed 2D data :",phpno)
pno=phpno+5;
GETINT("Enter procno to write rows to phase and baseline correct (empty):",pno)
REXPNO(steno)
RPROCNO(phpno)
SETCURDATA
FETCHPAR1("SI",&np)
GETINT("Enter number of points in image (autodetects) :",np)
GETSTRING("APKS (s) or APK (k) or apkf (f) auto phase correction?",phtyp)
if(strcmp(phtyp,"f")==0)
{
GETFLOAT("Enter right limit for apkf and absf:",abf2)
GETFLOAT("Enter left limit for apkf and absf:",abf1)
}
w=1;
TIMES(np)
{
RPROCNO(phpno)
SETCURDATA
RSR(w,pno)
RPROCNO(pno)
SETCURDATA
        if(strcmp(phtyp,"s")==0)
        {
APKS
ABS
        }
        if(strcmp(phtyp,"k")==0)
        {
APK
ABS
        }
        if(strcmp(phtyp,"f")==0)
        {
                STOREPAR("absf1",abf1)
                STOREPAR("absf2",abf2)
```

```
APKF
ABSF
            }
            WSR(w,phpno,steno,name,user,location)
w++;
            }
            END
            QUIT
```

S13.4 AU program to extract chemical shifts from CSI dataset (Bruker)

```
/*Bruker AU script for extracting peak positions from a CSI dataset*/
/*CSI dataset should have been fully processed in phase-sensitive mode*/
/*Rough chemical shift referencing in F2 also helps*/
/*The script works on Bruker Topspin 3.6.2 but has not been tested on other versions*/
/*The script extracts each row in turn into the empty procno requested (will overwrite existing contents!!!)*/
/*Will write peak positions of glycolate, SFA, 2MI, ref and acetate to separate .txt files/ppm  in procno directory of CSI dataset*/
/*These numbers go from first to final row of the dataset*/
/*Numbers can be copied and pasted into spreadsheet*/
/*Peak picking routine will find the centre of a multiplet (doublet, quartet, or singlet with bad shim)*/
/*Will treat two peaks as outer edges of a multiplet if their intensity is within ppsens of the biggest peak found in specified range*/
/*Adjust peak picking ranges as appropriate to avoid overlap*/
/*Default values here apply to DMSO as chemical shift reference*/
/*This AU is not fully tested and comes without warranty.*/
/*Use kill command if all goes wrong*/
/*Matthew Wallace, 3/2022*/
/*University of East Anglia, matthew.wallace@uea.ac.uk*/
FILE *fglyc,*fref,*fac,*fsfa,*fmi;
float min=0;
double f2pref=2.5;
double f1pref=2.8;
double f2pglyc=3.85;
double f1pglyc=4.2;
double f2pac=1.8;
double f1pac=2.18;
double f2mi=6;
double f1mi=9;
double f2sfa=3.65;
double f1sfa=3.85;
float ppsens=0.9;
double pc=0.1;
int steno=15;
int eno;
int ne=5;
int m=-1;
int rn=1;
double peakFreqHz, peakFreqPPM, peakIntensity, maxpsh, maxpsp, maxips, sf,sfo1,so1p;
double mintpp,minpsp,peakppmneg,cent,ppmdif,maxpspneg;
int i, numPeaks;
int np=64;
int row=1;
int v=1;
int wrpno=5;
int phpno=1;
GETCURDATA
steno=expno;
phpno=procno;
GETINT("Enter experiment number of CSI dataset",steno)
GETINT("Enter procno of CSI dataset",phpno)
REXPNO(steno)
RPROCNO(phpno)
SETCURDATA
FETCHPAR1("td",&np)
GETINT("Enter number of gradient values",np)
GETINT("Enter procno to extract rows into for peak picking (blank)",wrpno)
GETDOUBLE("Reference right peak picking/ppm",f2pref)
GETDOUBLE("Reference left peak picking/ppm",f1pref)
GETDOUBLE("Glycolate right peak picking/ppm",f2pglyc)
GETDOUBLE("Glycolate left peak picking/ppm",f1pglyc)
GETDOUBLE("Acetate right peak picking/ppm",f2pac)
GETDOUBLE("Acetate left peak picking/ppm",f1pac)
GETDOUBLE("Sulfoacetate right peak picking/ppm",f2sfa)
GETDOUBLE("Sulfoacetate left peak picking/ppm",f1sfa)
GETDOUBLE("2-methylimidazole right peak picking/ppm",f2mi)
GETDOUBLE("2-methylimidazole left peak picking/ppm",f1mi)
GETFLOAT("Enter peak picking sensitivity factor",pc)
GETDOUBLE("Enter satellite sensitivity factor",ppsens)
STOREPAR("mi",min)
STOREPAR("pc",pc)
/*Global scaling*/
STOREPAR("pscal",0)
/*Creates text files to hold peak positions*/
if ((fref = fopen(PROCPATH("Reference chemical shift.txt"),"wt")) == 0)
   STOPMSG("Cannot create file")
   if ((fglyc = fopen(PROCPATH("Glycolate.txt"),"wt")) == 0)
   STOPMSG("Cannot create file")
   if ((fac = fopen(PROCPATH("Acetate.txt"),"wt")) == 0)
   STOPMSG("Cannot create file")
    if ((fmi = fopen(PROCPATH("2-methylimidazole.txt"),"wt")) == 0)
   STOPMSG("Cannot create file")
    if ((fsfa = fopen(PROCPATH("sulfoacetate.txt"),"wt")) == 0)
   STOPMSG("Cannot create file")
  /*No go through each row in turn and extract into requested procno, find peak positions*/
TIMES(np)
{
RSR(v,wrpno)
RPROCNO(wrpno)
SETCURDATA
/*Extract chemcial shift of reference peak first*/
STOREPAR("f2p",f2pref)
```

```
STOREPAR("f1p",f1pref)
PP
 numPeaks = readPeakList(PROCPATH(0));
maxips=0.0;
    maxpsh=0.0;
    for (i=0; i<numPeaks; i++)
    {
                peakIntensity = getPeakIntensity(i);
                peakFreqHz = getPeakFreqHz(i);
                peakFreqPPM = getPeakFreqPPM(i);
                if (peakIntensity > maxips)
                {
                    maxips = peakIntensity;
                    maxpsh = peakFreqHz;
                    maxpsp = peakFreqPPM;
                }
    }
    /*Pick most downfield side of multiplet*/
    mintpp=maxips*ppsens;
maxpsp=0.0;
    for (i=0; i<numPeaks; i++)
    {
                                peakIntensity = getPeakIntensity(i);
                                if(peakIntensity>mintpp)
                                {
                peakFreqPPM = getPeakFreqPPM(i);
                peakFreqHz = getPeakFreqHz(i);
                if (peakFreqHz >= maxpsh)
                {
                    maxpsp = peakFreqPPM;
                    maxpsh = peakFreqHz;
                }
    }
                }
/*Flips negative to choose most upfield peak of multiplet*/
for (i=0; i<numPeaks; i++)
    {
                                peakIntensity = getPeakIntensity(i);
                                if(peakIntensity>mintpp)
                                {
                peakFreqPPM = getPeakFreqPPM(i);
peakppmneg=peakFreqPPM*m;
maxpspneg=maxpsp*m;
                if (peakppmneg >= maxpspneg)
                {
                    minpsp = peakFreqPPM;
                }
    }
                }
    freePeakList();
/*writes chemical shift into text document*/
ppmdif=maxpsp-minpsp;
cent=minpsp+ppmdif*0.5;
    fprintf(fref,"%f \n",cent);
/*Reads in for Glycolate*/
STOREPAR("f2p",f2pglyc)
STOREPAR("f1p",f1pglyc)
STOREPAR("mi",min)
PP
 numPeaks = readPeakList(PROCPATH(0));
maxips=0.0;
    maxpsh=0.0;
    for (i=0; i<numPeaks; i++)
    {
                peakIntensity = getPeakIntensity(i);
                peakFreqHz = getPeakFreqHz(i);
                peakFreqPPM = getPeakFreqPPM(i);
                if (peakIntensity > maxips)
                {
                    maxips = peakIntensity;
                    maxpsh = peakFreqHz;
                    maxpsp = peakFreqPPM;
                }
    }
 /*Pick most downfield side of multiplet*/
    mintpp=maxips*ppsens;
maxpsp=0.0;
    for (i=0; i<numPeaks; i++)
    {
                                peakIntensity = getPeakIntensity(i);
                                if(peakIntensity>mintpp)
                                {
                peakFreqPPM = getPeakFreqPPM(i);
                peakFreqHz = getPeakFreqHz(i);
                if (peakFreqHz >= maxpsh)
                {
                    maxpsp = peakFreqPPM;
                    maxpsh = peakFreqHz;
                }
    }
                }
/*Flips negative to choose most upfield peak of multiplet*/
for (i=0; i<numPeaks; i++)
    {
                                peakIntensity = getPeakIntensity(i);
                                if(peakIntensity>mintpp)
                                {
                peakFreqPPM = getPeakFreqPPM(i);
peakppmneg=peakFreqPPM*m;
maxpspneg=maxpsp*m;
                if (peakppmneg >= maxpspneg)
```

```
                {
                    minpsp = peakFreqPPM;
                }
    }
                }
    freePeakList();
/*writes chemical shift into text document*/
ppmdif=maxpsp-minpsp;
cent=minpsp+ppmdif*0.5;
    fprintf(fglyc,"%f \n",cent);
/*Reads in for Acetate*/
STOREPAR("f2p",f2pac)
STOREPAR("f1p",f1pac)
PP
 numPeaks = readPeakList(PROCPATH(0));
maxips=0.0;
    maxpsh=0.0;
    for (i=0; i<numPeaks; i++)
    {
                peakIntensity = getPeakIntensity(i);
                peakFreqHz = getPeakFreqHz(i);
                peakFreqPPM = getPeakFreqPPM(i);
                if (peakIntensity > maxips)
                {
                    maxips = peakIntensity;
                    maxpsh = peakFreqHz;
                    maxpsp = peakFreqPPM;
                }
    }
 /*Pick most downfield side of multiplet*/
    mintpp=maxips*ppsens;
maxpsp=0.0;
    for (i=0; i<numPeaks; i++)
    {
                                peakIntensity = getPeakIntensity(i);
                                if(peakIntensity>mintpp)
                                {
                peakFreqPPM = getPeakFreqPPM(i);
                peakFreqHz = getPeakFreqHz(i);
                if (peakFreqHz >= maxpsh)
                {
                    maxpsp = peakFreqPPM;
                    maxpsh = peakFreqHz;
                }
    }
                }
/*Flips negative to choose most upfield peak of multiplet*/
for (i=0; i<numPeaks; i++)
    {
                                peakIntensity = getPeakIntensity(i);
                                if(peakIntensity>mintpp)
                                {
                peakFreqPPM = getPeakFreqPPM(i);
peakppmneg=peakFreqPPM*m;
maxpspneg=maxpsp*m;
                if (peakppmneg >= maxpspneg)
                {
                    minpsp = peakFreqPPM;
                }
    }
                }
    freePeakList();
/*writes chemical shift into text document*/
ppmdif=maxpsp-minpsp;
cent=minpsp+ppmdif*0.5;
    fprintf(fac,"%f \n",cent);
/*Reads in for sulfoacetate*/
STOREPAR("f2p",f2sfa)
STOREPAR("f1p",f1sfa)
PP
 numPeaks = readPeakList(PROCPATH(0));
maxips=0.0;
    maxpsh=0.0;
    for (i=0; i<numPeaks; i++)
    {
                peakIntensity = getPeakIntensity(i);
                peakFreqHz = getPeakFreqHz(i);
                peakFreqPPM = getPeakFreqPPM(i);
                if (peakIntensity > maxips)
                {
                    maxips = peakIntensity;
                    maxpsh = peakFreqHz;
                    maxpsp = peakFreqPPM;
                }
    }
 /*Pick most downfield side of multiplet*/
    mintpp=maxips*ppsens;
maxpsp=0.0;
    for (i=0; i<numPeaks; i++)
    {
                                peakIntensity = getPeakIntensity(i);
                                if(peakIntensity>mintpp)
                                {
                peakFreqPPM = getPeakFreqPPM(i);
                peakFreqHz = getPeakFreqHz(i);
                if (peakFreqHz >= maxpsh)
                {
                    maxpsp = peakFreqPPM;
                    maxpsh = peakFreqHz;
                }
    }
```

```
                }
/*Flips negative to choose most upfield peak of multiplet*/
for (i=0; i<numPeaks; i++)
    {
                                    peakIntensity = getPeakIntensity(i);
                                    if(peakIntensity>mintpp)
                                    {
                peakFreqPPM = getPeakFreqPPM(i);
peakppmneg=peakFreqPPM*m;
maxpspneg=maxpsp*m;
                if (peakppmneg >= maxpspneg)
                {
                    minpsp = peakFreqPPM;
                }
    }
                }
    freePeakList();
/*writes chemical shift into text document*/
ppmdif=maxpsp-minpsp;
cent=minpsp+ppmdif*0.5;
    fprintf(fsfa,"%f \n",cent);
/*Reads in for 2-methylimidazole*/
STOREPAR("f2p",f2mi)
STOREPAR("f1p",f1mi)
PP
 numPeaks = readPeakList(PROCPATH(0));
maxips=0.0;
    maxpsh=0.0;
    for (i=0; i<numPeaks; i++)
    {
                peakIntensity = getPeakIntensity(i);
                peakFreqHz = getPeakFreqHz(i);
                peakFreqPPM = getPeakFreqPPM(i);
                if (peakIntensity > maxips)
                {
                    maxips = peakIntensity;
                    maxpsh = peakFreqHz;
                    maxpsp = peakFreqPPM;
                }
    }
 /*Pick most downfield side of multiplet*/
    mintpp=maxips*ppsens;
maxpsp=0.0;
    for (i=0; i<numPeaks; i++)
    {
                                    peakIntensity = getPeakIntensity(i);
                                    if(peakIntensity>mintpp)
                                    {
                peakFreqPPM = getPeakFreqPPM(i);
                peakFreqHz = getPeakFreqHz(i);
                if (peakFreqHz >= maxpsh)
                {
                    maxpsp = peakFreqPPM;
                    maxpsh = peakFreqHz;
                }
    }
                }
/*Flips negative to choose most upfield peak of multiplet*/
for (i=0; i<numPeaks; i++)
    {
                                    peakIntensity = getPeakIntensity(i);
                                    if(peakIntensity>mintpp)
                                    {
                peakFreqPPM = getPeakFreqPPM(i);
peakppmneg=peakFreqPPM*m;
maxpspneg=maxpsp*m;
                if (peakppmneg >= maxpspneg)
                {
                    minpsp = peakFreqPPM;
                }
    }
                }
    freePeakList();
/*writes chemical shift into text document*/
ppmdif=maxpsp-minpsp;
cent=minpsp+ppmdif*0.5;
    fprintf(fmi,"%f \n",cent);
v++;
RPROCNO(phpno)
SETCURDATA
}
END
fclose(fref);
fclose(fac);
fclose(fglyc);
fclose(fsfa);
fclose(fmi);
QUIT
```

S13.5 AU program to extract integrals of acetate and reference from a CSI dataset (Bruker)

```
/*Bruker AU script for integrating a CSI dataset*/
/*CSI dataset should have been fully processed in phase-sensitive mode*/
/*Crude chemical shift referencing in F2 also helps*/
/*The script works on Bruker Topspin 3.6.2 but has not been tested on other versions*/
/*Create a 1D integral file using the wmisc command*/
/*This should contain only the reference compound and acetate*/
/*Copy and paste this name into the text box when asked, or paste into strcpy(text,"dmsim"); line 45 below, in place of dmsim */
/*dmsim is the name of the integral range file used in this script by default*/
```

```
/*The script extracts each row in turn into the empty procno requested (will overwrite existing contents!!!)*/
/*Working in this procno, the spectrum is referenced to the chemical shift reference supplied*/
/*Default values here apply to DMSO, DMSO signal is downfield of acetate (ordans=d)*/
/*Change right and left peak picking limits if any risk of a non-reference peak being included in the referencing procedure*/
/*Having referenced the spectrum, the requested integral file is read in and the integral values exported to .txt documents*/
/*Each .txt document is stored in the procno directory of the 2D CSI dataset*/
/*The script will also by default save rows 15, 32 and 50 into procnos of the same number*/
/*This is so the work of the script can be checked - spectra are correctly referenced and integrals correctly applied*/
/*This AU is not fully tested and comes without warranty.*/
/*Use kill command if all goes wrong*/
/*Matthew Wallace, 3/2022*/
/*University of East Anglia, matthew.wallace@uea.ac.uk*/
FILE    *fpnt,*fref,*fac;
char savans[8],dummystr[256],intdir[256],location[128],ordans[8];
float min=0;
double f2pdmso=2.65;
double f1pdmso=2.8;
double ref=2.72;
float ppsens=0.9;
double pc=0.1;
int m=-1;
int rn=1;
int sav1=15;
int sav2=32;
int sav3=50;
int steno;
double peakFreqHz, peakFreqPPM, peakIntensity, maxpsh, maxpsp, maxips, sf,sfn,sfo1,intgr;
double intnum,ppmdn,ppmup,intgrso1p,mintpp,minpsp,peakppmneg,cent,ppmdif,maxpspneg;
int i, numPeaks;
int np=64;
int row=1;
int v=1;
int wrpno=5;
int linenum=1;
int phpno=1;
strcpy(location,disk);
strcpy(savans,"y");
strcpy(text,"dmsim");
strcpy(ordans,"d");
GETCURDATA
steno=expno;
phpno=procno;
GETINT("Enter experiment number of 2D dataset",steno)
GETINT("Enter procno of 2D dataset",phpno)
REXPNO(steno)
RPROCNO(phpno)
SETCURDATA
FETCHPAR1("td",&np)
GETINT("Enter number of spectra in CSI image",np)
GETINT("Enter procno to extract rows into for integration (blank)",wrpno)
GETDOUBLE("Chemical shift reference right peak picking/ppm",f2pdmso)
GETDOUBLE("Chemical shift reference left peak picking/ppm",f1pdmso)
GETDOUBLE("Enter reference shift/ppm",ref)
GETFLOAT("Enter peak picking sensitivity factor",pc)
GETDOUBLE("Enter satellite sensitivity factor",ppsens)
GETSTRING("Save example spectra to check working OK? y/n",savans)
if(strcmp(savans,"y")==0)
{
GETINT("Enter 1st spectrum to save (put in pno of same#)",sav1)
GETINT("Enter 2nd spectrum to save (put in pno of same#)",sav2)
GETINT("Enter 3rd spectrum to save (put in pno of same#)",sav3)
}
GETSTRING("Which intrng file must be used?", text)
GETSTRING("Is the reference integral upfield (u) or downfield (d) of acetate?",ordans)
REXPNO(steno)
SETCURDATA
RPROCNO(phpno)
SETCURDATA
STOREPAR("mi",min)
STOREPAR("pc",pc)
STOREPAR("CURPRIN","Integrals.txt")
/*Create text files to hold integral data*/
/*Global scaling*/
STOREPAR("pscal",0)
if ((fref = fopen(PROCPATH("Reference integral.txt"),"wt")) == 0)
    STOPMSG("Cannot create difflist")
if ((fac = fopen(PROCPATH("Acetate integral.txt"),"wt")) == 0)
    STOPMSG("Cannot create difflist")
    /*No go through each row in turn*/
TIMES(np)
{
RSR(v,wrpno)
RPROCNO(wrpno)
SETCURDATA
sprintf(intdir,"%s\\%s\\%i\\pdata\\%i\\integrals.txt",location,name,expno,procno);
/*Reference the spectrum.*/
/*If the reference peak is split, the program will reference based on the average shift*/
/*of the two peaks in the requested peak picking range which are within ppsens of the largest peak found*/
STOREPAR("f2p",f2pdmso)
STOREPAR("f1p",f1pdmso)
PP
 numPeaks = readPeakList(PROCPATH(0));
maxips=0.0;
    maxpsh=0.0;
    for (i=0; i<numPeaks; i++)
    {
                peakIntensity = getPeakIntensity(i);
                peakFreqHz = getPeakFreqHz(i);
                peakFreqPPM = getPeakFreqPPM(i);
                if (peakIntensity > maxips)
                {
```

```
                    maxips = peakIntensity;
                    maxpsh = peakFreqHz;
                    maxpsp = peakFreqPPM;
                }
    }
    /*Pick most downfield side of multiplet*/
    mintpp=maxips*ppsens;
maxpsp=0.0;
    for (i=0; i<numPeaks; i++)
    {
                                peakIntensity = getPeakIntensity(i);
                                if(peakIntensity>mintpp)
                                {
                peakFreqPPM = getPeakFreqPPM(i);
                peakFreqHz = getPeakFreqHz(i);
                if (peakFreqHz >= maxpsh)
                {
                    maxpsp = peakFreqPPM;
                    maxpsh = peakFreqHz;
                }
    }
                }
/*Flips negative to choose most upfield peak of multiplet*/
for (i=0; i<numPeaks; i++)
    {
                                peakIntensity = getPeakIntensity(i);
                                if(peakIntensity>mintpp)
                                {
                peakFreqPPM = getPeakFreqPPM(i);
peakppmneg=peakFreqPPM*m;
maxpspneg=maxpsp*m;
                if (peakppmneg >= maxpspneg)
                {
                    minpsp = peakFreqPPM;
                }
    }
                }
    freePeakList();
/*References spectrum*/
ppmdif=maxpsp-minpsp;
cent=minpsp+ppmdif*0.5;
FETCHPAR("sf",&sf)
    sfn=sf+(cent-ref)*sf/(1e6);
    STOREPAR("sf",sfn)
/*Integrate spectra, read numbers and store integrals in text file*/
                RMISC("intrng", text)
                LI
fpnt=fopen(intdir, "r");
fgets(dummystr, sizeof(dummystr), fpnt);
while (fgets(dummystr, sizeof(dummystr), fpnt) != NULL)
{
                /*Need to selectively elimate rows, then scan for numbers*/
if(linenum>=5)
{
                (void) sscanf(dummystr,"%lf %lf %lf %lf",
                                        &intnum,&ppmdn,&ppmup,&intgr);
                                        /*DMSO is first, then acetate*/
                                                    if(linenum==5)
                                        {
                                                    if(strcmp(ordans,"d")==0)
                                                    {
                                        fprintf(fref,"%f\n",intgr);
                                                    }
                                                                if(strcmp(ordans,"u")==0)
                                                    {
                                        fprintf(fac,"%f\n",intgr);
                                                    }
                                        }
                                                    if(linenum==6)
                                        {
                                if(strcmp(ordans,"d")==0)
                                                    {
                                        fprintf(fac,"%f\n",intgr);
                                                    }
                                                                if(strcmp(ordans,"u")==0)
                                                    {
                                        fprintf(fref,"%f\n",intgr);
                                                    }
                                        }
                                        intnum=0;
                                        ppmdn=0;
                                        ppmup=0;
                                        intgr=0;
                                        linenum++;
}
                else
{
                                        linenum++;
}
}
linenum=1;
fclose(fpnt);
                /*Save spectra in procnos if requested (will overwrite!!)*/
                if(strcmp(savans,"y")==0)
{
if(v==sav1)
{
WRP(sav1)
}
if(v==sav2)
{
```

```
WRP(sav2)
}
if(v==sav3)
{
WRP(sav3)
}
}
v++;
RPROCNO(phpno)
SETCURDATA
}
END
fclose(fac);
fclose(fref);
QUIT
```

S13.6 AU program to extract areas of acetate and reference from a CSI dataset by lineshape deconvolution (Bruker)

```
/*Bruker AU script for deconvoluting acetate and reference signals in a CSI dataset*/
/*use if overlap with other signals prevents conventional integration*/
/*CSI dataset should have been fully processed in phase-sensitive mode*/
/*Crude chemical shift referencing in F2 also helps*/
/*Open the 2D dataset in Topspin as the active dataset before running this AU*/
/*The script works on Bruker Topspin 3.6.2 but has not been tested on other versions*/
/*Goes through a 2D image and writes file of peak areas*/
/*Will find the area of the most intense peak in specified range for reference and acetate*/
/*After deconvolution, peak areas will be exported to text files stored in procno folder of dataset*/
/*Default peak picking parameters are for DMSO as reference*/
/*This AU is not fully tested and comes without warranty.*/
/*Use kill command if all goes wrong*/
/*Matthew Wallace, 3/2022*/
/*University of East Anglia, matthew.wallace@uea.ac.uk*/
#include <inc/exptUtil>
FILE    *fac,*fref,*frat,*fpnt;
char dconvdir[256],location[256],dummystr[256];
double min=0.1;
double minincac=0.1;
double minincvac;
double minincref=0.1;
double minincvref;
double f2pac=1.8;
double f1pac=2.2;
double f2pref=2.65;
double f1pref=2.8;
double ppsens=0.45;
double fhz=0.0;
double hgt=0.0;
float pc=1;
float azfw=0.5;
double m=-1;
int wrpno=1;
int v=1;
double ppm,wppm,whz,area,chisq,minvref,minvac,ac,ref,refarea,rat,acarea;
int pknum=0;
int steno;
int np=64;
int linenum=1;
int lincnt=6;
int nmpex=1;
int nmiso=1;
int nmisocnt=1;
int nmisoit=0;
int phpno=procno;
int extrctno=phpno+6;
int i, numPeaks,nmpexv;
strcpy(location,disk);
GETCURDATA
steno=expno;
GETSTRING("Enter location of dataset",location)
GETINT("Enter experiment number of 2D dataset",steno)
GETINT("Enter procno of 2D dataset",phpno)
REXPNO(steno)
RPROCNO(phpno)
SETCURDATA
FETCHPAR1("td",&np)
GETINT("Enter number of gradient values",np)
GETDOUBLE("Right acetate peak picking limit/ppm",f2pac)
GETDOUBLE("Left acetate peak picking limit/ppm",f1pac)
GETDOUBLE("Right reference peak picking limit/ppm",f2pref)
GETDOUBLE("Left reference peak picking limit/ppm",f1pref)
GETFLOAT("Enter peak picking sensitivity factor",pc)
GETDOUBLE("Enter satellite sensitivity factor",ppsens)
wrpno=phpno+4;
GETINT("Enter procno to perform deconvolution (blank)",wrpno)
            if ((fac = fopen(PROCPATH("Acetate area.txt"),"wt")) == 0)
   STOPMSG("Cannot create file")
   if ((fref = fopen(PROCPATH("Reference area.txt"),"wt")) == 0)
   STOPMSG("Cannot create file")
   if ((frat = fopen(PROCPATH("Ratio of integral and reference.txt"),"wt")) == 0)
   STOPMSG("Cannot create file")
   TIMES(np)
{
RSR(v,wrpno)
RPROCNO(wrpno)
SETCURDATA
STOREPAR("mi",minvac)
STOREPAR("pc",pc)
STOREPAR("azfw",azfw)
/*Finds max peak in acetate region*/
```

```
STOREPAR("f2p",f2pac)
STOREPAR("f1p",f1pac)
/*Global scaling*/
STOREPAR("pscal",0)
minincvac=minincac;
nmpexv=nmpex;
PP
 numPeaks = readPeakList(PROCPATH(0));
 freePeakList();
while(numPeaks!=nmpexv)
{
STOREPAR("mi",minvac)
PP
/*Checks that expected number of peaks has been found, if not increases PC and repeats peak picking*/
/*If are in fact less than expected number will just pick noise, but this does not matter*/
numPeaks = readPeakList(PROCPATH(0));
freePeakList();
if(numPeaks>nmpex)
{
minvac=minvac+minincvac;
nmisocnt++;
if(nmisocnt>=20)
{
minincvac=minincvac/10;
nmisocnt=0;
/*Exit loop 0.001 seems sensible limit*/
if(minincvac<0.001)
{
nmpexv=numPeaks;
}
}
}
if(numPeaks<nmpex)
{
minvac=minvac-minincvac;
nmisocnt++;
if(nmisocnt>=20)
{
minincvac=minincvac/10;
nmisocnt=0;
/*Exit loop*/
if(minincvac<0.001)
{
nmpexv=numPeaks;
}
}
}
}
  freePeakList();
nmisocnt=1;
nmpexv=nmpex;
/*Having found peaks, now write peak positions into peaklist file for deconvolution*/
DATASET2(name,expno,procno+1000,disk,user)
PPP
     /*Now do deconvolution*/
MDCON
sprintf(dconvdir,"%s/%s/%i/pdata/%i/integrals.txt",location,name,expno,procno);
fpnt=fopen(dconvdir, "r");
fgets(dummystr, sizeof(dummystr), fpnt);
while (fgets(dummystr, sizeof(dummystr), fpnt) != NULL)
{
                /*Need to selectively eliminate rows from dconpeaks, then scan for numbers*/
if(linenum>=9)
{
(void) sscanf(dummystr,"%lf %lf %lf %lf %lf %lf %lf",
                                                        &ppm,&fhz,&wppm,&whz,&hgt,&area,&chisq);

if(linenum==10)
{
fprintf(fac,"%f\n",area);
acarea=area;
}
linenum++;
}
else
{
                                                        linenum++;
}
}
linenum=1;
                fclose(fpnt);
                /*Now do same for ref region*/
STOREPAR("f2p",f2pref)
STOREPAR("f1p",f1pref)
minincvref=minincref;
nmpexv=nmpex;
PP
 numPeaks = readPeakList(PROCPATH(0));
 freePeakList();
while(numPeaks!=nmpexv)
{
STOREPAR("mi",minvref)
PP
/*Checks that expected number of peaks has been found, if not increases PC and repeats peak picking*/
/*If are in fact less than expected number will just pick noise, but not matter*/
numPeaks = readPeakList(PROCPATH(0));
freePeakList();
if(numPeaks>nmpex)
{
minvref=minvref+minincvref;
nmisocnt++;
```

```
if(nmisocnt>=20)
{
minincvref=minincvref/10;
nmisocnt=0;
/*Exit loop 0.001 seems sensible limit*/
if(minincvref<0.001)
{
nmpexv=numPeaks;
}
}
}
if(numPeaks<nmpex)
{
minvref=minvref-minincvref;
nmisocnt++;
if(nmisocnt>=20)
{
minincvref=minincvref/10;
nmisocnt=0;
/*Exit loop*/
if(minincvref<0.001)
{
nmpexv=numPeaks;
}
}
}
}
  freePeakList();
nmisocnt=1;
nmpexv=nmpex;
PPP
     /*Now do deconvolution*/
MDCON
sprintf(dconvdir,"%s/%s/%i/pdata/%i/integrals.txt",location,name,expno,procno);
fpnt=fopen(dconvdir, "r");
fgets(dummystr, sizeof(dummystr), fpnt);
while (fgets(dummystr, sizeof(dummystr), fpnt) != NULL)
{
                /*Need to selectively elimate rows from dconpeaks, then scan for numbers*/
if(linenum>=9)
{
(void) sscanf(dummystr,"%lf %lf %lf %lf %lf %lf %lf",
                                                   &ppm,&fhz,&wppm,&whz,&hgt,&area,&chisq);
if(linenum==10)
{
fprintf(fref,"%f\n",area);
refarea=area;
}
linenum++;
}
else
{
                                                   linenum++;
}
}
linenum=1;
                fclose(fpnt);
                rat=acarea/refarea;
                fprintf(frat,"%f\n",rat);
                RPROCNO(phpno)
                SETCURDATA
                v++;
}
                END
                fclose(frat);
                fclose(fac);
                fclose(fref);
                QUIT
```

S13.7 AU program to shim and find water suppression frequency when running under IconNMR (Bruker)

```
/*Bruker AU script for shimming, finding water supression frequency*/
/*and running CSI experiment through IconNMR*/
/*create macro for reading in default shim file*/
/*edmac rshim, then in macro text: rsh nameofshimfile*/
/*create macro for topshim on 1h:*/
/*edmac t1h, then in macro text: topshim 1h lockoff o1p=4.85 ordmax=3 convcomp*/
/*create a 1 scan proton parameter set (low rg) to find o1p of water, called H2O_SS*/
/*Remember to change peak picking regions in this PAR set to cover the expected range for water signal*/
/*create a CSI parameter set called 1hcsi (or equivalent), this runs the csi experiment*/
/*set parameter AUNM in this par set to the name of this script*/
/*Then add the par set onto your list of experiments in the IconNMR config menu*/
/*when running through Icon, disable locking and shimming by choosing the "IconNMR not responsible" option*/
/*The script works on Bruker Topspin 3.6.2 but has not been tested on other versions*/
/*This AU is not fully tested and comes without warranty.*/
/*Matthew Wallace, 5/2022*/
/*University of East Anglia, matthew.wallace@uea.ac.uk*/
 float peakFreqHz, peakFreqPPM, peakIntensity, maxpsh, maxpsp, maxips;
    char path[PATH_MAX];
double sf, sfo1;
int pscal_save, i, numPeaks;

GETCURDATA
/*Can set numbr of scans for water suppression in ICON*/
RPAR("H2O_SS","all")
/*STOREPAR("RG",1)*/
XMAC("rshim")
```

```
sleep(60);
XMAC("t1h")
sleep(90);
ZG
ERRORABORT
EF
ERRORABORT
APK
/*SREF*/
FETCHPAR("PSCAL",&pscal_save)
STOREPAR("PSCAL",0)
PP
ERRORABORT
  strcpy(path, PROCPATH(0));
    numPeaks = readPeakList(path);

    maxips=0.0;
    maxpsh=0.0;
    for (i=0; i<numPeaks; i++)
    {
            peakIntensity = getPeakIntensity(i);
            peakFreqHz = getPeakFreqHz(i);
            peakFreqPPM = getPeakFreqPPM(i);
            if (peakIntensity > maxips)
            {
                maxips = peakIntensity;
                maxpsh = peakFreqHz;
                maxpsp = peakFreqPPM;
            }
    }
    freePeakList();

    FETCHPAR("SF",&sf);
    sfo1 = sf + maxpsh * 1.0e-6;
    STOREPAR("SFO1",sfo1);
    SETCURDATA
/*RGA can go wrong for water suppression*/
RPAR("1hcsi","all")
STOREPAR("SFO1",sfo1)
ZG
QUIT
```

S13.8 Processing procedure for Mnova 14.2.0

To import a CSI dataset into Mnova, run processing AU (S13.2). Open 2rr file in procno directory of NMR dataset in Mnova. Set up and run a processing template as shown below to automatically phase, reference and baseline correct the data.

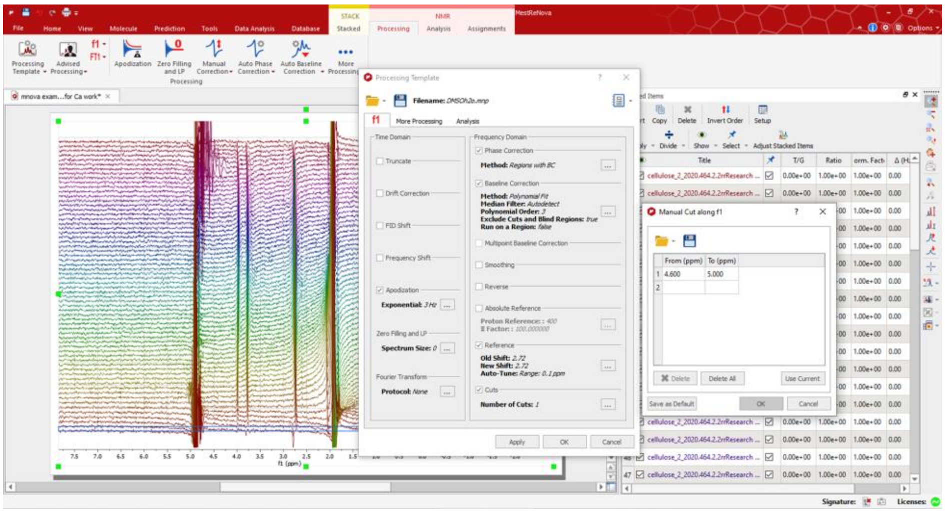

Extract peak positions and integrals using Data Analysis module

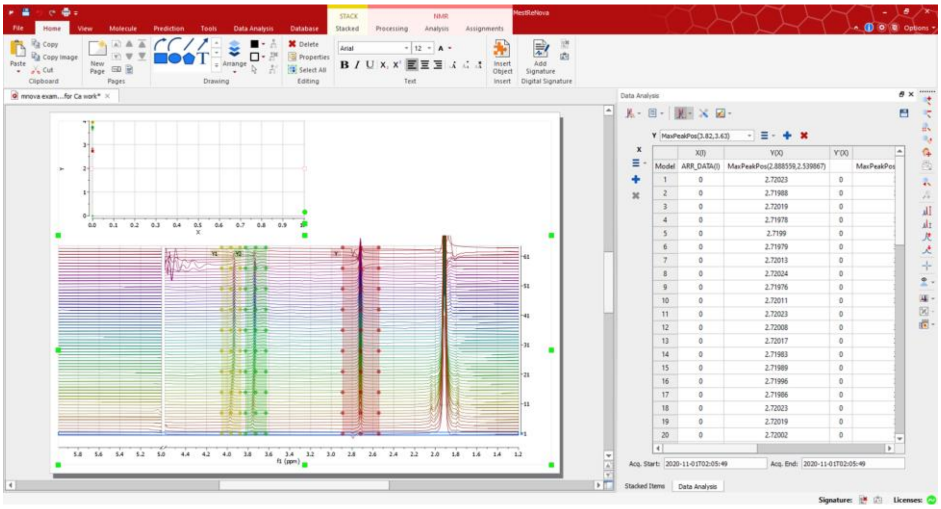

To extract peak areas, create line fitting region and fit all rows in the CSI dataset. Then run script below to export areas:

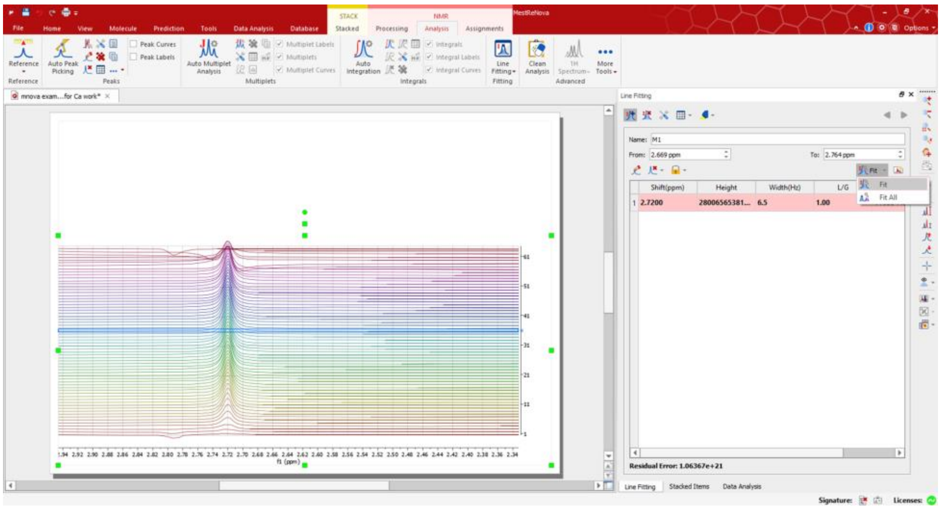

```
/*******************************************************************************************
For finding area of singlet peak in stacked plot from a Chemical Shift Imaging (CSI) dataset
Matthew Wallace, 3/2022
University of East Anglia, matthew.wallace@uea.ac.uk

Define line fitting area with new fit region (clear all previous line fitting regions)
Click fit to fit all spectra in the stacked CSI dataset
Save this script in Mnova as PeakArea, and Run this script

Script will find the biggest area of a peak in a spectrum within the defined region and put in txt document
Area can then be copied and pasted into Excel

Based on Mnova script exportFitRegions
Copyright (C) 2014 Mestrelab Research S.L. All rights reserved.

This file is part of the Mnova scripting toolkit.

Authorized users of Mnova Software may use this file freely, but this file is provided AS IS
with NO WARRANTY OF ANY KIND, INCLUDING THE WARRANTY OF DESIGN, MERCHANTABILITY AND FITNESS
FOR A PARTICULAR PURPOSE.
*******************************************************************************************/

/*globals settings, Dir, FileDialog, File, TextStream, Application, NMRSpectrum, print, Peak, MnUi*/
/*jslint plusplus: true, indent: 4*/

function PeakArea() {
	"use strict";

	function fitRegionToStream(aFitRegion, aFileStream, aNMRPeaks) {
/*			aFileStream.writeln("Area");*/
			var p, peak, tst, big
				tst=0;
				big=0;
				fitPeaksIds = aFitRegion.peaks;
			for (p = 0; p < fitPeaksIds.length; p++) {
				peak = new Peak(aNMRPeaks.byId(fitPeaksIds[p]));
```

```
                    tst=peak.integral;
                    if(tst>big)
                    {
                    big=peak.integral;
                    }
                    }
                    aFileStream.write(big, "\n");
        }

    var fout, sout, spc, peakList, fitRegions, fr, oldCurSpecIndex, i,
                dirSettingsKey = "PeakArea/LastDir",
                saveDir = settings.value(dirSettingsKey, Dir.home()),
                dw = Application.mainWindow.activeDocument,
                spectra = dw.itemCount("NMR Spectrum"),
                specIndex = 0,
                fileName = FileDialog.getSaveFileName("ASCII Files (*.txt)", "", saveDir);

    if (!fileName.length) {
                return;
        }

    fout = new File(fileName);
    settings.setValue(dirSettingsKey, fout.absDirPath);
    if (!fout.open(File.WriteOnly)) {
                throw "Impossible to open file";
        }
    sout = new TextStream(fout);
    sout.precision = 10;

    while (specIndex < spectra) {
                spc = new NMRSpectrum(dw.item(specIndex, "NMR Spectrum"));
                specIndex++;
                if (!spc.isValid()) {
                    throw "Invalid Spectrum";
                }
                oldCurSpecIndex = spc.curSpecIndex;
                for (i = 0; i < spc.specCount; i++) {
                    spc.curSpecIndex = i;
                    peakList = spc.peaks();
                    fitRegions = spc.fitRegions();
                    print(fitRegions);
                    for (fr = 0; fr < fitRegions.length; fr++) {
                        fitRegionToStream(fitRegions[fr], sout, peakList);
                    }
                }
                spc.curSpecIndex = oldCurSpecIndex;
    }
    fout.close();
}

if (this.MnUi && MnUi.scripts_nmr) {
        MnUi.scripts_nmr.scripts_nmr_ExportASCIIFitRegions = PeakArea;
}
```
